# Synthesis and Characterization of Bis-Triazolyl-Pyridine Derivatives as Noncanonical DNA-Interacting Compounds

**DOI:** 10.3390/ijms222111959

**Published:** 2021-11-04

**Authors:** Anna Di Porzio, Ubaldina Galli, Jussara Amato, Pasquale Zizza, Sara Iachettini, Nunzia Iaccarino, Simona Marzano, Federica Santoro, Diego Brancaccio, Alfonso Carotenuto, Stefano De Tito, Annamaria Biroccio, Bruno Pagano, Gian Cesare Tron, Antonio Randazzo

**Affiliations:** 1 Department of Pharmacy, University of Naples Federico II, Via D. Montesano 49, 80131 Naples, Italy; anna.diporzio@unina.it (A.D.P.); jussara.amato@unina.it (J.A.); nunzia.iaccarino@unina.it (N.I.); simona.marzano@unina.it (S.M.); federica.santoro@unina.it (F.S.); diego.brancaccio@unina.it (D.B.); alfonso.carotenuto@unina.it (A.C.); bruno.pagano@unina.it (B.P.); 2 Department of Pharmaceutical Sciences, University of Piemonte Orientale, Largo Donegani 2/3, 28100 Novara, Italy; ubaldina.galli@uniupo.it; 3 Oncogenomic and Epigenetic Unit, IRCCS-Regina Elena National Cancer Institute, 00144 Rome, Italy; pasquale.zizza@ifo.gov.it (P.Z.); sara.iachettini@ifo.gov.it (S.I.); annamaria.biroccio@ifo.gov.it (A.B.); 4 Molecular Cell Biology of Autophagy, The Francis Crick Institute, 1 Midland Road, London NW1 1AT, UK; stefano.de-tito@crick.ac.uk; 5 Institute of Experimental Endocrinology and Oncology, National Research Council, 80131 Naples, Italy

**Keywords:** G-quadruplex, i-motif, bis-triazolyl-pyridine derivatives, click chemistry, biophysical study, immunofluorescence microscopy

## Abstract

Besides the well-known double-helical conformation, DNA is capable of folding into various noncanonical arrangements, such as G-quadruplexes (G4s) and i-motifs (iMs), whose occurrence in gene promoters, replication origins, and telomeres highlights the breadth of biological processes that they might regulate. Particularly, previous studies have reported that G4 and iM structures may play different roles in controlling gene transcription. Anyway, molecular tools able to simultaneously stabilize/destabilize those structures are still needed to shed light on what happens at the biological level. Herein, a multicomponent reaction and a click chemistry functionalization were combined to generate a set of 31 bis-triazolyl-pyridine derivatives which were initially screened by circular dichroism for their ability to interact with different G4 and/or iM DNAs and to affect the thermal stability of these structures. All the compounds were then clustered through multivariate data analysis, based on such capability. The most promising compounds were subjected to a further biophysical and biological characterization, leading to the identification of two molecules simultaneously able to stabilize G4s and destabilize iMs, both in vitro and in living cells.

## 1. Introduction

**Figure 1:**
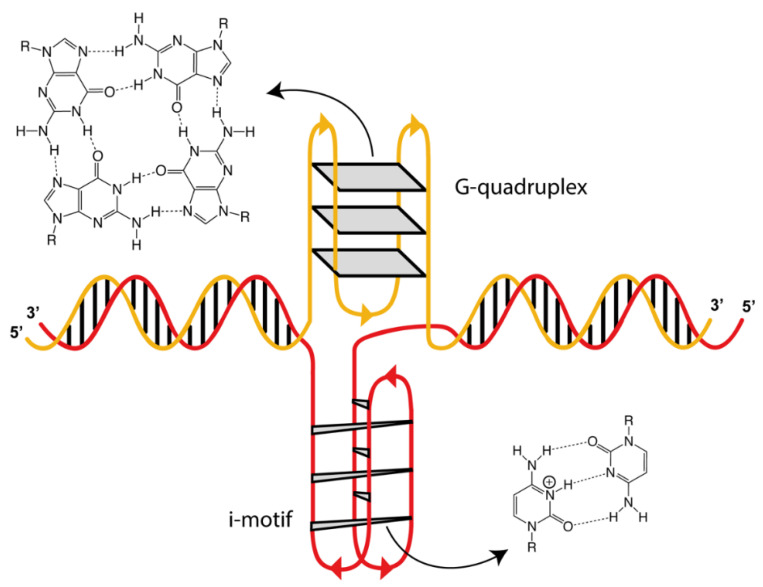
Noncanonical DNA secondary structures: G-quadruplex (G4) and i-motif (iM). **Top left**: a G-tetrad; **bottom right**: a hemi-protonated cytosine-cytosine (C-C^+^) base pair.

The canonical double helix is the most widely recognized genomic DNA structure. However, DNA is structurally dynamic and able to adopt a number of alternative secondary structures, such as cruciforms, triplexes, G-quadruplexes, and i-motifs [1,2,3]. G-quadruplex (G4) structures, four-stranded helical complexes that can arise from guanine-rich sequences, are among the most extensively studied noncanonical DNA secondary structures [4]. The core scaffold of a G4 is the G-tetrad (Figure 1), a cyclic array of four guanines connected by eight Hoogsteen hydrogen bonds. Two or more G-tetrads can self-associate into vertical stacks giving rise to G4s, which are extremely stable structures, although the stability of a G4 structure depends on many factors, including the length and sequence composition of the G-rich motif, the nature of the binding cations, and the folding topology [5,6,7,8]. Indeed, G4s can adopt a wide variety of topologies, depending on the combinations of strand orientation and length and composition of the loops (the intervening sequences between the G-rich repeats) [9,10]. DNA sequences that can fold into G4 structures are broadly distributed across the human genome and mainly located at telomeres, gene promoters, and introns [11,12]. Due to such a high genomic prevalence, G4s have been speculated to play a role in several biological processes. Indeed, they may offer telomere protection and block replication fork progression [4], while in gene promoters, they may represent a physical obstacle in the way of the RNA polymerase machinery, thus affecting the expression of the downstream gene [13].

The strand complementary to a G4-forming DNA motif is a C-rich sequence that can fold in another four-stranded structure called the i-motif (iM or i-DNA) [14]. This is composed of two head-to-tail, intercalated, parallel-stranded duplexes held together by hemi-protonated cytosine-cytosine (C-C^+^) base pairs (Figure 1). Very recently, evidence for iM formation in cells has been provided by NMR experiments [15] and by the discovery of an antibody that specifically binds iM structures in the nuclei of human cells [16]. Analogously to G4s, the presence of iM structures may affect gene transcription and telomere biology [2]. Interestingly, the formation of iM structures is cell-cycle dependent, peaking at the late G1 phase [16], whereas G4 formation is maximal during the S phase [17]. This suggests that iMs and G4s might play different roles in regulating gene expression and transcription.

Many studies, focused on disentangling the biological roles of G4 and iM structures, demonstrate that such structures can be considered as molecular targets in cancer therapies [14,18,19], leading to significant therapeutic advantages in the treatment of cancer. For example, direct evidence of the role of DNA G4s in gene regulation has been provided, showing that oncogene promoter activity is generally repressed using G4s stabilizers, while destabilization of G4s generally leads to increased gene transcription [20]. On the other hand, constant efforts are still underway to fully decipher the specific biological functions of iM structures. In this contest, the scientific community has also made big efforts to find small molecules able to recognize G4 and iM structures. More than a thousand compounds that bind and stabilize G4 structures have been found so far. Many of them display considerable selectivity for G4s over single-stranded and double-stranded DNA, and some of them are also able to discriminate among distinct G4 folding topologies. Compared to the well-documented examples of G4 ligands, the discovery of specific iM-targeting compounds is much more limited [21,22].

The design of small molecules able to interact with G4s is usually based on the following requisites: (i) the presence of a (hetero)-aromatic system that gives π-stacking interactions with planar G-tetrads and flanking bases, (ii) a V-shaped form to maximize the interaction with the G4, (iii) two or three cationic side chains able to participate in ionic interactions with the phosphate backbone of loops and grooves of G4 structures. On the other hand, loop binding and recognition of hemi-protonated C-C^+^ base pairing seem to be the most favored binding modes of iM ligands [23,24].

To increase the number of putative noncanonical DNA-targeting compounds, medicinal chemists are in continuous search for the use of synthetic strategies able to give access to as many compounds as possible in a short period of time. In this context, over the years, the so-called click chemistry reaction, namely the Cu(I)-catalyzed Huisgen 1,3-dipolar cycloaddition between azides and alkynes (CuAAC) [25], has gained considerable interest in medicinal chemistry in general, and in the design of G4 binding ligands in particular [26]. The CuAAC exclusively generates a 1,4-disubstituted 1,2,3-triazole. This reaction is operationally simple, regioselective, modular, and bio-orthogonal, providing high yields of the products. Moreover, it can be performed in aqueous media under mild conditions and the azide and alkyne building blocks are either commercially available or easily synthesizable. In addition, the resulting 1,2,3-triazole system is stable under physiological conditions as it is resistant to hydrolysis and oxidation-reduction [27,28].

In 2006, Neidle’s group described, for the first time, the use of click chemistry for the synthesis of G4-stabilizing ligands [29]. Afterward, this reaction has been employed to functionalize different aromatic/heteroaromatic moieties with appropriate side chain appendages to form novel molecular scaffolds that displayed efficient binding properties to G4s of different topologies [30,31,32].

Here, inspired by pyridostatin, a potent G4-binder based on an *N*,*N*′-bis(quinolinyl)pyridine-2,6-dicarboxamide scaffold [33,34,35] which has also been shown to interact with iM DNA (although without significant effects on the thermal stability of such structure) [36], a library of bis-triazolyl-pyridine derivatives has been generated. Then, the ability of such compounds of interacting with G4 and iM structures has been investigated both from a biophysical and biological point of view. In particular, we exploited the Hantzsch multicomponent reaction [37,38] to generate a central pyridine scaffold with two ester groups, which can act as potential arms ready to be functionalized. The resulting heterocyclic ring can work as a planar aromatic platform able to produce π-stacking interactions with the nucleobases, whereas the ester groups can be converted into azides. Then, a click chemistry reaction between azides and terminal alkynes containing a tertiary amine was carried out to install two cationic side chains able to interact with the negatively charged phosphate groups to stabilize the interaction with the nucleic acid.

The synergistic effect of a multicomponent reaction and a click chemistry functionalization allowed for the generation of a library of 31 potential G4 and/or iM ligands thanks to the extensive modulation of the basic side chains, which is well-known to be crucial to impart the required binding selectivity.

The synthesized bis-triazolyl-pyridines were preliminarily screened for their ability to interact with G4 and/or iM DNAs through circular dichroism (CD) spectroscopy and clustered based on their capability to affect the thermal stability of the investigated DNA sequences by employing the Principal Component Analysis (PCA) technique. The ability of the best-selected compounds to interact with noncanonical DNAs was further investigated by using NMR and fluorescence spectroscopies. Finally, the proficiency of such compounds to modulate G4 and iM structures formation in living cells was assessed.

## 2. Results and Discussion

### 2.1. Chemistry

**Scheme 1:**
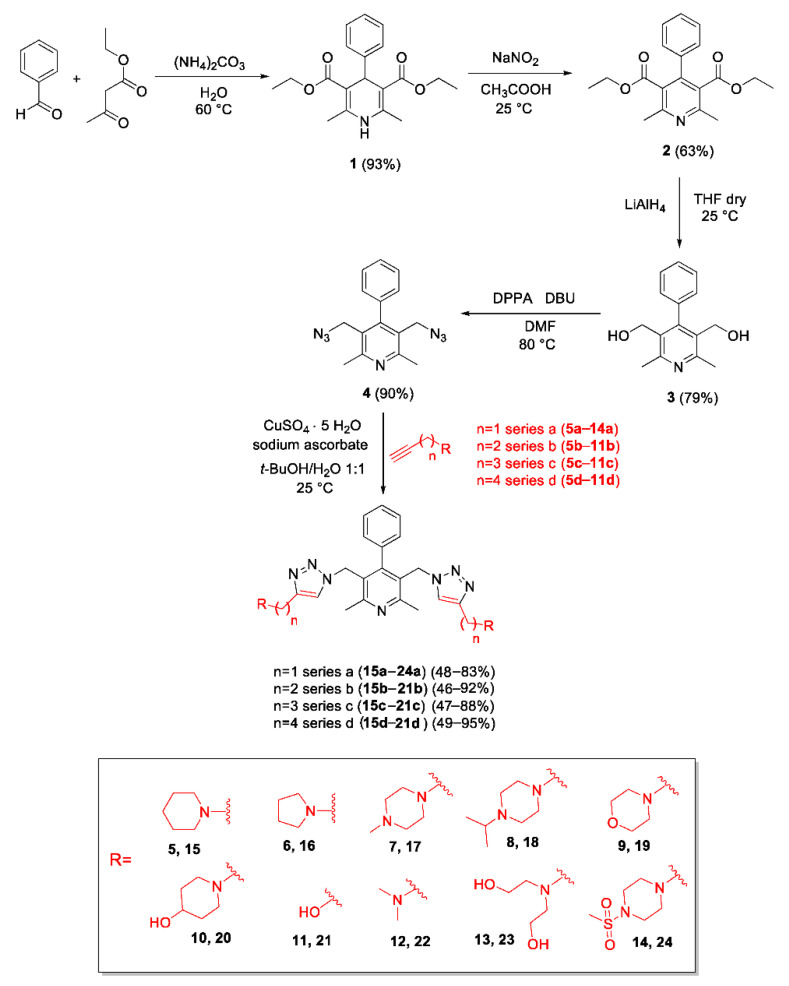
Synthesis of bis-triazolyl-pyridines **15a**–**24a**, **15b**–**21b**, **15c**–**21c**, **15d**–**21d**.

The synthetic route followed for the preparation of the putative noncanonical DNA-interacting compounds is depicted in Scheme 1. In brief, the pyridine scaffold was obtained reacting benzaldehyde, ethyl acetoacetate, and ammonium carbonate in water at 60 °C [39]. The dihydropyridine (**1**) obtained was then aromatized to give **2** via oxidation with sodium nitrite in glacial acetic acid at room temperature (RT). The two ester groups were then reduced to alcohol using LiAlH_4_. The diol **3** was transformed into the diazide **4** using the DPPA, DBU protocol. The diazide **4** was reacted with twenty-seven terminal alkynes (see Supplementary Materials, General Procedures for the Synthesis of Alkynes and Schemes S1–S4), which contain at least one tertiary nitrogen atom, and with four commercial alkynes containing hydroxyl groups, by using the CuAAC protocol [25]. In particular, click reactions were carried out with four series of alkynes of different lengths: series a (**5a**–**14a**), series b (**5b**–**11b**), series c (**5c**–**11c**), series d (**5d**–**11d**) containing one, two, three or four methylene groups, respectively. A library of 31 bis-triazolyl-pyridines (series a **15a**–**24a**, series b **15b**–**21b**, series c **15c**–**21c**, series d **15d**–**21d**) were thus obtained and the compounds were tested for their ability to interact with G4 and iM DNA structures. The identity of the new compounds was established by their FT-IR, ^1^H-NMR, ^13^C-NMR, MS, and elemental analyses.

### 2.2. Circular Dichroism Screening and Selection of the Most Promising Compounds

The 31 synthesized bis-triazolyl-pyridines were preliminarily screened for their ability to interact with G4 and iM DNAs through circular dichroism (CD) spectroscopy [40]. Various G-rich sequences that can form parallel, antiparallel, and hybrid G4 structures and their iM-forming C-rich counterparts were used in these experiments. In particular, we employed the G-/C-rich motifs from the promoter regions of BCL-2 (*Bcl-2 G4* and *Bcl-2 iM*) [41,42] and c-MYC (*c-Myc G4* and *c-Myc iM*) oncogenes [43,44], as well as the G-rich 23-mer (*Tel_23_ G4)* and the C-rich 22-mer (*hTeloC iM*) truncations of the human telomeric DNA sequence [45,46,47]. Moreover, a 27-mer hairpin-duplex-forming sequence (*Hairpin*) was also used to evaluate the selectivity of the compounds for noncanonical DNAs over duplex DNA. DNA samples were prepared in the appropriate buffers (see Materials and Methods section) at pH 7.0 for the G-rich and hairpin-duplex-forming sequences, and at pH 5.0 for the C-rich sequences since the formation of iM structures requires cytosine protonation to form the C-C^+^ base pair.

The overall topology adopted by each investigated DNA sequence was first verified by CD measurements (Figures S1–S4). The *c-Myc G4*-forming sequence adopted a parallel G4 conformation, showing the characteristic positive band at around 260 nm and negative band at ~240 nm in its CD spectrum. The presence of an additional band at ~290 nm in the CD spectrum of *Bcl-2 G4* indicates the formation of a mixed parallel/antiparallel conformation. On the other hand, the *Tel_23_ G4* sequence showed a CD spectrum having two positive bands at 290 and 270 nm and a weak negative band at 240 nm, consistent with the formation of a hybrid [3+1] G4 folding topology. As for *Bcl-2 iM, c-Myc iM*, and *hTeloC iM*, all of them exhibited a CD spectrum with a positive band at 288 nm and a negative one at around 260 nm, which are characteristic values of iM formation [48], while the *Hairpin* showed a positive band at around 280 nm and a negative one at ~250 nm in the CD spectrum, confirming duplex formation (Figure S4).

Additional CD spectra were acquired to explore the potential of the 31 bis-triazolyl-pyridines to alter the native folding topology of the investigated DNAs. Upon addition of an excess of compounds (10 molar equiv relative to the DNA), no relevant variations of DNA chiroptical signals were observed, suggesting overall preservation of the DNA structure in the presence of the compounds (Figures S1–S4). Then, their DNA stabilizing/destabilizing properties were evaluated by CD-melting experiments measuring the compound-induced change in the apparent melting temperature (Δ*T*_1/2_) either of G4, iM, or duplex structures (Table 1 and Figures S5–S8).

**Figure 2:**
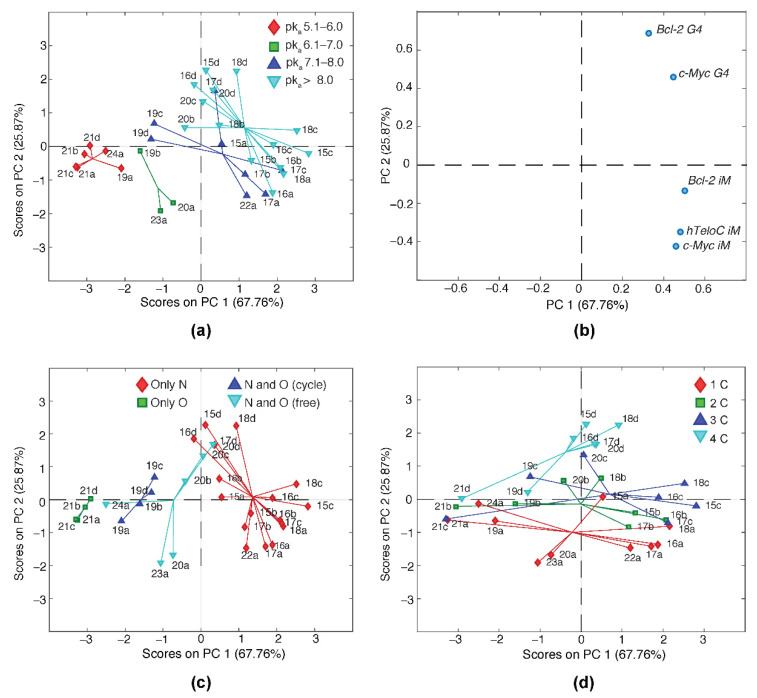
Output of the PCA model performed on the Δ*T*_1/2_ values dataset of the 31 compounds tested on *Bcl-2 G4*, *c-Myc G4*, *Bcl-2 iM*, *c-Myc iM,* and *hTeloC iM* DNAs. PC1/PC2 scores plot colored according to the (**a**) pK_a_ value corresponding to the conjugated acid of the strongest basic group in each molecule (**c**) presence/absence of both nitrogen and oxygen atoms in the side chains, and (**d**) length of the R side chain (from 1 up to 4 carbons); (**b**) PC1/PC2 loadings plot.

Considering a large number of Δ*T*_1/2_ values to be compared (31 compounds tested on 7 DNA sequences give a total of 217 values of Δ*T*_1/2_), a multivariate data analysis tool, such as Principal Component Analysis (PCA) [49], was employed to explore the data set. In this way, similarities and differences in the behavior of the investigated compounds could be detected and correlated to their structural features. PCA is an unsupervised method that allows the reduction in the dimensionality of a data set, providing a visual representation of the major variance in the data. In particular, the original variables (5 DNA sequences) were transformed into 2 new uncorrelated variables termed Principal Component 1 (PC1), which explains 67.76% of the total variance, and Principal Component 2 (PC2) explaining 25.87% of the variance. PC1 and PC2 are visualized both in a scores plot (Figure 2a,c,d), where the compounds characterized by a similar behavior are clustered together and colored according to their structural features and in a loadings plot (Figure 2b) that indicates the DNA sequences responsible for the distribution of the compounds observed in the scores plot. Therefore, by employing this technique, we could cluster the 31 compounds based on their capability to affect the thermal stability of the investigated DNA sequences.

*Hairpin* and *Tel_23_ G4* were excluded from the analysis since their thermal stability was not significantly affected by any of the tested compounds (|Δ*T*_1/2_| < 4 °C). Therefore, the PCA was performed including two G4-forming sequences (*Bcl-2 G4* and *c-Myc G4*) and three iM-forming sequences (*hTeloC iM*, *c-Myc iM*, and *Bcl-2 iM*) for a total of 5 DNA sequences. Moreover, we decided to take into account the Δ*T*_1/2_ absolute values considering that most of the compounds destabilized the iM structures (negative Δ*T*_1/2_) while stabilizing the G4 structures. Figure 2 shows the scores and loadings plots generated by the PCA model. The fact that all the DNA sequences are clustered together on the very right side of the loadings plot (Figure 2b) suggests that the compounds that lie on the right side of the scores plot (Figure 2a) are characterized by higher |Δ*T*_1/2_| values compared to the ones situated on the left side. Interestingly, by coloring each compound according to the pK_a_ value corresponding to the conjugate acid of the strongest basic group in the molecule (see Table S1 and Figure S9 for details), we could observe the presence of a trend along PC1, which goes from the left (lower pK_a_ values) to the right (higher pK_a_ values). This means that the compounds that least affect the thermal stability of the investigated DNA structures are the ones that are less prone to carry a positive charge (under the experimental conditions), which, in turn, is fundamental for the interaction with the negatively charged DNA backbone [50,51]. Then, in order to understand the common structural features shared by the compounds able to interact with the DNA structures, the samples were colored according to the chemical features of the side chain (R). Thus, they were divided into different classes where R (i) contains only nitrogen; (ii) contains only oxygen; (iii) contains both nitrogen and oxygen with the latter being closed in a cycle; and (iv) contains both nitrogen and oxygen with the latter being present as a free hydroxyl group. Figure 2c shows the same scores plot reported in Figure 2a but colored according to these new classes and it is evident that both the lack of nitrogen and the presence of oxygen reduce the capability of the compounds to stabilize/destabilize the DNA structures. Indeed, the compounds that most affect the stability of the DNA structures are those containing only nitrogen atoms in the R chain.

On the other hand, PC2 (28.87% of the total variance) shows the distribution of the compounds according to their effect on G4 rather than on the iM structures. In particular, **16a**, **17a**, **20a**, **22a**, and **23a**, located in the lower part of the scores plot where the iM-forming sequences are placed (in the corresponding area of the loadings plot), affect more significantly the thermal stability of the iMs rather than G4 structures. On the contrary, compounds **15d**, **16d**, **17d**, **18d**, **20d,** and **20c**, located in the upper part of the plot, where the G4-forming sequences are placed (in the corresponding area of the loadings plot), preferentially affect the thermal stability of the G4 over the iM structures. In order to understand which structural feature could lead to such a selective effect on a DNA structure over another, we decided to label the compounds in the scores plot according to the length of the R side chain, which goes from 1 carbon (series a) up to 4 carbons (series d) (Figure 2d). Interestingly, we observed that the longer is the carbon chain, the higher is the ability to stabilize the G4 structures. The effect of the side chain length is observable only for the compounds that do affect the thermal stability of the investigated structures (right part of the plot) while it is not evident for the rest of the molecules. This confirms once again that the presence of positive charges is fundamental for the interaction with the DNA, while the length of the side chain could modulate compound selectivity. Interestingly, there are two compounds (**15c** and **18c**) that are located at the very right side of the PC1 axis, and thus characterized by high Δ*T*_1/2_ values, while being in the middle region of the PC2 axis, meaning that they are able to affect the thermal stability of both iM and G4 structures. In particular, they can stabilize the G4 structures while destabilizing the iM ones. This is a very interesting aspect from a biological point of view since previous studies have reported that G4 and iM structures may exert opposite roles in controlling gene transcription: while G4 ligand-induced stabilization generally blocks gene expression, stabilization of iMs seems to show transcription activating capabilities [36,52].

### 2.3. Nuclear Magnetic Resonance (NMR) Studies

To get information about the binding mode of **15c**, **18c**, **20a,** and **23a** to *c-Myc G4* and *hTeloC iM*, 1D ^1^H-NMR experiments were performed. We focused on these compounds since able to affect the thermal stability of both iM and G4 structures (**15c** and **18c**) or exclusively of the iMs (**20a** and **23a**). In addition, **21d** was also employed to have a comparison with a compound unable to affect the stability of any of the investigated DNAs. Particularly, wanting to perform a comparative study on the binding mode and affinity of the most promising compounds towards the investigated G4/iMs and needing to use acidic conditions to guarantee iM folding, we performed NMR and fluorescence titration experiments under the same experimental conditions (10 mM potassium phosphate buffer, pH 5.0, for the studied G4 and 10 mM sodium phosphate buffer, pH 5.0, for the iMs).

**Figure 3:**
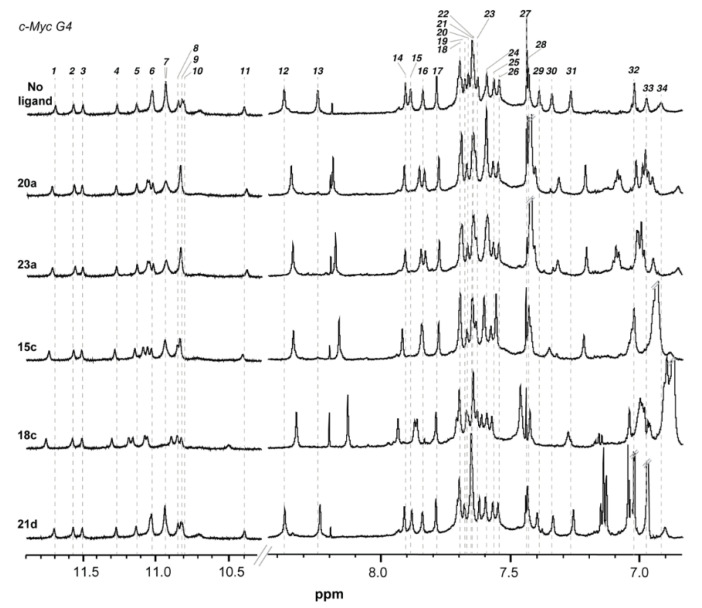
^1^H-NMR spectra of *c-Myc G4* in the absence and presence of compounds **20a**, **23a**, **15c**, **18c,** and **21d**. See Table S2 for signal assignment. The NMR sample was prepared at 100–150 μM final oligonucleotide concentration in 10 mM potassium phosphate buffer (pH 5.0).

**Figure 4:**
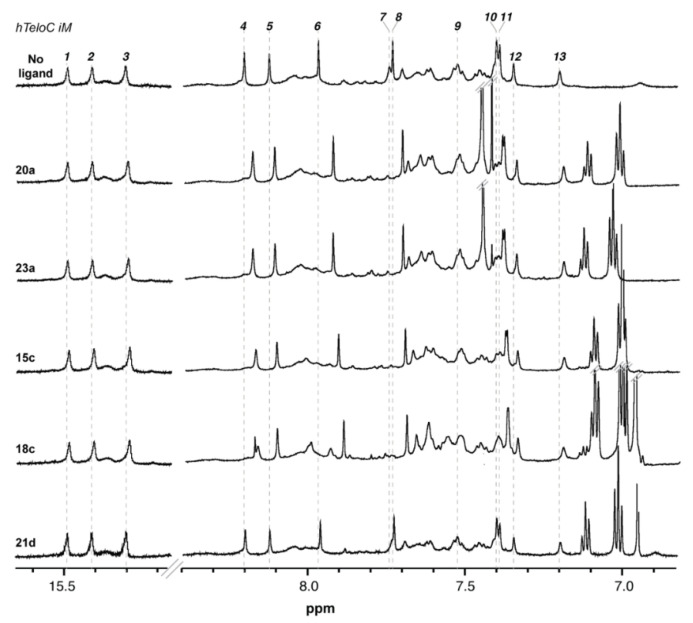
^1^H-NMR spectra of *hTeloC iM* in the absence and presence of compounds **20a**, **23a**, **15c**, **18c**, and **21d**. See Table S3 for signal assignment. The NMR sample was prepared at 100–150 μM final oligonucleotide concentration in 10 mM sodium phosphate buffer (pH 5.0).

The spectral regions of the imino and aromatic protons of *c-Myc G4* and *hTeloC iM* in the absence and presence of compounds (2 molar equiv) are shown in Figure 3 and Figure 4, respectively. According to the literature, the *c-Myc G4* sequence forms, under the experimental conditions used, a single G4 conformation characterized by 12 well-resolved imino proton peaks, corresponding to the 12 guanines involved in the three G-tetrad planes [43]. On the other hand, *hTeloC iM* folds in an iM structure characterized by 3 well-resolved imino proton peaks that correspond to the 6 intercalated C-C^+^ pairs [47].

No relevant shift of both the imino and aromatic signals of *c-Myc G4* and *hTeloC iM* was observed upon the addition of **21d** (Figure 3 and Figure 4, and Tables S2 and S3). Conversely, **15c**, **18c**, **20a,** and **23a** mainly affected the imino protons of *c-Myc G4,* as well as the aromatic protons of both *c-Myc G4* and *hTeloC iM* (Figure 3 and Figure 4, and Tables S2 and S3, respectively). As for *c-Myc G4*, not negligible chemical shift changes were observed for the imino (H1) protons of G16 belonging to the 5′ G-tetrad (G7−G11−G16−G20), as well as for those of guanines involved in the 3′ G-tetrad (G9−G13−G18−G22). At the same time, the most perturbed aromatic protons turned out to be those of A15, located in the double nucleotide loop (T14A15) connecting G13 and G16, and of A25 which stacks over the 3′ G-tetrad, specifically, with residues G9 and G22. Overall, these results suggest a common end-stacking binding mode for **15c**, **18c**, **20a**, and **23a** to the 3′ G-tetrad of *c-Myc G4*, which is also combined with loop binding.

Conversely, the imino proton signals of *hTeloC iM* were not affected by compounds **15c**, **18c**, **20a**, and **23a**. However, significant chemical shift changes of aromatic protons were observed especially for those residues located in the loops of *hTeloC iM*, and particularly for A5, A6, A11, A12, A17, and A18. These observations suggest interaction with iM loops, which is one of the most common mechanisms of interaction proposed for iM-targeting compounds [24].

### 2.4. Saturation-Transfer Difference (STD) NMR Analysis

In order to confirm the binding of compounds **15c**, **18c**, **20a**, and **23a** to *c-Myc G4* and *hTeloC iM*, a ligand-based NMR approach was used. Particularly, Saturation Transfer Difference NMR (STD NMR) experiments were performed in buffer solutions containing a large excess of compounds (250 μM) with respect to the DNA (20 μM) [53]. STD NMR is based on the magnetization transfer by the Nuclear Overhauser Effect (NOE) from the macromolecule to the ligand. STD NMR spectra are the result of the subtraction of a 1D ^1^H spectrum where the macromolecule protons are selectively saturated (on-resonance spectrum) from a reference spectrum in which the macromolecule is not saturated (off-resonance spectrum). The resulting difference STD spectrum will only show the ligand signals interacting with the macromolecule. The proton resonances for **15c**, **18c**, **20a**, and **23a** were observed in the STD spectra (Figures S10–S13) in presence of both *c-Myc G4* and *hTeloC iM*, demonstrating once again an interaction between the investigated compounds and the DNAs. In the STD spectra, the presence of both aromatic and aliphatic signals for **15c**, **18c**, **20a**, and **23a** suggests that the aromatic moieties of these compounds could interact with the G4 and iM through π-π stacking with the guanines of G-tetrads and the adenines of loops, respectively, while the side chains could interact with the backbone of the DNA molecules through electrostatic interactions. The very low signal intensity of the protons of **21d** in the STD spectra (Figure S14) shows that this compound does not interact with the DNA sequences under investigation.

### 2.5. Evaluation of Ligand Affinity by Fluorescence Experiments

**Figure 5:**
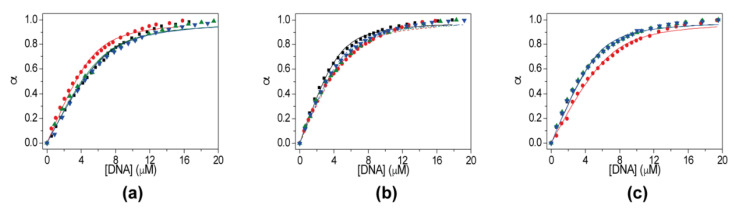
Fluorescence titration curves were obtained by plotting the fraction of bound compound (**20a** in green, **23a** in blue, **15c** in black, and **18c** in red) as a function of (**a**) *c-Myc G4*, (**b**) *c-Myc iM*, and (**c**) *hTeloC iM* concentration. Titrations were carried out in 10 mM potassium phosphate buffer (pH 5.0) for *c-Myc G4*, and 10 mM sodium phosphate buffer (pH 5.0) for *c-Myc iM* and *hTeloC iM*.

To obtain quantitative data regarding the affinity of **15c**, **18c**, **20a**, and **23a** for *c-Myc G4*, *c-Myc iM*, and *hTeloC iM*, fluorescence titration experiments were performed. Fluorescence emission spectra of the compounds in the absence and presence of increasing amounts of oligonucleotides were recorded. The fluorescence intensity of the compounds decreased gradually with the addition of DNA until it reached saturation (Figure S15). The binding curves were obtained by plotting the fraction of bound compound (α), calculated following fluorescence changes at the emission maximum, as a function of DNA concentration (Figure 5). Binding constants (*K*_b_) were obtained from curve fitting using an independent and equivalent binding sites model and reported in Table 2. The results of the interpolation analysis indicated that in those cases the data could not be fitted with a single binding site, but they were well fitted with two binding sites per DNA molecule. Compound **18c**, which displayed the strongest affinity for the G4 structure of *c-Myc*, also exhibited the least affinity for the iM structure of both *c-Myc* and *hTeloC*. On the other hand, compounds **15c**, **20a** and **23a** showed higher affinity values for *c-Myc iM* and *hTeloC iM* rather than to *c-Myc G4.*

Furthermore, fluorescence titration experiments were carried out to investigate the interaction of **21d** with a G4 (*c-Myc G4*) and an iM (*c-Myc iM*) structure (Figure S16a,b). The obtained curves were then fitted giving *K*_b_ values of 1.1 (±0.2) × 10^6^ and 1.6 (±0.2) × 10^6^ M^−1^ for *c-Myc G4* and *c-Myc iM*, respectively, and stoichiometry of 2:1 in both cases. These results confirm the lower (although not null) affinity of this compound for these targets. Finally, the same experiment was performed for **18c** with a C-rich single-stranded oligonucleotide [d(CT)_15_] (Figure S16c), giving a 1:1 stoichiometry and a *K*_b_ of 4.6 (±0.4) × 10^6^ M^−1^, and thus suggesting that the decrease in melting temperature of the iMs could also be due to preferred binding to the unfolded DNA, which would shift the folding equilibrium.

### 2.6. Effect of the Compounds on G4/iM Structures in Living Cells

**Figure 6:**
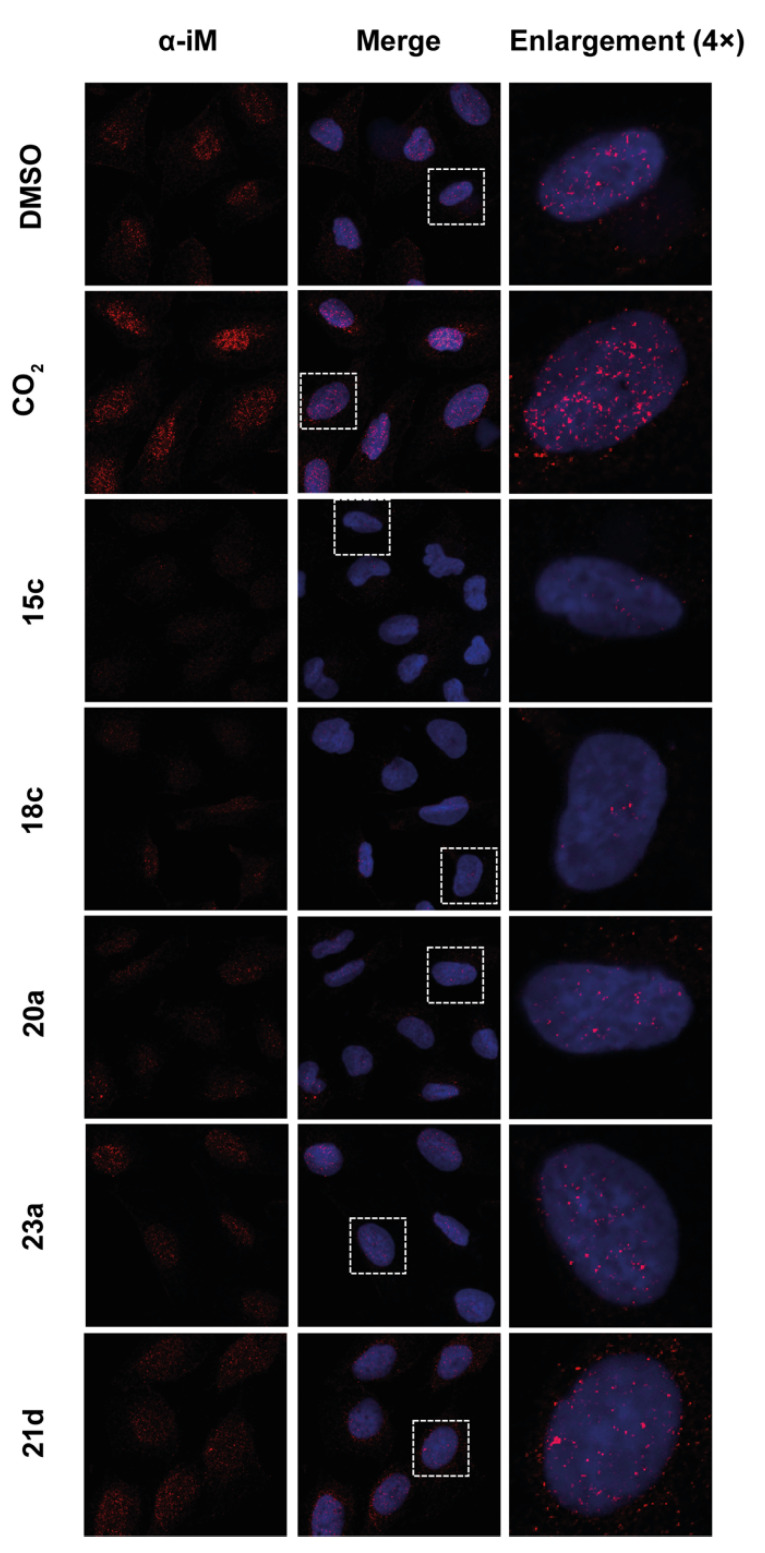
Biological evaluation of the iM-destabilizing activity of the selected compounds. Immunofluorescence analysis of iM structures in U2OS cells treated for 24 h with 2 µM of the selected compounds or an equivalent amount of DMSO (negative control). As a positive control, cells were maintained for 2.5 h in an atmosphere with 8% of CO_2_. Representative images of confocal sections (63×) used to detect iM structures are shown. **Left panels**: iM structures (red) detected by anti-iM antibody (α-iM). **Middle panels**: merged images showing iM structures (red) and DAPI counterstained nuclei (blue). **Right panels**: 4× enlargements from the pictures in the middle panels. The experiment was performed in triplicate and at least 9 fields/experiments were evaluated for each condition. Scale bars are reported in the figures.

**Figure 7:**
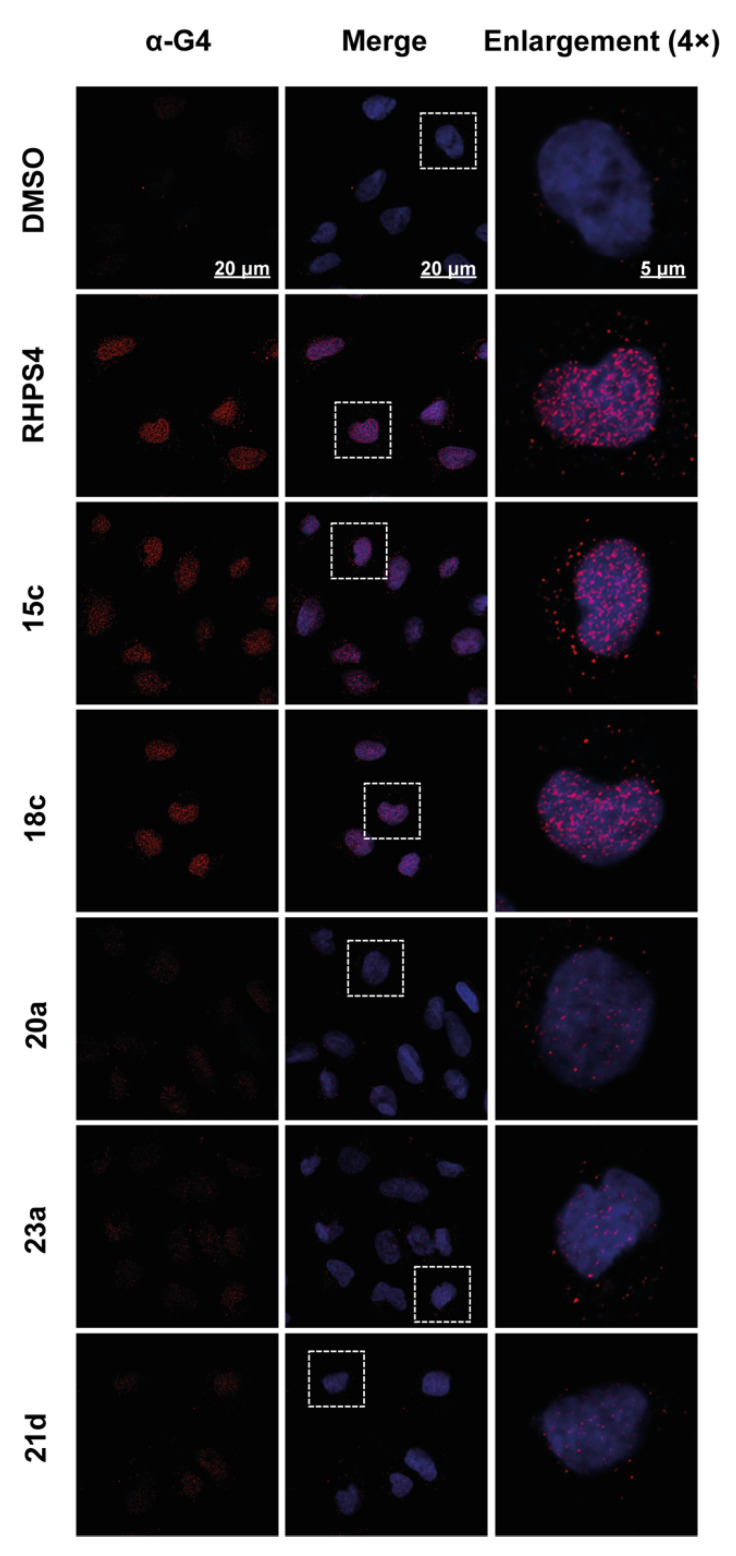
Biological evaluation of G4-stabilizing activity of the selected compounds. Immunofluorescence analysis of G4 structures in U2OS cells treated for 24 h with 2 µM of the selected compounds or an equivalent amount of DMSO (negative control). As a positive control, cells were treated for 24 h with 1 µM RHPS4. Representative images of confocal sections (63×) used for the detection of G4 structures are shown. **Left panels**: G4 structures (red) detected by anti-G4 antibody (α-G4). **Middle panels**: merged images showing G4 structures (red) and DAPI counterstained nuclei (blue). **Right panels**: 4× enlargements from the pictures in the middle panels. The experiment was performed in triplicate and at least 9 fields/experiments were evaluated for each condition. Scale bars are reported in the figures.

Starting from the results of the biophysical assays, the selected molecules (**15c**, **18c**, **20a**, **23a**, and **21d** as a control) were tested for their capability to destabilize the iM and/or stabilize the G4 structures also in the cells. To this aim, U2OS human osteosarcoma cells, one of the models already used by Zeraati and colleagues to validate their anti-iM antibody (iMab) [16], were treated for 24 h with 2 µM of each compound and the iM structures were evaluated by immunofluorescence (IF) microscopy. As a positive control, cells were maintained in 8% of CO_2_ for 2.5 h, a condition that is known to favor the formation of intracellular iM structures [16]. In accordance with the previously reported results, all the investigated molecules, except for **21d**, succeeded in destabilizing the iMs as demonstrated by the significant reduction in the iM structures following the treatments (Figure 6 and Figure S17a). IF experiments highlighted that the compounds differ from each other in terms of potency in destabilizing the iMs, with **15c** and **18c** that—inducing a reduction of about 70% and 60% in the iMs fluorescence signal, respectively—turned out to be the most effective compounds. Notably, the major efficacy of **15c** and **18c** in destabilizing iMs well correlates with their capability to stabilize G4 structures (Figure 7 and Figure S17b). Indeed, the dual activity of **15c** and **18c**, already observed at the biophysical level, is in line with the literature data showing that, under certain conditions, the formation of G4s and iM structures can be reciprocally influenced [55]. Altogether, our data confirmed the ability of the selected compounds to affect G4/iM structures also in the complexity of a cellular model, demonstrating the biological translatability of the biophysical results.

## 3. Materials and Methods

### 3.1. Materials

Commercially available reagents and solvents were purchased from Merck (Darmstadt, Germany) or Alfa Aesar (Karlsruhe, Germany) and used without any further purification. Immediately before using it, tetrahydrofuran (THF) was distilled from Na/benzophenone, under a slight positive atmosphere of dry nitrogen. Distillation from P_2_O_5_ was used to dry dichloromethane, which was then stored on activated molecular sieves (4 Å). When needed, the reactions were carried out in oven-dried glassware, under a positive pressure of dry nitrogen. The determination of the melting points (which are uncorrected) was done in open glass capillaries, with a Stuart scientific SMP3. All the compounds were characterized by employing FT-IR Nicolet Avatar (Thermo Fisher Scientific, Waltham, MA, USA), ^1^H-NMR, ^13^C-NMR (APT) (JEOL ECP 300 MHz, Tokyo, Japan), and mass spectrometry (Thermo Finnigan LCQ-deca XP-plus, Waltham, MA, USA), equipped with an ESI source and an ion trap detector. Chemical shifts (*δ*) are reported in parts per million (ppm). Flash column chromatography was performed on Kieselgel 60, 230–400 mesh ASTM silica gel (Merck, Kenilworth, NJ, USA). Thin-layer chromatography was carried out on 5 × 10 cm plates with a layer thickness of 0.25 mm silica gel 60 F_254_ (Merck, Kenilworth, NJ, USA). When necessary, they were developed with KMnO4 reagent. Elemental analysis (C, H, N) of the tested compounds was used to establish their purity which turned out to be ≥95%, being within ±0.4% of the calculated values. Alkynes **11a**, **11b**, **11c**, **11d**, **12a** were commercially available.

### 3.2. Chemistry

#### 3.2.1. Synthesis of Diethyl 2,6-Dimethyl-4-Phenyl-1,4-Dihydropyridine-3,5-Dicarboxylate (**1**)

To a stirred solution of benzaldehyde (5.0 g, 47.17 mmol, 1 equiv) in water (50 mL), were added ethyl acetoacetate (13.21 mL, 103.77 mmol, 2.2 equiv) and ammonium carbonate (6.79 g, 70.7 mmol, 1.5 equiv). The mixture was heated at 60 °C for 4 h. After the reaction completion, cold water was added, and the product was extracted with dichloromethane (×2). The combined organic layers were washed with brine (×1), dried over anhydrous sodium sulfate, and concentrated *in vacuo*, to give 14.44 g of product as a pale-yellow solid, which was used in the next step without further purification. Yield 93%; mp 160–161 °C (lett. 156–157 °C) [56]; IR (KBr) 3341, 2981, 2934, 2902, 1687, 1650, 1488, 1372, 1211, 1091, 703 cm^−1^; ^1^H-NMR (300 MHz, CDCl_3_) *δ* 7.28–7.10 (m, 5-H), 5.87 (br s, 1-H), 4.98 (s, 1-H), 4.08–4.05 (m, 4-H), 2.30 (s, 6-H), 1.22–1.18 (m, 6-H) ppm; ^13^C-NMR (75 MHz, CDCl_3_) *δ* 167.8, 147.9, 144.1, 128.1, 127.9, 126.1, 104.1, 59.8, 39.7, 19.5, 14.3 ppm.

#### 3.2.2. Synthesis of Diethyl 2,6-Dimethyl-4-Phenylpyridine-3,5-Dicarboxylate (**2**)

To a stirred and cooled (0 °C) solution of 1,4-dihydropyridine **1** (8.07 g, 24.6 mmol, 1 equiv) in glacial acetic acid (16.14 mL, 295.2 mmol, 12 equiv), NaNO_2_ (8.48 g, 123 mmol, 5 equiv) was added in small portions. The resulting mixture was stirred for 2 h at 25 °C. After completion of the reaction, water was added, and the mixture was basified by the addition of sat. aq. NaHCO_3_ solution dropwise. The product was extracted with dichloromethane (×2). The combined organic layers were dried over anhydrous sodium sulfate and concentrated *in vacuo*. Finally, the crude material was purified by column chromatography using PE/EtOAc 8:2 as eluant to give 5 g of a pale-yellow solid; yield 63%; mp 60–62 °C (lett. 63–64.5 °C) [57]; IR (KBr) 2980, 2933, 1728, 1555, 1289, 1228, 1098, 1041, 860, 704 cm^−1^; ^1^H-NMR (300 MHz, CDCl_3_) *δ* 7.28–7.15 (m, 5-H), 3.90 (qd, *J* = 7.0/2.7 Hz, 4-H), 2.52 (s, 6-H), 0.80 (td, *J* = 7.0/2.7 Hz, 6-H) ppm; ^13^C-NMR (75 MHz, CDCl_3_) *δ* 167.9, 155.4, 146.1, 136.6, 128.4, 128.14, 128.11, 126.9, 61.3, 22.9, 13.5 ppm.

#### 3.2.3. Synthesis of (2,6-Dimethyl-4-Phenylpyridine-3,5-Diyl)Dimethanol (**3**)

Under a nitrogen atmosphere LiAlH_4_ (2.71 g, 71.40 mmol, 3 equiv) was added portion-wise to a stirred solution of the corresponding diethylester **2** (7.75 g, 23.8 mmol, 1 equiv) in THF dry (155.0 mL) at 0 °C. The resulting mixture was stirred for 3 h at 25 °C. Then the reaction was quenched by dropwise addition of NaOH 2 M solution under stirring and a white precipitate was formed. The suspension was filtered through a pad of Celite, and the filter cake was washed with MeOH. The filtrate obtained was concentrated *in vacuo* and the residue was purified by column chromatography using EtOAc/MeOH 9:1 as eluant to give 5 g of product as a white solid (lett. pale yellow oil) [58]; yield 79%; mp 164–166 °C; IR (KBr) 3300–2800 br, 1571, 1560, 1443, 1410, 1009, 711 cm^−1^; ^1^H-NMR (300 MHz, CDCl_3_) *δ* 7.45–7.44 (m, 3-H), 7.27–7.25 (m, 2-H), 4.30 (s, 4-H), 2.65 (s, 6-H) ppm; ^13^C-NMR (75 MHz, CDCl_3_) *δ* 158.2, 153.2, 138.3, 131.0, 130.2, 129.1, 128.9, 59.5, 21.6 ppm.

#### 3.2.4. Synthesis of 3,5-Bis(Azidomethyl)-2,6-Dimethyl-4-Phenylpyridine (**4**)

Under a nitrogen atmosphere, DPPA (diphenylphosphoryl azide) (6.64 mL, 30.86 mmol, 3 equiv) and DBU (1,8-Diazabicycloundec-7-ene) (4.60 mL, 30.86 mmol, 3 equiv) were subsequently added dropwise to a cooled (0 °C) and stirred solution of alcohol **3** (2.5 g, 10.28 mmol, 1 equiv) in 75.0 mL di *N,N*′-dimethylformamide dry. Once the additions were completed, the ice bath was removed and allowed to react at RT for 30 min, then it was heated to 80 °C for 1 h. After cooling to RT, the reaction was worked up by dilution with water and extraction with EtOAc (×2). The combined organic layers were washed with water (×3), dried over anhydrous sodium sulfate, and concentrated *in vacuo.* The residue was purified by column chromatography using PE/EtOAc 98:2 and PE/EtOAc 9:1 as eluants to give 2.74 g of product as a white solid; yield 90%, mp 74–76 °C; IR (KBr) 2959, 2932, 2104, 1554, 1488, 1458, 1260, 1193, 960, 770 cm^−1^; ^1^H-NMR (300 MHz, CDCl_3_) *δ* 7.49–7.46 (m, 3-H), 7.15–7.13 (m, 2-H), 4.07 (br s, 4-H), 2.64 (br s, 6-H) ppm; ^13^C-NMR (75 MHz, CDCl_3_) *δ* 157.8, 151.8, 135.8, 129.0, 128.8, 128.5, 125.0, 49.1, 22.6 ppm; ESI-MS *m*/*z* 294 [M + H]^+^.

#### 3.2.5. General Procedure for the Preparation of Bis-Triazolyl-Pyridines **15a**–**24a**, **15b**–**21b**, **15c**–**21c**, **15d**–**21d**

3,5-bis(azidomethyl)-2,6-dimethyl-4-phenylpyridine **4** (0.13 g, 0.44 mmol, 1 equiv) and the corresponding alkyne (0.968, 2.2 equiv) were suspended in a mixture of water/*tert*-butanol (1:1) (4 mL), sodium ascorbate of a freshly prepared 1 M solution in water (0.09 mL, 0.097 mmol, 0.22 equiv) was added, followed by the addition of copper (II) sulfate pentahydrate (0.002 g, 0.0097 mmol, 0.022 equiv). The resulting mixture was vigorously stirred at 25 °C for 3 h under nitrogen. Then the mixture was concentrated *in vacuo* and the crude was purified by column chromatography to give the desired products.

##### Series a: Bis-Triazolyl-Pyridines **15a**–**24a**

1,1′-((((2,6-dimethyl-4-phenylpyridine-3,5-diyl)bis(methylene))bis(1H-1,2,3-triazole-1,4-diyl))bis(methylene))dipiperidine (**15a**)

The title compound was prepared from azide **4** and 1-(prop-2-yn-1-yl)piperidine (**5a**) according to the general procedure. The crude was purified by column chromatography using EtOAc/MeOH 8:2 and EtOAc/MeOH 8:2 + 1% conc. NH_4_OH as eluants to give a white solid: yield 51%; mp 218–220 °C dec; IR (KBr) 3062, 2933, 2851, 2778, 1563, 1465, 1438, 1318, 1299, 1111, 1051, 703 cm^−1^; ^1^H-NMR (300 MHz, CD_3_OD) *δ* 7.44–7.37 (m, 5-H), 7.01 (d, *J =* 7.0 Hz, 2-H), 5.34 (s, 4-H), 3.57 (s, 4-H), 2.59 (s, 6-H), 2.41 (br s, 8-H), 1.58 (br s, 8-H), 1.46(br s, 4-H) ppm; ^13^C-NMR (75 MHz, CD_3_OD) *δ* 159.7, 154.4, 143.8, 136.8, 130.0, 129.9, 129.3, 126.2, 125.4, 54.8, 54.0, 49.7, 26.3, 24.8, 22.4 ppm; ESI-MS *m*/*z* 540 [M + H]^+^. Anal. Calcd for C_31_H_41_N_9_: C, 68.99; H, 7.66; N, 23.36; found: C, 69.14; H, 7.57; N, 23.49. The ^1^H- and ^13^C-NMR (APT) spectra of **15a** are shown in Figure S18.

2,6-dimethyl-4-phenyl-3,5-bis((4-(pyrrolidin-1-ylmethyl)-1H-1,2,3-triazol-1-yl)methyl)pyridine (**16a**)

The title compound was prepared from azide **4** and 1-(prop-2-yn-1-yl)pyrrolidine (**6a**) according to the general procedure. The crude was purified by column chromatography using EtOAc/MeOH 8:2 and EtOAc/MeOH 7:3 + 1% conc. NH_4_OH as eluants to give a white solid: yield 67%; mp 197–199 °C; yield 67%; IR (KBr) 3061, 2961, 2787, 1558, 1444, 1347, 1145, 1053, 702 cm^−1^; ^1^H-NMR (300 MHz, CD_3_OD) *δ* 7.50–7.37 (m, 5-H), 7.07 (d, *J =* 7.3 Hz, 2-H), 5.35 (s, 4-H), 3.70 (s, 4-H), 2.58–2.54 (m, 14-H), 1.79 (br s, 8-H) ppm; ^13^C-NMR (75 MHz, CD_3_OD) *δ* 159.6, 154.3, 145.2, 136.8, 130.0, 129.8, 129.3, 126.2, 124.8, 54.5, 50.6, 49.7, 24.2, 22.4 ppm; ESI-MS *m*/*z* 512 [M + H]^+.^. Anal. Calcd for: C_29_H_37_N_9_: C, 68.07; H, 7.29; N, 24.64; found: C, 68.29; H, 7.42; N, 24.79. The ^1^H- and ^13^C-NMR (APT) spectra of **16a** are shown in Figure S19.

4,4′-((((2,6-dimethyl-4-phenylpyridine-3,5-diyl)bis(methylene))bis(1H-1,2,3-triazole-1,4-diyl))bis(methylene))bis(1-methylpiperazine) (**17a**)

The title compound was prepared from azide **4** and 1-methyl-4-(prop-2-yn-1-yl)piperazine (**7a**) according to the general procedure. The crude was purified by column chromatography using EtOAc/MeOH 3:7 and EtOAc/MeOH 3:7 + 1% conc. NH_4_OH as eluants to give a white solid: yield 80%; mp 221–223 °C dec; IR (KBr) 3101, 3060, 2933, 2797, 2741, 1557, 1455, 1280, 1163, 1139, 1031, 822, 705 cm^−1^; ^1^H-NMR (300 MHz, CD_3_OD) *δ* 7.48–7.37 (m, 5-H), 7.06–7.04 (m, 2-H), 5.35 (s, 4-H), 3.60 (s, 4-H), 2.59 (s, 6-H), 2.50 (br s, 16-H), 2.28 (s, 6-H) ppm; ^13^C-NMR (75 MHz, CD_3_OD) *δ* 159.6, 154.3, 144.1, 136.7, 130.0, 129.8, 129.3, 126.1, 125.2, 55.4, 53.2, 53.0, 49.6, 45.9, 22.4 ppm; ESI-MS *m*/*z* 570 [M + H]^+^. Anal. Calcd for: C_31_H_43_N_11_: C, 65.35; H, 7.61; N, 27.04; found: C, 65.12; H, 7.78; N, 27.19. The ^1^H- and ^13^C-NMR (APT) spectra of **17a** are shown in Figure S20.

4,4′-((((2,6-dimethyl-4-phenylpyridine-3,5-diyl)bis(methylene))bis(1H-1,2,3-triazole-1,4-diyl))bis(methylene))bis(1-isopropylpiperazine) (**18a**)

The title compound was prepared from azide **4** and 1-isopropyl-4-(prop-2-yn-1-yl)piperazine (**8a**) according to the general procedure. The crude was purified by column chromatography using EtOAc/MeOH 7:3 and EtOAc/MeOH 7:3 + 1% conc. NH_4_OH as eluants to give an off-white solid: yield 67%; mp 214–216 °C dec; IR (KBr) 3101, 2964, 2879, 2802, 1566, 1447, 1316, 1177, 1133, 1048, 842, 705 cm^−1^; ^1^H-NMR (300 MHz, CD_3_OD) *δ* 7.47–7.40 (m, 5-H), 7.04 (d, *J =* 7.0 Hz, 2-H), 5.34 (s, 4-H), 3.60 (s, 4-H), 2.72–2.53 (m, 24-H), 1.08 (br d, 12-H) ppm; ^13^C-NMR (75 MHz, CD_3_OD) *δ* 159.7, 154.4, 144.1, 136.8, 130.1, 129.9, 129.4, 126.2, 125.4, 56.0, 53.3, 49.7, 49.3, 22.4, 18.6 ppm; ESI-MS *m*/*z* 626 [M + H]^+^. Anal. Calcd for: C_35_H_51_N_11_: C, 67.17; H, 8.21; N, 24.62; found: C, 67.38; H, 8.35; N, 24.48. The ^1^H- and ^13^C-NMR (APT) spectra of **18a** are shown in Figure S21.

4,4′-((((2,6-dimethyl-4-phenylpyridine-3,5-diyl)bis(methylene))bis(1H-1,2,3-triazole-1,4-diyl))bis(methylene))dimorpholine (**19a**)

The title compound was prepared from azide **4** and 4-(prop-2-yn-1-yl)morpholine (**9a**) according to the general procedure. The crude was purified by column chromatography using EtOAc/MeOH 7:3 and EtOAc/MeOH 7:3 + 1% conc. NH_4_OH as eluants to give a white solid: 78% yield; mp 239–241 °C dec.; IR (KBr) 3063, 2926, 2856, 2813, 1558, 1453, 1328, 1115, 1003, 863, 705 cm^−1^; ^1^H-NMR (300 MHz, CD_3_OD) *δ* 7.44–7.35 (m, 5-H), 7.00 (d, *J =* 6.7 Hz, 2-H), 5.34 (br s, 4-H), 3.66 (br s, 8-H), 3.59 (br s, 4-H), 2.59 (br s, 6-H), 2.43 (br s, 8-H) ppm; ^13^C-NMR (75 MHz, CD_3_OD) *δ* 159.7, 154.4, 144.0, 136.7, 130.0, 129.8, 129.3, 126.2, 125.3, 67.6, 54.1, 53.8, 49.6, 22.3 ppm; ESI-MS *m*/*z* 544 [M + H]^+^. Anal. Calcd for: C_29_H_37_N_9_O_2_: C, 64.07; H, 6.86; N, 23.19; found: C, 63.89; H, 7.03; N, 23.48. The ^1^H- and ^13^C-NMR (APT) spectra of **19a** are shown in Figure S22.

1,1′-((((2,6-dimethyl-4-phenylpyridine-3,5-diyl)bis(methylene))bis(1H-1,2,3-triazole-1,4-diyl))bis(methylene))bis(piperidin-4-ol) (**20a**)

The title compound was prepared from azide **4** and 1-(prop-2-yn-1-yl)piperidin-4-ol (**10a**) according to the general procedure. The crude was purified by column chromatography using EtOAc/MeOH 7:3 and EtOAc/MeOH 7:3 + 1% conc. NH_4_OH as eluents to give a white solid: yield 67%; mp 90–92 °C; IR (KBr) 3400–2700 br, 3139, 2939, 2824, 1557, 1442, 1331, 1223, 1137, 1056, 707 cm^−1^; ^1^H-NMR (300 MHz, CD_3_OD) *δ* 7.45–7.35 (m, 5-H), 7.00 (d, *J =* 6.7 Hz, 2-H), 5.34 (s, 4-H), 3.60 (br s, 6-H), 2.75 (m, 4-H), 2.60 (s, 6-H), 2.20 (m, 4-H), 1.84 (m, 4-H), 1.54 (m, 4-H) ppm; ^13^C-NMR (75 MHz, CD_3_OD) *δ* 159.7, 154.4, 144.2, 136.8, 130.0, 129.9, 129.3, 126.2, 125.3, 67.8, 53.3, 51.6, 49.7, 34.6, 22.4 ppm; ESI-MS *m*/*z* 572 [M + H]^+^. Anal. Calcd for: C_31_H_41_N_9_O_2_: C, 65.13; H, 7.23; N, 22.05; found: C, 64.89; H, 7.36; N, 22.17. The ^1^H- and ^13^C-NMR (APT) spectra of **20a** are shown in Figure S23.

(((2,6-dimethyl-4-phenylpyridine-3,5-diyl)bis(methylene))bis(1H-1,2,3-triazole-1,4-diyl))dimethanol (**21a**)

The title compound was prepared from azide **4** and propargyl alcohol (**11a**) according to the general procedure. The crude was purified by column chromatography using EtOAc/MeOH 9:1 and EtOAc/MeOH 8:2 as eluants to give an amorphous yellow solid: yield 83%; IR (KBr) 3400–2800 br, 1557, 1445, 1218, 1142, 1037 cm^−1^; ^1^H-NMR (300 MHz, CD_3_OD) *δ* 7.45–7.39 (m, 5-H), 7.04 (d, *J =* 7.3 Hz, 2-H), 5.33 (s, 4-H), 4.59 (s, 4-H), 2.56 (s, 6-H) ppm; ^13^C-NMR (75 MHz, CD_3_OD) *δ* 159.7, 154.4, 148.9, 136.7, 130.0, 129.9, 129.3, 126.2, 125.0, 56.3, 49.7, 22.3 ppm; ESI-MS *m*/*z* 406 [M + H]^+^. Anal. Calcd for: C_21_H_23_N_7_O_2_: C, 62.21; H, 5.72; N, 24.18; found: 62.03; H, 5.89; N, 24.43. The ^1^H- and ^13^C-NMR (APT) spectra of **21a** are shown in Figure S24.

1,1′-(((2,6-dimethyl-4-phenylpyridine-3,5-diyl)bis(methylene))bis(1H-1,2,3-triazole-1,4-diyl))bis(N,N-dimethylmethanamine) (**22a**)

The title compound was prepared from azide **4** and 1-dimethylamino-2-propyne (**12a**) according to the general procedure. The crude was purified by column chromatography using EtOAc/MeOH 8:2 and EtOAc/MeOH 8:2 + 1% conc. NH_4_OH as eluants to give a white solid: yield 48%; mp 184–186 °C; IR (KBr) 3110, 3069, 2941, 2814, 2763, 1561, 1456, 1142, 1052, 1036, 841, 704 cm^−1^; ^1^H-NMR (300 MHz, CD_3_OD) *δ* 7.51–7.05 (m, 5-H), 7.06 (d, *J =* 6.7 Hz, 2-H), 5.36 (s, 4-H), 3.56 (s, 4-H), 2.58 (s, 6-H), 2.22 (br s, 12-H) ppm; ^13^C-NMR (75 MHz, CD_3_OD) *δ* 159.6, 154.3, 144.3, 136.7, 130.0, 129.8, 129.3, 126.1, 125.2, 54.1, 49.7, 44.8, 22.4 ppm; ESI-MS *m*/*z* 460 [M + H]^+^. Anal. Calcd for: C_25_H_33_N_9_: C, 65.33; H, 7.24; N, 27.43; found: C, 65.59; H, 7.39; N, 27.22. The ^1^H- and ^13^C-NMR (APT) spectra of **22a** are shown in Figure S25.

2,2′,2″,2‴-(((((2,6-dimethyl-4-phenylpyridine-3,5-diyl)bis(methylene))bis(1H-1,2,3-triazole-1,4-diyl))bis(methylene))bis(azanetriyl))tetrakis(ethan-1-ol) (**23a**)

The title compound was prepared from azide **4** and 2,2′-(prop-2-yn-1-ylazanediyl)bis(ethan-1-ol) (**13a**) according to the general procedure. The crude was purified by column chromatography using EtOAc/MeOH 7:3 and EtOAc/MeOH 7:3 + 1% conc. NH_4_OH as eluants to give a yellow oil: yield 66%; IR (KBr) 3500–2800 br, 1576, 1443, 1336, 1219, 1141, 1044, 764, 708 cm^−1^; ^1^H-NMR (300 MHz, CD_3_OD) *δ* 7.48 (s, 2-H), 7.41–7.39 (m, 3-H), 7.01 (d, *J =* 6.7 Hz, 2-H), 5.33 (br s, 4-H), 3.78 (br s, 4-H), 3.61–3.58 (m, 8-H), 2.59 (br s, 14-H) ppm; ^13^C-NMR (75 MHz, CD_3_OD) *δ* 159.7, 154.5, 145.5, 136.7, 130.1, 129.9, 129.3, 126.2, 125.0, 60.4, 57.1, 49.8, 49.7, 22.3 ppm; ESI-MS *m*/*z* 602 [M + Na]^+^. Anal. Calcd for: C_29_H_41_N_9_O_4_: C, 60.09; H, 7.13; N, 21.75; found: C, 60.32; H, 6.95; N, 21.58. The ^1^H- and ^13^C-NMR (APT) spectra of **23a** are shown in Figure S26.

4,4′-((((2,6-dimethyl-4-phenylpyridine-3,5-diyl)bis(methylene))bis(1H-1,2,3-triazole-1,4-diyl))bis(methylene))bis(1-(methylsulfonyl)piperazine) (**24a**)

The title compound was prepared from azide **4** and 1-(methylsulfonyl)-4-(prop-2-yn-1-yl)piperazine (**14a**) according to the general procedure. The crude was purified by column chromatography using EtOAc/MeOH 7:3 and EtOAc/MeOH 7:3 + 1% conc. NH_4_OH as eluants to give a light orange oil: yield 67%; mp 244–246 °C dec.; IR (KBr) 3067, 2925, 2850, 2822,1561, 1455, 1342, 1323, 1156, 1133, 960, 785 cm^−1^; ^1^H-NMR (300 MHz, CD_3_OD) *δ* 7.37–7.35 (m, 3-H), 6.99–6.94 (m, 4-H), 5.21 (br s, 4-H), 3.61 (br s, 4-H), 3.19 (br s, 8-H), 2.74 (br s, 6-H), 2.55 (br s, 14-H) ppm; ^13^C-NMR (75 MHz, CD_3_OD) *δ* 158.8, 152.2, 143.4, 135.4, 129.2, 129.1, 128.1, 124.0, 122.5, 52.7, 52.0, 48.8, 45.7, 34.4, 22.9 ppm; ESI-MS *m*/*z* 698 [M + H]^+^. Anal. Calcd for: C_31_H_43_N_11_O_4_S_2_: C, 53.35; H, 6.21; N, 22.08; found: C, 53.54; H, 5.98; N, 22.23. The ^1^H- and ^13^C-NMR (APT) spectra of **24a** are shown in Figure S27.

##### Series b: Bis-Triazolyl-Pyridines **15b**–**21b**

1,1′-((((2,6-dimethyl-4-phenylpyridine-3,5-diyl)bis(methylene))bis(1H-1,2,3-triazole-1,4-diyl))bis(ethane-2,1-diyl))dipiperidine (**15b**)

The title compound was prepared from azide **4** and 1-(but-3-yn-1-yl)piperidine (**5b**) according to the general procedure. The crude was purified by column chromatography using EtOAc/MeOH 8:2 and EtOAc/MeOH 8:2 + 1% conc. NH_4_OH as eluants to give a white solid: yield 92%; mp 214–216 °C dec.; IR (KBr) 3110, 3058, 2933, 2804, 2769, 1560, 1438, 1143, 1055, 1030, 703 cm^−1^; ^1^H-NMR (300 MHz, CD_3_OD) *δ* 7.43–7.30 (m, 5-H), 6.99 (d, *J =* 6.7 Hz, 2-H), 5.30 (s, 4-H), 2.82 (t, *J =* 7.6 Hz, 4-H), 2.60–2.48 (m, 18-H), 1.60 (br s, 8-H), 1.48 (br s, 4-H) ppm; ^13^C-NMR (75 MHz, CD_3_OD) *δ* 159.6, 154.3, 146.7, 136.8, 130.0, 129.8, 129.3, 126.3, 123.6, 59.6, 55.2, 26.5, 25.1, 23.5, 22.3 ppm; ESI-MS *m*/*z* 568 [M + H]^+^. Anal. Calcd for: C_33_H_45_N_9_: C, 69.81; H, 7.99; N, 22.20; found: C, 69.69; H, 8.21; N, 21.97. The ^1^H- and ^13^C-NMR (APT) spectra of **15b** are shown in Figure S28.

2,6-dimethyl-4-phenyl-3,5-bis((4-(2-(pyrrolidin-1-yl)ethyl)-1H-1,2,3-triazol-1-yl)methyl)pyridine (**16b**)

The title compound was prepared from azide **4** and 1-(but-3-yn-1-yl)pyrrolidine (**6b**) according to the general procedure. The crude was purified by column chromatography using EtOAc/MeOH 7:3 and EtOAc/MeOH 7:3 + 1% conc. NH_4_OH as eluants to give an off-white solid: yield 69%; mp 182–184 °C; IR (KBr) 3112, 3062, 2955, 2787, 1562, 1442, 1146, 1052, 1033, 841, 702 cm^−1^; ^1^H-NMR (300 MHz, CD_3_OD) *δ* 7.41–7.31 (m, 5-H), 7.01 (d, *J =* 6.7 Hz, 2-H), 5.30 (s, 4-H), 2.87–2.82 (m, 4-H), 2.76–2.71 (m, 4-H), 2.61–2.57 (m, 14-H), 1.82 (br s, 8-H) ppm; ^13^C-NMR (75 MHz, CD_3_OD) *δ* 159.6, 154.4, 146.5, 136.8, 130.0, 129.8, 129.3, 126.3, 123.6, 56.7, 54.9, 25.6, 24.2, 22.3 ppm; ESI-MS *m*/*z* 540 [M + H]^+^. Anal. Calcd for: C_31_H_41_N_9_: C, 68.99; H, 7.66; N, 23.36; found: C, 69.25; H, 7.82; N, 23.17. The ^1^H- and ^13^C-NMR (APT) spectra of **16b** are shown in Figure S29.

4,4′-((((2,6-dimethyl-4-phenylpyridine-3,5-diyl)bis(methylene))bis(1H-1,2,3-triazole-1,4-diyl))bis(ethane-2,1-diyl))bis(1-methylpiperazine) (**17b**)

The title compound was prepared from azide **4** and 1-(but-3-yn-1-yl)-4-methylpiperazine (**7b**) according to the general procedure. The crude was purified by column chromatography using EtOAc/MeOH 3:7 and EtOAc/MeOH 3:7 + 1% conc. NH_4_OH as eluants to give an off-white solid: yield 81%; mp 149–152 °C dec.; IR (KBr) 2937, 2810, 2690, 1556, 1455, 1284, 1164, 1146, 1055, 1009, 839 cm^−1^; ^1^H-NMR (300 MHz, CD_3_OD) *δ* 7.42–7.35 (m, 5-H), 7.02 (d, *J =* 6.4 Hz, 2-H), 5.31 (s, 4-H), 2.82 (t, *J =* 7.4 Hz, 4-H) 2.63–2.56 (m, 26-H), 2.30 (s, 6-H) ppm; ^13^C-NMR (75 MHz, CD_3_OD) *δ* 159.5, 154.2, 146.6, 136.8, 130.0, 129.8, 129.3, 126.3, 123.7, 58.5, 55.6, 53.3, 49.5, 45.9, 23.7, 22.4 ppm; ESI-MS *m*/*z* 598 [M + H]^+^. Anal. Calcd for: C_33_H_47_N_11_: C, 66.30; H, 7.92; N, 25.77; found: C, 66.12; H, 8.16; N, 26.09. The ^1^H- and ^13^C-NMR (APT) spectra of **17b** are shown in Figure S30.

4,4′-((((2,6-dimethyl-4-phenylpyridine-3,5-diyl)bis(methylene))bis(1H-1,2,3-triazole-1,4-diyl))bis(ethane-2,1-diyl))bis(1-isopropylpiperazine) (**18b**)

The title compound was prepared from azide **4** and 1-(but-3-yn-1-yl)-4-isopropylpiperazine (**8b**) according to the general procedure. The crude was purified by column chromatography using EtOAc/MeOH 7:3 and EtOAc/MeOH 7:3 + 1% conc. NH_4_OH as eluants to give a white solid: yield 82%; mp 224–226 °C dec.; IR (KBr) 3113, 3058, 2960, 2938, 2813, 1561, 1464, 1448, 1271, 1178, 1147, 1055, 1030, cm^−1^; ^1^H-NMR (300 MHz, CD_3_OD) *δ* 7.41–7.34 (m, 5-H), 7.01 (d, *J =* 6.7 Hz, 2-H), 5.30 (s, 4-H), 2.82 (t, *J =* 6.7 Hz, 4-H) 2.69–2.56 (m, 28-H), 1.07 (br d, 12-H) ppm; ^13^C-NMR (75 MHz, CD_3_OD) *δ* 159.5, 154.3, 146.6, 136.8, 130.0, 129.8, 129.3, 126.3, 123.6, 58.6, 55.9, 53.7, 49.5, 49.4, 23.6, 22.3, 18.7 ppm; ESI-MS *m*/*z* 654 [M + H]^+^. Anal. Calcd for: C_37_H_55_N_11_: C, 67.96; H, 8.48; N, 23.56; found: C, 67.84; H, 8.65; N, 23.31. The ^1^H- and ^13^C-NMR (APT) spectra of **18b** are shown in Figure S31.

4,4′-((1,1′-((2,6-dimethyl-4-phenylpyridine-3,5-diyl)bis(methylene))bis(1H-1,2,3-triazole-4,1-diyl))bis(ethane-2,1-diyl))dimorpholine (**19b**)

The title compound was prepared from azide **4** and 4-(but-3-yn-1-yl)morpholine (**9b**) according to the general procedure. The crude was purified by column chromatography using EtOAc/MeOH 8:2 as eluant to give a white solid: yield 46%; mp 200–204 °C dec.; IR (KBr) 3116, 3064, 2942, 2852, 2812, 1564, 1461, 1446, 1274, 1212, 1115, 1006, 869, 703 cm^−1^; ^1^H-NMR (300 MHz, CD_3_OD) *δ* 7.43–7.38 (m, 3-H), 7.29 (s, 2-H), 7.00 (d, *J* = 6.7 Hz, 2-H), 5.30 (s, 4-H), 3.66 (br s, 8-H), 2.82 (t, *J* = 7.4 Hz, 4-H), 2.61–2.57 (m, 12-H), 2.48 (br s, 6-H) ppm; ^13^C-NMR (75 MHz, CD_3_OD) *δ* 159.6, 154.3, 146.6, 136.8, 130.0, 129.9, 129.3, 126.3, 123.7, 67.7, 59.1, 54.5, 49.6, 23.4, 22.4 ppm; ESI-MS *m*/*z* 572 [M + H]^+^. Anal. Calcd for: C_31_H_41_N_9_O_2_: C, 65.13; H, 7.23; N, 22.05; found: C, 65.29; H, 7.48; N, 21.86. The ^1^H- and ^13^C-NMR (APT) spectra of **19b** are shown in Figure S32.

1,1′-((1,1′-((2,6-dimethyl-4-phenylpyridine-3,5-diyl)bis(methylene))bis(1H-1,2,3-triazole-4,1-diyl))bis(ethane-2,1-diyl))bis(piperidin-4-ol) (**20b**)

The title compound was prepared from azide **4** and 1-(but-3-yn-1-yl)piperidin-4-ol (**10b**) according to the general procedure. The crude was purified by column chromatography using EtOAc/MeOH 7:3 + 1% conc. NH_4_OH as eluant to give a white solid: yield 77%; mp 58–62 °C; IR (KBr) 3135, 2939, 2813, 1554, 1444, 1340, 1218, 1136, 1072, 762, 707, cm^−1^; ^1^H-NMR (300 MHz, CD_3_OD) *δ* 7.41–7.38 (m, 3-H), 7.33 (s, 2-H), 7.00 (d, *J* = 6.7 Hz, 2-H), 5.31 (s, 4-H), 3.64 (br s, 2-H), 2.85–2.80 (m, 8-H), 2.63–2.57 (m, 10-H), 2.24 (br t, 4-H), 1.87 (br d, 4-H), 1.61–1.55 (m, 4-H) ppm; ^13^C-NMR (75 MHz, CD_3_OD) *δ* 159.6, 154.3, 146.6, 136.8, 130.0, 129.9, 129.3, 126.3, 123.7, 68.0, 58.7, 52.0, 49.6, 34.7, 23.8, 22.4 ppm; ESI-MS *m*/*z* 600 [M + H]^+^. Anal. Calcd for: C_33_H_45_N_9_O_2_: C, 66.08; H, 7.56; N, 21.02; found: C, 65.84; H, 7.73; N, 21.28. The ^1^H- and ^13^C-NMR (APT) spectra of **20b** are shown in Figure S33.

2,2′-(((2,6-dimethyl-4-phenylpyridine-3,5-diyl)bis(methylene))bis(1H-1,2,3-triazole-1,4-diyl))bis(ethan-1-ol) (**21b**)

The title compound was prepared from azide **4** and 3-butyn-1-ol (**11b**) according to the general procedure. The crude was purified by column chromatography using EtOAc/MeOH 9:1 and EtOAc/MeOH 8:2 eluants to give an amorphous off-white solid: yield 88%; IR (KBr) 2800–3200 br, 1556, 1444, 1218, 1131, 1053, 707cm^−1^; ^1^H-NMR (300 MHz, CD_3_OD) *δ* 7.40–7.29 (m, 5-H), 7.01 (d, *J =* 7.0 Hz, 2-H), 5.30 (s, 4-H), 3.73; (td, *J =* 6.7/1.8 Hz, 4-H), 2.81(td, *J =* 6.7/1.8 Hz, 4-H), 2.57 (s, 6-H) ppm; ^13^C-NMR (75 MHz, CD_3_OD) *δ* 159.6, 154.4, 146.0, 136.7, 130.0, 129.8, 129.3, 126.3, 123.9, 61.9, 49.5, 29.7, 22.3 ppm; ESI-MS *m*/*z* 434 [M + H]^+^. Anal. Calcd for: C_23_H_27_N_7_O_2_: C, 63.72; H, 6.28; N, 22.62; found: C, 63.44; H, 6.42; N, 22.32. The ^1^H- and ^13^C-NMR (APT) spectra of **21b** are shown in Figure S34.

##### Series c: Bis-Triazolyl-Pyridines **15c**–**21c**

1,1′-((((2,6-dimethyl-4-phenylpyridine-3,5-diyl)bis(methylene))bis(1H-1,2,3-triazole-1,4-diyl))bis(propane-3,1-diyl))dipiperidine (**15c**)

The title compound was prepared from azide **4** and 1-(pent-4-yn-1-yl)piperidine (**5c**) according to the general procedure. The crude was purified by column chromatography using EtOAc/MeOH 8:2 and EtOAc/MeOH 8:2 + 1% conc. NH_4_OH as eluants to give a white solid: yield 79%; mp 159–161 °C; IR (KBr) 3058, 2931, 2850, 2800, 2762, 1562, 1441, 1150, 1110, 1053, 1037, 702 cm^−1^; ^1^H-NMR (300 MHz, CD_3_OD) *δ* 7.43–7.29 (m, 5-H), 7.02 (d, *J =* 7.0 Hz, 2-H), 5.30 (s, 4-H), 2.62 (t, *J =* 7.0 Hz, 4-H), 2.56 (br s, 6-H), 2.43 (br s, 8-H), 2.35 (d, *J =* 7.2 Hz, 4-H), 1.81 (quint, *J =* 7.0 Hz, 4-H), 1.59 (br s, 8-H), 1.47 (br s, 4-H) ppm; ^13^C-NMR (75 MHz, CD_3_OD) *δ* 159.5, 154.3, 148.4, 136.8, 130.0, 129.8, 129.3, 126.2, 123.2, 59.5, 55.4, 49.5, 27.1, 26.4, 25.1, 24.2, 22.3 ppm; ESI-MS *m*/*z* 596 [M + H]^+^. Anal. Calcd for: C_35_H_49_N_9_: C, 70.55; H, 8.29; N, 21.16; found: C, 70.32; H, 8.53; N, 21.35. The ^1^H- and ^13^C-NMR (APT) spectra of **15c** are shown in Figure S35.

2,6-dimethyl-4-phenyl-3,5-bis((4-(3-(pyrrolidin-1-yl)propyl)-1H-1,2,3-triazol-1-yl)methyl)pyridine (**16c**)

The title compound was prepared from azide **4** and 1-(pent-4-yn-1-yl)pyrrolidine (**6c**) according to the general procedure. The crude was purified by column chromatography using EtOAc/MeOH 6:4 and EtOAc/MeOH 6:4 + 1% conc. NH_4_OH as eluants to give a dark-beige solid: yield 47%; mp 130–133 °C; IR (KBr) 3107, 3056, 2941, 2775, 1557, 1444, 1144, 1052, 1029, 840, 702 cm^−1^; ^1^H-NMR (300 MHz, CD_3_OD) *δ* 7.44–7.31 (m, 5-H), 7.02 (d, *J =* 6.5 Hz, 2-H), 5.31 (s, 4-H), 2.68–2.53 (m, 22-H), 1.89–1.82 (m, 12-H) ppm; ^13^C-NMR (75 MHz, CD_3_OD) *δ* 159.5, 154.2, 148.2, 136.8, 130.0, 129.8, 129.3, 123.3, 56.6, 54.9, 49.5, 29.1, 24.1, 22.3 ppm; ESI-MS *m*/*z* 568 [M + H]^+^. Anal. Calcd for: C_33_H_45_N_9_: C, 69.81; H, 7.99; N, 22.20; found: C, 70.03; H, 8.12; N, 22.47. The ^1^H- and ^13^C-NMR (APT) spectra of **16c** are shown in Figure S36.

4,4′-((((2,6-dimethyl-4-phenylpyridine-3,5-diyl)bis(methylene))bis(1H-1,2,3-triazole-1,4-diyl))bis(propane-3,1-diyl))bis(1-methylpiperazine) (**17c**)

The title compound was prepared from azide **4** and 1-methyl-4-(pent-4-yn-1-yl)piperazine (**7c**) according to the general procedure. The crude was purified by column chromatography using EtOAc/MeOH 3:7 and EtOAc/MeOH 3:7 + 1% conc. NH_4_OH as eluants to give a white solid: yield 72%; mp 139–141 °C; IR (KBr) 3109, 3057, 2937, 2794, 1558, 1457, 1296, 1161, 1051, 814, 705 cm^−1^; ^1^H-NMR (300 MHz, CD_3_OD) *δ* 7.41–7.30 (m, 5-H), 7.02 (d, *J =* 6.4 Hz, 2-H), 5.30 (s, 4-H), 2.64; (t, *J =* 7.3 Hz, 4-H), 2.56 (br s, 6-H), 2.50 (br s, 8-H), 2.37 (t, *J =* 7.0 Hz, 4-H), 2.28 (br s, 6-H), 1.80 (quint, *J =* 7.3 Hz, 4-H) ppm; ^13^C-NMR (75 MHz, CD_3_OD) *δ* 159.5, 154.2, 148.4, 136.8, 130.0, 129.8, 129.3, 126.2, 123.2, 58.6, 55.5, 53.6, 49.5, 45.9, 27.2, 24.0, 22.4 ppm; ESI-MS *m*/*z* 626 [M + H]^+^. Anal. Calcd for: C_35_H_51_N_11_: C, 67.17; H, 8.21; N, 24.62; found: C, 66.99; H, 8.37; N, 24.45. The ^1^H- and ^13^C-NMR (APT) spectra of **17c** are shown in Figure S37.

4,4′-((((2,6-dimethyl-4-phenylpyridine-3,5-diyl)bis(methylene))bis(1H-1,2,3-triazole-1,4-diyl))bis(propane-3,1-diyl))bis(1-isopropylpiperazine) (**18c**)

The title compound was prepared from azide **4** and 1-isopropyl-4-(pent-4-yn-1-yl)piperazine (**8c**) according to the general procedure. The crude was purified by column chromatography using EtOAc/MeOH 7:3 and EtOAc/MeOH 7:3 + 1% conc. NH_4_OH as eluants to give a white solid: yield 79%; mp 153–156 °C; IR (KBr) 3115, 3061, 2943, 2804, 2770, 1560, 1442, 1268, 1182, 1053, 984, 703 cm^−1^; ^1^H-NMR (300 MHz, CD_3_OD) *δ* 7.42–7.32 (m, 5-H), 7.06 (d, *J =* 6.1 Hz, 2-H), 5.32 (s, 4-H), 2.67–2.37 (m, 32-H), 1.81 (m, 4-H), 1.09–1.06 (m, 12-H) ppm; ^13^C-NMR (75 MHz, CD_3_OD) *δ* 159.4, 154.0, 148.3, 136.8, 130.0, 129.8, 129.3, 126.2, 123.1, 58.6, 55.8, 53.9, 49.5, 49.4, 27.2, 24.0, 22.5, 18.8 ppm; ESI-MS *m*/*z* 682 [M + H]^+^. Anal. Calcd for: C_39_H_59_N_11_: C, 68.69; H, 8.72; N, 22.59; found: C, 68.94; H, 8.55; N, 22.35. The ^1^H- and ^13^C-NMR (APT) spectra of **18c** are shown in Figure S38.

4,4′-((1,1′-((2,6-dimethyl-4-phenylpyridine-3,5-diyl)bis(methylene))bis(1H-1,2,3-triazole-4,1-diyl))bis(propane-3,1-diyl))dimorpholine (**19c**)

The title compound was prepared from azide **4** and 4-(pent-4-yn-1-yl)morpholine (**9c**) according to the general procedure. The crude was purified by column chromatography using EtOAc/MeOH 9:1 + 1% conc. NH_4_OH as eluant to give a white solid: yield 52%; mp 164–166 °C; IR (KBr) 3114, 3061, 2943, 2849, 2809, 1710, 1562, 1453, 1293, 1137, 1117, 864, 706, cm^−1^; ^1^H-NMR (300 MHz, CD_3_OD) *δ* 7.44–7.39 (m, 3-H), 7.29 (s, 2-H), 7.01 (d, *J* = 6.4 Hz, 2-H), 5.30 (s, 4-H), 3.69–3.66 (m, 8-H), 2.64 (t, *J* = 7.3, 4-H), 2.56 (br s, 6-H), 2.44 (br s, 8-H), 2.35 (t, *J* = 7.3, 4-H), 1.80 (quint, *J* = 7.7 Hz, 4-H) ppm; ^13^C-NMR (75 MHz, CD_3_OD) *δ* 159.6, 154.4, 148.5, 136.9, 130.1, 129.9, 129.3, 126.4, 123.4, 67.6, 59.2, 54.8, 49.6, 27.0, 24.0, 22.4 ppm; ESI-MS *m*/*z* 600 [M + H]^+^. Anal. Calcd for: C_33_H_45_N_9_O_2_: C, 66.08; H, 7.56; N, 21.02; found: C, 65.82; H, 7.76; N, 20.91. The ^1^H- and ^13^C-NMR (APT) spectra of **19c** are shown in Figure S39.

1,1′-((1,1′-((2,6-dimethyl-4-phenylpyridine-3,5-diyl)bis(methylene))bis(1H-1,2,3-triazole-4,1-diyl))bis(propane-3,1-diyl))bis(piperidin-4-ol) (**20c**)

The title compound was prepared from azide **4** and 1-(pent-4-yn-1-yl)piperidin-4-ol (**10c**) according to the general procedure. The crude was purified by column chromatography using EtOAc/MeOH 8:2 + 1% conc. NH_4_OH and EtOAc/MeOH 7:3 + 1% conc. NH_4_OH as eluants to give a pale yellow solid: yield 57%; mp 78–81 °C; IR (KBr) 3300, 3134, 2940, 2812, 1672, 1561, 1444, 1340, 1217, 1135, 1062, 763, 707, cm^−1^; ^1^H-NMR (300 MHz, CD_3_OD) *δ* 7.41–7.39 (m, 3-H), 7.28 (s, 2-H), 7.01 (d, *J* = 7.3 Hz, 2-H), 5.30 (s, 4-H), 3.63 (m, 2-H), 2.82–2.78 (m, 4-H), 2.63 (t, *J* = 7.4 Hz, 4-H), 2.56 (br s, 6-H), 2.36 (t, *J* = 7.1 Hz, 4-H), 2.19 -2.13 (m, 4-H), 1.93–1.78 (m, 8-H), 1.61–1.51 (m, 4-H) ppm; ^13^C-NMR (75 MHz, CD_3_OD) *δ* 159.5, 154.3, 148.4, 136.8, 130.0, 129.8, 129.3, 126.3, 123.3, 68.1, 58.7, 52.2, 49.6, 34.7, 27.4, 24.1, 22.4 ppm; ESI-MS *m*/*z* 628 [M + H]^+^. Anal. Calcd for: C_35_H_49_N_9_O_2_: C, 66.96; H, 7.87; N, 20.08; found: C, 66.83; H, 7.99; N, 20.27. The ^1^H- and ^13^C-NMR (APT) spectra of **20c** are shown in Figure S40.

3,3′-(((2,6-dimethyl-4-phenylpyridine-3,5-diyl)bis(methylene))bis(1H-1,2,3-triazole-1,4-diyl))bis(propan-1-ol) (**21c**)

The title compound was prepared from azide **4** and 4-pentyn-1-ol (**11c**) according to the general procedure. The crude was purified by column chromatography using EtOAc/MeOH 9:1 and EtOAc/MeOH 8:2 as eluants to give an amorphous yellow solid: yield 88%; IR (KBr) 3400–3000 br, 2944, 2870, 1556, 1442, 1216, 1054, 707 cm^−1^; ^1^H-NMR (300 MHz, CD_3_OD) *δ* 7.42–7.35 (m, 3-H), 7.26 (s, 2-H), 6.98 (d, *J =* 7.0 Hz, 2-H), 5.30 (s, 4-H), 3.54 (td, *J =* 6.4/2.1 Hz, 4-H), 2.68 (t, *J =* 6.4 Hz, 4-H), 2.57 (s, 6-H), 1.80 (quint, *J =* 6.4 Hz, 4-H) ppm; ^13^C-NMR (75 MHz, CD_3_OD) *δ* 159.5, 154.3, 148.5, 136.7, 130.0, 129.8, 129.2, 126.3, 123.2, 61.9, 49.5, 33.2, 22.5, 22.3 ppm; ESI-MS *m*/*z* 462 [M + H]^+^. Anal. Calcd for: C_25_H_31_N_7_O_2_: C, 65.06; H, 6.77; N, 21.24; found: C, 65.32; H, 6.93; N, 20.93. The ^1^H- and ^13^C-NMR (APT) spectra of **21c** are shown in Figure S41.

##### Series d: Bis-Triazolyl-Pyridines **15d**–**21d**

1,1′-((1,1′-((2,6-dimethyl-4-phenylpyridine-3,5-diyl)bis(methylene))bis(1H-1,2,3-triazole-4,1-diyl))bis(butane-4,1-diyl))dipiperidine (**15d**)

The title compound was prepared from azide **4** and 1-(hex-5-yn-1-yl)piperidine (**5d**) according to the general procedure. The crude was purified by column chromatography using EtOAc/MeOH 9:1 and EtOAc/MeOH 8:2 + 1% conc. NH_4_OH as eluants to give a pale yellow solid: yield 65%; mp 173–176 °C dec.; IR (KBr) 3116, 3062, 2930, 2856, 2768, 1700, 1561, 1440, 1146, 1053, 702, cm^−1^; ^1^H-NMR (300 MHz, CD_3_OD) *δ* 7.44–7.38 (m, 3-H), 7.28 (s, 2-H), 7.02 (d, *J* = 6.5 Hz, 2-H), 5.30 (s, 4-H), 2.66–2.55 (m, 10-H), 2.42–2.31 (m, 12-H), 1.60–1.48 (m, 20-H) ppm; ^13^C-NMR (75 MHz, CD_3_OD) *δ* 159.6, 154.4, 148.7, 136.9, 130.0, 129.9, 129.4, 126.3, 123.3, 60.1, 55.5, 49.6, 28.6, 26.8, 26.4, 26.0, 25.2, 22.3 ppm; ESI-MS *m*/*z* 624 [M + H]^+^. Anal. Calcd for: C_37_H_53_N_9_: C, 71.23; H, 8.56; N, 20.21; found: C, 71.05; H, 8.79; N, 20.48. The ^1^H- and ^13^C-NMR (APT) spectra of **15d** are shown in Figure S42.

2,6-dimethyl-4-phenyl-3,5-bis((4-(4-(pyrrolidin-1-yl)butyl)-1H-1,2,3-triazol-1-yl)methyl)pyridine (**16d**)

The title compound was prepared from azide **4** and 1-(hex-5-yn-1-yl)pyrrolidine (**6d**) according to the general procedure. The crude was purified by column chromatography using EtOAc/MeOH 8:2 and EtOAc/MeOH 7:3 + 1% conc. NH_4_OH as eluants to give a pale yellow solid: yield 49%; mp 138–141 °C; IR (KBr) 3114, 3062, 2929, 2858, 1618, 1562, 1462, 1219, 1144, 1051, 700, 587, cm^−1^; ^1^H-NMR (300 MHz, CD_3_OD) *δ* 7.44–7.39 (m, 3-H), 7.30 (s, 2-H), 7.03 (d, *J* = 7.1 Hz, 2-H), 5.30 (s, 4-H), 2.67–2.50 (m, 22-H), 1.81 (br s, 8-H), 1.63–1.55 (m, 8-H) ppm; ^13^C-NMR (75 MHz, CD_3_OD) *δ* 159.6, 154.3, 148.7, 136.9, 130.0, 129.9, 129.4, 126.3, 123.3, 57.1, 55.0, 49.6, 28.9, 28.5, 26.0, 24.1, 22.3 ppm; ESI-MS *m*/*z* 596 [M + H]^+^. Anal. Calcd for: C_35_H_49_N_9_: C, 70.55; H, 8.29; N, 21.16; found: C, 70.43; H, 8.15; N, 21.41. The ^1^H- and ^13^C-NMR (APT) spectra of **16d** are shown in Figure S43.

4,4′-((1,1′-((2,6-dimethyl-4-phenylpyridine-3,5-diyl)bis(methylene))bis(1H-1,2,3-triazole-4,1-diyl))bis(butane-4,1-diyl))bis(1-methylpiperazine) (**17d**)

The title compound was prepared from azide **4** and 1-(hex-5-yn-1-yl)-4-methylpiperazine (**7d**) according to the general procedure. The crude was purified by column chromatography using EtOAc/MeOH 8:2 + 1% conc. NH_4_OH as eluant to give a yellow solid: yield 76%; mp 146–149 °C dec; IR (KBr) 3115, 3063, 2934, 2860, 2793, 1618, 1561, 1459, 1446, 1280, 1164, 820, 700, cm^−1^; ^1^H-NMR (300 MHz, CD_3_OD) *δ* 7.41–7.39 (m, 3-H), 7.28 (s, 2-H), 7.01 (d, *J* = 7.3 Hz, 2-H), 5.30 (s, 4-H), 2.64–2.32 (m, 36-H), 1.65–1.52 (m, 8-H) ppm; ^13^C-NMR (75 MHz, CD_3_OD) *δ* 159.6, 154.4, 148.7, 136.9, 130.0, 129.8, 129.3, 126.3, 123.3, 59.0, 55.4, 53.5, 49.6, 45.8, 28.4, 26.8, 25.9, 22.3 ppm; ESI-MS *m*/*z* 654 [M + H]^+^. Anal. Calcd for: C_37_H_55_N_11_: C, 67.96; H, 8.48; N, 23.56; found: C, 67.65; H, 8.66; N, 23.75. The ^1^H- and ^13^C-NMR (APT) spectra of **17d** are shown in Figure S44.

4,4′-((1,1′-((2,6-dimethyl-4-phenylpyridine-3,5-diyl)bis(methylene))bis(1H-1,2,3-triazole-4,1-diyl))bis(butane-4,1-diyl))bis(1-isopropylpiperazine) (**18d**)

The title compound was prepared from azide **4** and 1-(hex-5-yn-1-yl)-4-isopropylpiperazine (**8d**) according to the general procedure. The crude was purified by column chromatography using EtOAc and EtOAc/MeOH 9:1 + 1% conc. NH_4_OH as eluants to give a white solid: yield 61%; mp189–193 °C; IR (KBr) 3116, 3061, 2962, 2931, 2811, 1561, 1467, 1446, 1269, 1183, 1146, 1054, 703, cm^−1^; ^1^H-NMR (300 MHz, CD_3_OD) *δ* 7.42–7.39 (m, 3-H), 7.28 (s, 2-H), 7.02 (d, *J* = 6.8 Hz, 2-H), 5.30 (s, 4-H), 2.66–2.37 (m, 32-H), 1.62–1.51 (m, 8-H), 1.08 (d, *J* = 6.5 Hz, 12-H) ppm; ^13^C-NMR (75 MHz, CD_3_OD) *δ* 159.6, 154.4, 148.7, 136.9, 130.1, 129.9, 129.4, 126.3, 123.3, 59.3, 55.9, 54.0, 49.6, 49.4, 28.5, 26.9, 26.0, 22.4, 18.7 ppm; ESI-MS *m*/*z* 711 [M + H]^+^. Anal. Calcd for: C_41_H_63_N_11_: C, 69.36; H, 8.94; N, 21.70; found: C, 69.55; H, 8.73; N, 21.89. The ^1^H- and ^13^C-NMR (APT) spectra of **18d** are shown in Figure S45.

4,4′-((1,1′-((2,6-dimethyl-4-phenylpyridine-3,5-diyl)bis(methylene))bis(1H-1,2,3-triazole-4,1-diyl))bis(butane-4,1-diyl))dimorpholine (**19d**)

The title compound was prepared from azide **4** and 4-(hex-5-yn-1-yl)morpholine (**9d**) according to the general procedure. The crude was purified by column chromatography using EtOAc/MeOH 9:1 and EtOAc/MeOH 9:1 + 1% conc. NH_4_OH as eluants to give a white solid: yield 62%; mp 152–156 °C; IR (KBr) 3060, 2931, 2855, 2808, 1703, 1560, 1445, 1115, 1053, 1031, 865, 702, 589, cm^−1^; ^1^H-NMR (300 MHz, CD_3_OD) *δ* 7.41–7.39 (m, 3-H), 7.29 (s, 2-H), 7.04 (d, *J* = 5.8 Hz, 2-H), 5.30 (s, 4-H), 3.67–3.66 (m, 8-H), 2.64 (t, *J* = 6.7 Hz, 4-H), 2.55 (br s, 6-H), 2.42–2.33 (m, 12-H), 1.66–1.61 (m, 4-H), 1.54–1.51 (m, 4-H) ppm; ^13^C-NMR (75 MHz, CD_3_OD) *δ* 159.5, 154.2, 148.7, 136.9, 130.0, 129.8, 129.3, 126.3, 123.2, 67.6, 59.7, 54.7, 49.6, 28.3, 26.6, 26.0, 22.4 ppm; ESI-MS *m*/*z* 628 [M + H]^+^. Anal. Calcd for: C_35_H_49_N_9_O_2_: C, 66.96; H, 7.87; N, 20.08; found: C, 67.25; H, 7.99; N, 20.25. The ^1^H- and ^13^C-NMR (APT) spectra of **19d** are shown in Figure S46.

1,1′-((1,1′-((2,6-dimethyl-4-phenylpyridine-3,5-diyl)bis(methylene))bis(1H-1,2,3-triazole-4,1-diyl))bis(butane-4,1-diyl))bis(piperidin-4-ol) (**20d**)

The title compound was prepared from azide **4** and 1-(hex-5-yn-1-yl)piperidin-4-ol (**10d**) according to the general procedure. The crude was purified by column chromatography using EtOAc/MeOH 9:1 + 1% conc. NH_4_OH and EtOAc/MeOH 7:3 + 1% conc. NH_4_OH as eluants to give a white solid: yield 87%; mp 159–162 °C; IR (KBr) 3406, 3058, 2940, 2765, 1557, 1444, 1362, 1130, 1062, 1031, 699, 584, cm^−1^; ^1^H-NMR (300 MHz, CD_3_OD) *δ* 7.44–7.36 (m, 3-H), 7.28 (s, 2-H), 7.02 (d, *J* = 6.4 Hz, 2-H), 5.30 (s, 4-H), 3.64–3.62 (m, 2-H), 2.83–2.79 (m, 4-H), 2.64 (t, *J* = 7.4 Hz, 4-H), 2.56 (br s, 6-H), 2.37 (t, *J* = 7.4 Hz, 4-H), 2.20–2.14 (m, 4-H), 1.88–1.84 (m, 4-H), 1.61–1.51 (m, 12-H) ppm; ^13^C-NMR (75 MHz, CD_3_OD) *δ* 159.6, 154.3, 148.7, 136.9, 130.0, 129.8, 129.3, 126.3, 123.2, 68.0, 59.2, 52.1, 49.6, 34.6, 28.5, 27.0, 25.9, 22.4 ppm; ESI-MS *m*/*z* 656 [M + H]^+^. Anal. Calcd for: C_37_H_53_N_9_O_2_: C, 67.76; H, 8.15; N, 19.22; found: C, 67.89; H, 8.23; N, 18.97. The ^1^H- and ^13^C-NMR (APT) spectra of **20d** are shown in Figure S47.

4,4′-(1,1′-((2,6-dimethyl-4-phenylpyridine-3,5-diyl)bis(methylene))bis(1H-1,2,3-triazole-4,1-diyl))bis(butan-1-ol) (**21d**)

The title compound was prepared from azide **4** and 5-hexyn-1-ol (**11d**) according to the general procedure. The crude was purified by column chromatography using EtOAc/MeOH 9:1 as eluant to give a white solid: yield 95%; mp 105–108 °C; IR (KBr) 3275, 3109, 3055, 2923, 2853, 1554, 1442, 1215, 1146, 1076, 1048, 842, 703, 589, cm^−1^; ^1^H-NMR (300 MHz, CD_3_OD) *δ* 7.40–7.38 (m, 3-H), 7.28 (s, 2-H), 7.01 (d, *J* = 6.4 Hz, 2-H), 5.30 (s, 4-H), 3.56 (t, *J* = 6.4 Hz, 4-H), 2.63 (t, *J* = 7.3 Hz, 4-H), 2.57 (s, 6-H), 1.66 (quint, *J* = 7.4 Hz, 4-H), 1.52 (quint, *J* = 7.4 Hz, 4-H) ppm; ^13^C-NMR (75 MHz, CD_3_OD) *δ* 159.5, 154.3, 148.8, 136.8, 130.0, 129.8, 129.3, 126.3, 123.2, 62.4, 49.6, 33.0, 26.8, 25.9, 22.4 ppm; ESI-MS *m*/*z* 512 [M + Na]^+^. Anal. Calcd for: C_27_H_35_N_7_O_2_: C, 66.23; H, 7.21; N, 20.03; found: C, 66.42; H, 7.01; N, 20.36. The ^1^H- and ^13^C-NMR (APT) spectra of **21d** are shown in Figure S48.

### 3.3. Oligonucleotide Synthesis and Sample Preparation

The following deoxyribonucleotide sequences were selected for the experiments: the promoter sequences of d(GGG CGC GGG AGG AAT TGG GCG GG) (*Bcl-2 G4*), d(TGA GGG TGG GTA GGG TGG GTA A) (*c-Myc G4*), d(TAG GGT TAG GGT TAG GGT TAG GG) (*Tel_23_ G4*), d(CAG CCC CGC TCC CGC CCC CTT CCT CCC GCG CCC GCC CCT) (*Bcl-2 iM*), d(TTC CCC ACC CTC CCT ACC CTA A) (*c-Myc iM*), d(CCC TAA CCC TAA CCC TAA CCC T) (*hTeloC iM*), d(CGC GAA TTC GCG TTT CGC GAA TTC GCG) (*Hairpin*). The oligonucleotides were chemically synthesized at a 5-μmol scale on an ABI 394 DNA/RNA synthesizer (Applied Biosystem, Foster City, CA, USA), by using the standard β-cyanoethylphosphoramidite solid-phase chemistry, as described elsewhere [59]. The subsequent DNA detachment from support and removal of the semi-permanent protection groups were achieved upon treatment with an aqueous solution of concentrated ammonia at 55 °C, for 12 h. After being combined and concentrated under reduced pressure, the filtrates and the washings were solubilized in water and then purified by high-performance liquid chromatography (HPLC) equipped with a Nucleogel SAX 1000-8/46 column (Macherey-Nagel, GmbH & Co. KG, Düren, Germany). Different buffers were used for the purification step: buffer A, consisting of a 20 mM MH_2_PO_4_/M_2_HPO_4_ aqueous solution (pH 7.0) (where M stands for K^+^ or Na^+^ ion), and buffer B, consisting of 1.0 M MCl, 20 mM MH_2_PO_4_/M_2_HPO_4_ aqueous solution (pH 7.0). Both buffer A and B also contained 20% (*v*/*v*) CH_3_CN. A 30 min linear gradient going from 0 to 100% buffer B with a flow rate of 1 mL/min was employed. Desalting of the purified fractions was obtained by means of Sep-pak cartridges (C-18). The purity of the isolated oligomers was checked by NMR and proved to be higher than 98%. The oligonucleotide concentrations were established by measuring the UV absorption at 90 °C, taking into account the appropriate molar extinction coefficient values ε (λ = 260 nm) calculated by the nearest neighbor model [60]. The DNA samples were then dissolved in the proper buffer, as described below. Finally, to achieve the correct folding of the DNA sequences, the oligonucleotide solutions were heated in a water bath at 90 °C, for 5 min, and then left to cool down slowly, at RT, overnight. 

### 3.4. Circular Dichroism (CD) Experiments

The samples for CD measurements were prepared by dissolving *Bcl-2 G4*, *c-Myc G4,* and *Tel_23_ G4* in 25 mM potassium phosphate buffer (pH 7.0), and *Bcl-2 iM*, *c-Myc iM*, and *hTeloC iM* in 10 mM sodium phosphate buffer (pH 5.0) [61], and the *Hairpin* in 10 mM sodium phosphate buffer (pH 7.0). Circular dichroism (CD) experiments (spectra and melting) were performed on a Jasco J-815 spectropolarimeter (JASCO Inc., Tokyo, Japan) equipped with a PTC-423S/15 Peltier temperature controller, using a quartz cuvette with a path length of 0.1 cm. Data was obtained from samples at 15–20 μM final oligonucleotide concentration, in the absence and presence of 10 molar equivalents of each compound (2.5, 5, or 10 mM in 100% DMSO). CD spectra were recorded at 5 and 90 °C for *Bcl-2 iM*, *c-Myc iM*, and *hTeloC iM*, and at 20 °C and 100 °C for *Bcl-2 G4*, *c-Myc G4*, *Tel_23_ G4*, and *Hairpin*. Each spectrum was acquired in a wavelength range of 220–360 nm, averaged over three scans, and subtracted from the buffer baseline. The scanning speed was set to 100 nm/min, with a 4 s response time, 1 nm data pitch, and 2 nm bandwidth. CD melting experiments were performed at 1 °C/min heating rate, in the 5–90 °C temperature range for *Bcl-2 iM*, *c-Myc iM*, and *hTeloC iM*, and in the 20–100 °C temperature range for *Bcl-2 G4*, *c-Myc G4*, *Tel_23_ G4,* and *Hairpin*. Changes in the CD signal were followed at the wavelengths of the maximum CD intensity: 264 nm for *Bcl-2 G4* and *c-Myc G4*, 287 nm for *Tel_23_ G4*, 288 nm for *Bcl-2 iM*, *c-Myc iM,* and *hTeloC iM*. As for *Hairpin*, CD melting curves were recorded by following the change in the CD signal at 251 nm, the wavelength of the minimum intensity value of the respective CD spectrum. All CD melting curves were normalized between 0 and 1. The apparent melting temperatures (*T*_1/2_) were mathematically calculated by using the curve fitting function in Origin 7.0 software (OriginLab, Northampton, MA, USA). The *T*_1/2_ values of the DNAs alone are: *Bcl-2 G4* = 59.1 (±0.9) °C; *c-Myc G4* = 78.6 (±0.4) °C; *Tel_23_ G4* = 55 (±0.7) °C; *Bcl-2 iM* = 62.8 (±0.9) °C; *c-Myc iM* = 73.6 (±0.8) °C; *hTeloC iM* = 51.5 (±0.2) °C; *Hairpin* = 71.1 (±0.9) °C. Δ*T*_1/2_ values correspond to the difference between the DNA melting temperatures with and without compounds.

### 3.5. Principal Component Analysis

The Δ*T*_1/2_ values obtained from the CD experiments were collected in a data matrix consisting of 31 rows (compounds) and 7 columns (DNA sequences) as reported in Table 1. Before PCA, data was converted to absolute values, then mean-centered and scaled to unit variance (autoscaling). Autoscaling employs the standard deviation as a scaling factor thus giving all the Δ*T*_1/2_ values the same chance to affect the model. Moreover, data relative to the *Hairpin* and *Tel_23_ G4* sequences were removed before the analysis because of the very small variation of the Δ*T*_1/2_ values (<4 °C). PCA was then computed by using PLS Toolbox 8.6.1 (Eigenvector Research Inc., Wenatchee, WA, USA) in Matlab R2015b (The Mathworks Inc., Natick, MA, USA) environment. The pK_a_ values for each compound were calculated by using the Percepta Software version 14.3.0 (ACD/Labs, Toronto, ON, Canada).

### 3.6. Nuclear Magnetic Resonance Experiments

#### 3.6.1. 1D ^1^H-NMR Experiments

The samples for nuclear magnetic resonance (NMR) spectroscopy were prepared at 100–150 μM final oligonucleotide concentration, in 250 μL (H_2_O/D_2_O 9:1) buffer solution (10 mM sodium phosphate buffer (pH 5.0) for *hTeloC iM*, and 10 mM potassium phosphate buffer (pH 5.0) for *c-Myc G4*). DNA/compound mixtures were obtained by adding 2 equivalents of the compounds directly to the DNA solution inside the NMR tube [59], with a final DMSO concentration of 6%. NMR experiments were performed by employing a 700 MHz Bruker spectrometer. In particular, 1D ^1^H-NMR spectra were recorded at 10 °C with a ‘zgesgp’ pulse-program (a gradient-based excitation sculpting using 180° water-selective pulses), including the following parameters: 128 scans, spectral width 17,241 Hz, delay 3 s, receiver gain 101, and 32 k points. All NMR spectra were calibrated by centering the water signal at 4.69 ppm (at 10 °C and pH 5.0). NMR data processing was done by using the vendor software TOPSPIN 4.0.7 (Bruker Biospin Gmbh, Rheinstetten, Germany).

#### 3.6.2. STD NMR Experiments

In the 1D ^1^H ligand-based STD NMR experiments, each compound (250 μM), previously solubilized in DMSO-d_6_, was added to a DNA solution (20 μM, 600 μL) in 100% of deuterium oxide containing 10 mM phosphate buffer, pH 5.0. All the spectra were acquired at 298 K with a Bruker AVANCE NEO NMR spectrometer (Bruker Biospin Gmbh, Rheinstetten, Germany) operating at 700 MHz (^1^H Larmor frequency), equipped with a 5 mm TCI 3 channels HCN cryo-probe head, optimized for ^1^H sensitivity. The spectrometer was also equipped with a SampleCase (autosampler) for NMR screening. The spectra were processed with the vendor software TOPSPIN 4.0.7 (Bruker Biospin Gmbh, Rheinstetten, Germany). Saturation Transfer Difference spectra were acquired with 1024 scans, with on-resonance irradiation at 5.95 and 9.12 ppm for selective saturation of *c-Myc G4* and *hTeloC iM* resonances, respectively, and off-resonance irradiation at 40 ppm for reference spectra. A train of 40 Gaussian-shaped pulses of 50 ms (with 1 ms delay between pulses) was used, for a total saturation time of 4 s. The saturation width of the used radiofrequency pulses was 100 Hz. The STD spectra were obtained by internal subtraction of the saturated spectrum from the reference spectrum by phase cycling, with a spectral width of 20 ppm, relaxation delay 1.0 s, 16 k data points for acquisition, and 65 k for transformation.

### 3.7. Fluorescence Titrations

Fluorescence titration experiments were performed at 25 °C on an FP-8300 spectrofluorometer (Jasco, Easton, MD, USA) equipped with a Peltier temperature controller system (Jasco PCT-818, Jasco, Easton, MD, USA). A 1 cm path length, sealed quartz cuvette was used. Titrations were carried out by stepwise addition (5 μL) of *c-Myc G4*, *c-Myc iM*, *hTeloC iM*, or a single-stranded DNA [d(CT)_15_] solution (150–200 μM) to a cell containing a fixed concentration of a compound solution (2.5–3.5 μM) in the appropriate buffer (10 mM potassium phosphate buffer (pH 5.0) for *c-Myc G4*, and 10 mM sodium phosphate buffer (pH 5.0) for *c-Myc iM*, *hTeloC iM*, and single-stranded DNA). The excitation wavelength was set at 275 nm, and emission spectra were recorded in the wavelength range of 285–600 nm. Both excitation and emission slit widths were set at 5 nm. After each DNA addition, the solution was stirred and allowed to equilibrate for 5 min before spectrum acquisition. The fraction of bound compound (α) at each point of the titration was calculated following the changes of fluorescence intensity at the maximum of intensity (307 nm). The compound concentration was corrected for dilution effects resulting from the change in volume due to DNA solution addition. It should be pointed out that the excitation of compounds at 275 nm led to a certain filter effect due to DNA absorption at this wavelength, which in part contributes to the observed quenching of compound fluorescence independent of binding. Titration curves were obtained by plotting α versus the DNA concentration. The equilibrium dissociation constant (*K*_b_) and the stoichiometry of interaction were estimated by fitting the resulting curve to an independent and equivalent binding site model as previously described [62]. The experiments were repeated in duplicate, and the results are presented as the mean ± S.D.

### 3.8. Biological Experiments

#### 3.8.1. Cell Culture and Treatments

Human osteosarcoma cells (U2OS) were purchased from American Type Culture Collection (ATCC) and were grown in Dulbecco’s Modified Eagle Medium (DMEM, Euroclone, Milan, Italy) supplemented with 10% Fetal Bovine Serum (FBS), 2 mM L-glutamine, and antibiotics, at 37 °C, in a 5% CO_2_-95% air atmosphere. As a positive control for the stabilization of iM structures, U2OS were cultured in the presence of 8% of CO_2_ for 2.5 h, as reported elsewhere [16]. As for G-quadruplex structures stabilization, treatment with 1 µM RHPS4 for 24 h was used as a positive control [63]. All compounds were dissolved at a concentration of 10 mM in DMSO. Cells were incubated for 24 h with the compounds, at a final concentration of 2 µM.

#### 3.8.2. Immunofluorescence (IF) Microscopy Experiments

For the IF experiments, cells were fixed in 4% formaldehyde in phosphate-buffered saline (PBS), at RT, for 10 min, permeabilized in 0.25% Triton X-100 in PBS 1×, at RT, for 5 min, and incubated with blocking solution (3% FBS in PBS 1×) at RT for 1 h. For immuno-labeling, cells were incubated at RT, for 2 h, with the flag-tagged antibody that recognizes iM structures. Successively, cells were washed three times with PBS 1× and incubated with the antibody anti-Flag (Sigma-Aldrich, St. Louis, MO, USA, #F7425) for 1 h. Finally, after three washes in PBS 1×, cells were incubated with the antibody anti-rabbit IgG (H + L), F(ab′)2 Fragment (Alexa Fluor 555 Conjugate) (Cell Signaling, Danvers, MA, USA, #4413S) for 1 h. For G4 structures staining, after the permeabilization, cells were incubated with the specific antibody (Anti-DNA/RNA G-quadruplex (BG4), Absolute Antibody, Oxford, UK, #Ab00174-1.1) at RT, for 2 h, washed three times with PBS 1×, and incubated with the antibody anti-mouse IgG (H + L), F(ab’)2 Fragment (Alexa Fluor 555 Conjugate) (Cell Signaling Technology, Danvers, MA, USA, #4409S) for 1 h. For all the IF experiments, nuclei were stained with 4′,6-diamidino-2-phenylindole (DAPI, Sigma-Aldrich, St. Louis, MO, USA, #D9542), and fluorescence signals were recorded by using a Zeiss LSM 880 with airyscan (Zeiss, Jena, Germany), 63× magnification. IF experiments were quantified by Image J (version 1.53e, National Institutes of Health, NIH, Bethesda, MD, USA). 25 cells were screened for each condition and the results were expressed as fold change of fluorescence intensity (anti-G4 or anti-iM signal) over the negative control (DMSO). Histograms show the mean ± SD of three independent experiments. Statistical significance was calculated using unpaired student *t*-tests on Prism 6 (GraphPad, San Diego, CA, USA).

## 4. Conclusions

In the present study, we rationally designed a new library of potential G4/iM-targeting compounds according to the main structural features required to design effective noncanonical DNA-interacting compounds. To this aim, we took advantage of a novel synergistic approach by combining the Hantzsch multicomponent reaction and the CuAAC click chemistry reaction to generate a library of 31 bis-triazolyl-pyridine derivatives. Then, using multiple biophysical techniques, we dissected the profile of each compound in terms of effects on G4/iM structures. Of note, these experiments were corroborated by the application of the PCA method to select the most promising compounds. Finally, in order to translate the biophysical data into a more complex environment, we probed the capability of the selected compounds to affect the formation of G4/iM structures in U2OS cells.

Interestingly, our biophysical screening led to the identification of compounds **15c** and **18c** as candidates capable of concomitantly stabilizing the G4 structures and destabilizing the iM ones, and compounds **20a** and **23a** that selectively affected the thermal stability of the iMs, showing negligible effects on G4 structures. NMR and fluorescence titration experiments provided insights into the interaction mode and affinity of the selected compounds towards the noncanonical DNA structures under investigation. Noteworthy, the results of the biophysical study found confirmation also at the biological level. Indeed, analyses performed by confocal microscopy showed that the same compounds were able to destabilize iM structures in U2OS cells, the best-characterized cell model used so far to study iMs formation. Intriguingly, **15c** and **18c** were able to effectively destabilize iM structures by simultaneously stabilizing G4s, so resulting—also in the complexity of the cellular environment—the most effective compounds among those synthesized and tested.

How G4 and iM structures reciprocally influence each other, and which biological implications are regulated by such a delicate balance are still open questions under intense investigation. Thus, the need for new experimental approaches able to disentangle this scientific dilemma is urgent. In this scenario, our investigation led to the identification of new compounds that might be used as tools to shed light on the mechanisms underlying the controversial biological roles of G4 and iM structures and their intricated relationship.

## Figures and Tables

**Table 1:** Compound-induced thermal stabilization or destabilization of the investigated oligonucleotides, measured by circular dichroism melting experiments. Δ*T*_1/2_ values are the differences in the apparent melting temperatures of DNA in the presence and absence of compounds.

Compound	Δ*T*_1/2_ (°C) ^1^						
	*Hairpin*	*Bcl-2 G4*	*c-Myc G4*	*Tel_23_ G4*	*Bcl-2 iM*	*c-Myc iM*	*hTeloC iM*
**15a**	0	5.0	7.0	1.1	−7.0	−18.4	−12
**16a**	−0.2	4.5	5.0	1.5	−11.0	−27.5	−25.0
**17a**	−1.0	4.0	5.0	0	−10.5	−27.0	−24.5
**18a**	0.2	4.5	7.5	3.5	−13.5	−24.5	−21.0
**19a**	1.7	0.5	2.0	1.0	−4.0	−5.5	−4.0
**20a**	3.9	0.5	1.0	0	−8.0	−10.4	−18.0
**21a**	2.9	0	0	0	0	−0.8	2.5
**22a**	−0.2	2.7	4.5	0.5	−11.0	−19.0	−25.5
**23a**	2.9	0	0.5	0.5	−6.0	−17.0	−12.5
**24a**	−0.7	1.5	2.0	−0.5	−2.0	−2.8	1.5
**15b**	−0.1	5.1	7.5	1.6	−6.0	−20	−26.5
**16b**	−0.1	4.5	8.0	1.6	−13.0	−19.4	−24.5
**17b**	−0.1	4.0	5.5	1.0	−10	−17.4	−23.5
**18b**	−0.1	5.5	8.5	1.5	−6.0	−11.4	−15.5
**19b**	0	3.0	2.0	1.2	−5.0	−4.0	−7.0
**20b**	2.2	5.9	4.0	0.1	−8.0	−6.0	−9.0
**21b**	1.9	1.0	1.0	2.0	0	−0.4	2.0
**15c**	−0.1	6.1	10.6	0	−11.0	−24	−29
**16c**	−0.1	5.6	9.5	0.5	−10.0	−17.4	−24.1
**17c**	−0.1	5.6	6.5	2.0	−12.0	−21.1	−27.4
**18c**	1.1	7.6	10.5	1.6	−10.5	−20.0	−25.5
**19c**	−1.1	5.1	4.2	−1.0	−3.0	−3.0	−10.0
**20c**	3.0	7.1	6.8	0	−9.0	−2.9	−9.0
**21c**	1.9	0	0	2.0	0	−0.9	1.5
**15d**	0	8.8	9.8	−1.0	−5.0	−4.9	−7.0
**16d**	−0.3	8.0	8.1	2.0	−5.0	−4.9	−7.0
**17d**	1.0	9.0	6.9	2.0	−8.0	−7.0	−8.9
**18d**	0	10.1	9.8	−1.0	−8.0	−8.0	−9.0
**19d**	0.9	4.2	2.8	−2.0	−5.0	−5.0	−7.0
**20d**	2.9	8.0	8.2	0	−8.0	−5.0	−9.0
**21d**	1.9	2.0	0.8	−0.8	−1.0	0	−1.0

^1^ The error on Δ*T*_1/2_ values is ±1.0 °C. All experiments were performed in duplicate, and the values reported are the average of two measurements.

**Table 2:** Binding constant (*K*_b_) values were obtained by fitting the fluorescence titration curves to an independent and equivalent binding sites model [54].

Compound	*K*_b_ (×10^6^ M^−1^) ^1^		
	*c-Myc G4*	*c-Myc iM*	*hTeloC iM*
**20a**	2.3	3.1	3.1
**23a**	2.2	3.2	3.1
**15c**	2.5	3.4	3.2
**18c**	3.1	2.8	2.6

^1^ The error on the *K*_b_ values is ±0.2.

## Data Availability

Not applicable.

## Supplementary Materials

The Supplementary Materials can be found at https://www.mdpi.com/article/10.3390/ijms222111959/s1.

## Abbreviations

APT	Attached proton test
ATCC	American type culture collection
BCL-2	B-cell lymphoma 2
CD	Circular dichroism
c-MYC	Cellular MYC
CuAAC	Copper(I)-catalyzed alkyne-azide cycloaddition
DAPI	4′,6-diamidino-2-phenylindole
DBU	1,8-diazabicycloundec-7-ene
DMEM	Dulbecco’s modified eagle medium
DMF	*N,N*′-dimethylformamide
DMSO	Dimethyl sulfoxide
DPPA	Diphenylphosphoryl azide
ESI	Electrospray ionization
FBS	Fetal bovine serum
FT-IR	Fourier transform infrared
G4	G-quadruplex
HPLC	High-performance liquid chromatography
IF	Immunofluorescence
iM	i-Motif
MS	Mass spectrometry
NMR	Nuclear magnetic resonance
NOE	Nuclear Overhauser effect
PBS	Phosphate buffered saline
PCA	Principal component analysis
PE	Petroleum ether
RT	Room temperature
STD	Saturation transfer difference
THF	Tetrahydrofuran

## References

[1] Bacolla A., Wells R.D. (2009). Non-B DNA Conformations as Determinants of Mutagenesis and Human Disease. Mol. Carcinog..

[2] Abou Assi H., Garavís M., González C., Damha M.J. (2018). I-Motif DNA: Structural Features and Significance to Cell Biology. Nucleic Acids Res..

[3] Gajarský M., Živković M.L., Stadlbauer P., Pagano B., Fiala R., Amato J., Tomáška L., Šponer J., Plavec J., Trantírek L. (2017). Structure of a Stable G-Hairpin. J. Am. Chem. Soc..

[4] Spiegel J., Adhikari S., Balasubramanian S. (2020). The Structure and Function of DNA G-Quadruplexes. Trends Chem..

[5] Lane A.N., Chaires J.B., Gray R.D., Trent J.O. (2008). Stability and Kinetics of G-Quadruplex Structures. Nucleic Acids Res..

[6] Bhattacharyya D., Mirihana Arachchilage G., Basu S. (2016). Metal Cations in G-Quadruplex Folding and Stability. Front. Chem..

[7] Largy E., Mergny J.-L., Gabelica V., Sigel A., Sigel H., Sigel R. (2016). Role of Alkali Metal Ions in G-Quadruplex Nucleic Acid Structure and Stability. The Alkali Metal Ions: Their Role for Life. Metal Ions in Life Sciences.

[8] Pagano B., Mattia C.A., Cavallo L., Uesugi S., Giancola C., Fraternali F. (2008). Stability and Cations Coordination of DNA and RNA 14-Mer G-Quadruplexes: A Multiscale Computational Approach. J. Phys. Chem. B.

[9] Guédin A., Gros J., Alberti P., Mergny J.-L. (2010). How Long Is Too Long? Effects of Loop Size on G-Quadruplex Stability. Nucleic Acids Res..

[10] Bugaut A., Balasubramanian S. (2008). A Sequence-Independent Study of the Influence of Short Loop Lengths on the Stability and Topology of Intramolecular DNA G-Quadruplexes. Biochemistry.

[11] Marsico G., Chambers V.S., Sahakyan A.B., McCauley P., Boutell J.M., Di Antonio M., Balasubramanian S. (2019). Whole Genome Experimental Maps of DNA G-Quadruplexes in Multiple Species. Nucleic Acids Res..

[12] Zizza P., Cingolani C., Artuso S., Salvati E., Rizzo A., D’Angelo C., Porru M., Pagano B., Amato J., Randazzo A. (2016). Intragenic G-Quadruplex Structure Formed in the Human CD133 and Its Biological and Translational Relevance. Nucleic Acids Res..

[13] Kim N. (2019). The Interplay between G-Quadruplex and Transcription. Curr. Med. Chem..

[14] Brown S.L., Kendrick S. (2021). The I-Motif as a Molecular Target: More Than a Complementary DNA Secondary Structure. Pharmaceuticals.

[15] Dzatko S., Krafcikova M., Hänsel-Hertsch R., Fessl T., Fiala R., Loja T., Krafcik D., Mergny J.-L., Foldynova-Trantirkova S., Trantirek L. (2018). Evaluation of the Stability of DNA I-Motifs in the Nuclei of Living Mammalian Cells. Angew. Chem. Int. Ed..

[16] Zeraati M., Langley D.B., Schofield P., Moye A.L., Rouet R., Hughes W.E., Bryan T.M., Dinger M.E., Christ D. (2018). I-Motif DNA Structures Are Formed in the Nuclei of Human Cells. Nat. Chem..

[17] Biffi G., Tannahill D., McCafferty J., Balasubramanian S. (2013). Quantitative Visualization of DNA G-Quadruplex Structures in Human Cells. Nat. Chem..

[18] Kosiol N., Juranek S., Brossart P., Heine A., Paeschke K. (2021). G-Quadruplexes: A Promising Target for Cancer Therapy. Mol. Cancer.

[19] Amato J., Iaccarino N., Randazzo A., Novellino E., Pagano B. (2014). Noncanonical DNA Secondary Structures as Drug Targets: The Prospect of the i-Motif. ChemMedChem.

[20] Verma A., Yadav V.K., Basundra R., Kumar A., Chowdhury S. (2009). Evidence of Genome-Wide G4 DNA-Mediated Gene Expression in Human Cancer Cells. Nucleic Acids Res..

[21] Shu B., Cao J., Kuang G., Qiu J., Zhang M., Zhang Y., Wang M., Li X., Kang S., Ou T.-M. (2018). Syntheses and Evaluation of New Acridone Derivatives for Selective Binding of Oncogene C- Myc Promoter i-Motifs in Gene Transcriptional Regulation. Chem. Commun..

[22] Brown R.V., Wang T., Chappeta V.R., Wu G., Onel B., Chawla R., Quijada H., Camp S.M., Chiang E.T., Lassiter Q.R. (2017). The Consequences of Overlapping G-Quadruplexes and i-Motifs in the Platelet-Derived Growth Factor Receptor β Core Promoter Nuclease Hypersensitive Element Can Explain the Unexpected Effects of Mutations and Provide Opportunities for Selective Targeting Of. J. Am. Chem. Soc..

[23] Satpathi S., Sappati S., Das K., Hazra P. (2019). Structural Characteristics Requisite for the Ligand-Based Selective Detection of i-Motif DNA. Org. Biomol. Chem..

[24] Masoud S.S., Nagasawa K. (2018). I-Motif-Binding Ligands and Their Effects on the Structure and Biological Functions of I-Motif. Chem. Pharm. Bull..

[25] Rostovtsev V.V., Green L.G., Fokin V.V., Sharpless K.B. (2002). A Stepwise Huisgen Cycloaddition Process: Copper(I)-Catalyzed Regioselective “Ligation” of Azides and Terminal Alkynes. Angew. Chem. Int. Ed..

[26] Saha P., Panda D., Dash J. (2019). The Application of Click Chemistry for Targeting Quadruplex Nucleic Acids. Chem. Commun..

[27] Tron G.C., Pirali T., Billington R.A., Canonico P.L., Sorba G., Genazzani A.A. (2008). Click Chemistry Reactions in Medicinal Chemistry: Applications of the 1,3-Dipolar Cycloaddition between Azides and Alkynes. Med. Res. Rev..

[28] Bonandi E., Christodoulou M.S., Fumagalli G., Perdicchia D., Rastelli G., Passarella D. (2017). The 1,2,3-Triazole Ring as a Bioisostere in Medicinal Chemistry. Drug Discov. Today.

[29] Moorhouse A.D., Santos A.M., Gunaratnam M., Moore M., Neidle S., Moses J.E. (2006). Stabilization of G-Quadruplex DNA by Highly Selective Ligands via Click Chemistry. J. Am. Chem. Soc..

[30] Ritson D.J., Moses J.E. (2012). A Fragment Based Click Chemistry Approach towards Hybrid G-Quadruplex Ligands: Design, Synthesis and Biophysical Evaluation. Tetrahedron.

[31] Sparapani S., Haider S.M., Doria F., Gunaratnam M., Neidle S. (2010). Rational Design of Acridine-Based Ligands with Selectivity for Human Telomeric Quadruplexes. J. Am. Chem. Soc..

[32] Drewe W.C., Neidle S. (2008). Click Chemistry Assembly of G-Quadruplex Ligands Incorporating a Diarylurea Scaffold and Triazole Linkers. Chem. Commun..

[33] Rodriguez R., Müller S., Yeoman J.A., Trentesaux C., Riou J.-F., Balasubramanian S. (2008). A Novel Small Molecule That Alters Shelterin Integrity and Triggers a DNA-Damage Response at Telomeres. J. Am. Chem. Soc..

[34] Müller S., Sanders D.A., Di Antonio M., Matsis S., Riou J.-F., Rodriguez R., Balasubramanian S. (2012). Pyridostatin Analogues Promote Telomere Dysfunction and Long-Term Growth Inhibition in Human Cancer Cells. Org. Biomol. Chem..

[35] Di Fonzo S., Amato J., D’Aria F., Caterino M., D’Amico F., Gessini A., Brady J.W., Cesàro A., Pagano B., Giancola C. (2020). Ligand Binding to G-Quadruplex DNA: New Insights from Ultraviolet Resonance Raman Spectroscopy. Phys. Chem. Chem. Phys..

[36] Pagano A., Iaccarino N., Abdelhamid M.A.S., Brancaccio D., Garzarella E.U., Di Porzio A., Novellino E., Waller Z.A.E., Pagano B., Amato J. (2018). Common G-Quadruplex Binding Agents Found to Interact With i-Motif-Forming DNA: Unexpected Multi-Target-Directed Compounds. Front. Chem..

[37] Hantzsch A. (1881). Condensationsprodukte Aus Aldehydammoniak Und Ketonartigen Verbindungen. Ber. Dtsch. Chem. Ges..

[38] Hantzsch A. (1882). Ueber Die Synthese Pyridinartiger Verbindungen Aus Acetessigäther Und Aldehydammoniak. Justus Liebig’s Ann. Chem..

[39] Tamaddon F., Razmi Z., Jafari A.A. (2010). Synthesis of 3,4-Dihydropyrimidin-2(1H)-Ones and 1,4-Dihydropyridines Using Ammonium Carbonate in Water. Tetrahedron Lett..

[40] Pagano B., Cosconati S., Gabelica V., Petraccone L., De Tito S., Marinelli L., La Pietra V., Saverio di Leva F., Lauri I., Trotta R. (2012). State-of-the-Art Methodologies for the Discovery and Characterization of DNA G-Quadruplex Binders. Curr. Pharm. Des..

[41] Dai J., Chen D., Jones R.A., Hurley L.H., Yang D. (2006). NMR Solution Structure of the Major G-Quadruplex Structure Formed in the Human BCL2 Promoter Region. Nucleic Acids Res..

[42] Kendrick S., Akiyama Y., Hecht S.M., Hurley L.H. (2009). The I-Motif in the Bcl-2 P1 Promoter Forms an Unexpectedly Stable Structure with a Unique 8:5:7 Loop Folding Pattern. J. Am. Chem. Soc..

[43] Ambrus A., Chen D., Dai J., Jones R.A., Yang D. (2005). Solution Structure of the Biologically Relevant G-Quadruplex Element in the Human c-MYC Promoter. Implications for G-Quadruplex Stabilization. Biochemistry.

[44] Dai J., Hatzakis E., Hurley L.H., Yang D. (2010). I-Motif Structures Formed in the Human c-MYC Promoter Are Highly Dynamic–Insights into Sequence Redundancy and I-Motif Stability. PLoS ONE.

[45] Dai J., Carver M., Yang D. (2008). Polymorphism of Human Telomeric Quadruplex Structures. Biochimie.

[46] Pagano B., Amato J., Iaccarino N., Cingolani C., Zizza P., Biroccio A., Novellino E., Randazzo A. (2015). Looking for Efficient G-Quadruplex Ligands: Evidence for Selective Stabilizing Properties and Telomere Damage by Drug-like Molecules. ChemMedChem.

[47] Phan A.T., Guéron M., Leroy J.-L. (2000). The Solution Structure and Internal Motions of a Fragment of the Cytidine-Rich Strand of the Human Telomere. J. Mol. Biol..

[48] Iaccarino N., Cheng M., Qiu D., Pagano B., Amato J., Di Porzio A., Zhou J., Randazzo A., Mergny J.-L. (2021). Effects of Sequence and Base Composition on the CD and TDS Profiles of I-DNA. Angew. Chem. Int. Ed..

[49] Wold S., Esbensen K., Geladi P. (1987). Principal Component Analysis. Chemom. Intell. Lab. Syst..

[50] Amato J., Morigi R., Pagano B., Pagano A., Ohnmacht S., De Magis A., Tiang Y.-P., Capranico G., Locatelli A., Graziadio A. (2016). Toward the Development of Specific G-Quadruplex Binders: Synthesis, Biophysical, and Biological Studies of New Hydrazone Derivatives. J. Med. Chem..

[51] Amato J., Miglietta G., Morigi R., Iaccarino N., Locatelli A., Leoni A., Novellino E., Pagano B., Capranico G., Randazzo A. (2020). Monohydrazone Based G-Quadruplex Selective Ligands Induce DNA Damage and Genome Instability in Human Cancer Cells. J. Med. Chem..

[52] Kaiser C.E., Van Ert N.A., Agrawal P., Chawla R., Yang D., Hurley L.H. (2017). Insight into the Complexity of the I-Motif and G-Quadruplex DNA Structures Formed in the KRAS Promoter and Subsequent Drug-Induced Gene Repression. J. Am. Chem. Soc..

[53] Mayer M., Meyer B. (1999). Characterization of Ligand Binding by Saturation Transfer Difference NMR Spectroscopy. Angew. Chem. Int. Ed..

[54] Giancola C., Pagano B. (2013). Energetics of Ligand Binding to G-Quadruplexes. Top. Curr. Chem..

[55] King J.J., Irving K.L., Evans C.W., Chikhale R.V., Becker R., Morris C.J., Peña Martinez C.D., Schofield P., Christ D., Hurley L.H. (2020). DNA G-Quadruplex and i-Motif Structure Formation Is Interdependent in Human Cells. J. Am. Chem. Soc..

[56] Böcker R.H., Guengerich F.P. (1986). Oxidation of 4-Aryl- and 4-Alkyl-Substituted 2,6-Dimethyl-3,5-Bis(Alkoxycarbonyl)-1,4-Dihydropyridines by Human Liver Microsomes and Immunochemical Evidence for the Involvement of a Form of Cytochrome P-450. J. Med. Chem..

[57] Loev B., Snader K.M. (1965). The Hantzsch Reaction. I. Oxidative Dealkylation of Certain Dihydropyridines. J. Org. Chem..

[58] Shahabi D., Amrollahi M.A. (2014). Synthesis of Some Novel and Water-Soluble 2,4,6-Substituted 3,5-Dihydroxymethylpyridines. Bulg. Chem. Commun..

[59] Amato J., Pagano A., Cosconati S., Amendola G., Fotticchia I., Iaccarino N., Marinello J., De Magis A., Capranico G., Novellino E. (2017). Discovery of the First Dual G-Triplex/G-Quadruplex Stabilizing Compound: A New Opportunity in the Targeting of G-Rich DNA Structures?. Biochim. Biophys. Acta—Gen. Subj..

[60] Cantor C.R., Warshaw M.M., Shapiro H. (1970). Oligonucleotide Interactions. III. Circular Dichroism Studies of the Conformation of Deoxyoligonucleolides. Biopolymers.

[61] Iaccarino N., Di Porzio A., Amato J., Pagano B., Brancaccio D., Novellino E., Leardi R., Randazzo A. (2019). Assessing the Influence of pH and Cationic Strength on I-Motif DNA Structure. Anal. Bioanal. Chem..

[62] Musumeci D., Amato J., Zizza P., Platella C., Cosconati S., Cingolani C., Biroccio A., Novellino E., Randazzo A., Giancola C. (2017). Tandem Application of Ligand-Based Virtual Screening and G4-OAS Assay to Identify Novel G-Quadruplex-Targeting Chemotypes. Biochim. Biophys. Acta—Gen. Subj..

[63] Yang S.Y., Chang E.Y.C., Lim J., Kwan H.H., Monchaud D., Yip S., Stirling P.C., Wong J.M.Y. (2021). G-Quadruplexes Mark Alternative Lengthening of Telomeres. NAR Cancer.

