# Supplementary material for: Synthesis and Characterization of Bis-Triazolyl-Pyridine Derivatives as Noncanonical DNA-Interacting Compounds
Source: Int J Mol Sci. 2021 Nov 4;22(21):11959. doi: 10.3390/ijms222111959 (PMC8584640; doi:10.3390/ijms222111959)
Supplement: Supplementary file 1 [file ijms-22-11959-s001.zip › ijms-1420140-supplementary.pdf]

Supplementary Material

Synthesis and Characterization of Bis-Triazolyl-Pyridine Derivatives as Noncanonical DNA-Interacting Compounds

Anna Di Porzio [1,†], Ubaldina Galli [2,†], Jussara Amato [1], Pasquale Zizza [3], Sara Iachettini [3], Nunzia Iaccarino [1], Simona Marzano [1], Federica Santoro[1], Diego Brancaccio [1], Alfonso Carotenuto [1], Stefano De Tito [4,5], Annamaria Biroccio [3], Bruno Pagano [1], Gian Cesare Tron [2,*] and Antonio Randazzo [1,*]

[1] Department of Pharmacy, University of Naples Federico II, Via D. Montesano 49, 80131 Naples, Italy; anna.diporzio@unina.it (A.D.); jussara.amato@unina.it (J.A.); nunzia.iaccarino@unina.it (N.I.); simona.marzano@unina.it (S.M.); federica.santoro@unina.it (F.S.); diego.brancaccio@unina.it (D.B.); alfonso.carotenuto@unina.it (A.C.); bruno.pagano@unina.it (B.P.)

[2] Department of Pharmaceutical Sciences, University of Piemonte Orientale, Largo Donegani 2/3, 28100 Novara, Italy; ubaldina.galli@uniupo.it (U.G.)

[3] Oncogenomic and Epigenetic Unit, IRCCS-Regina Elena National Cancer Institute, 00144 Rome, Italy; pasquale.zizza@ifo.gov.it (P.Z.); sara.iachettini@ifo.gov.it (S.I.); annamaria.biroccio@ifo.gov.it (A.B.)

[4] Molecular Cell Biology of Autophagy, The Francis Crick Institute, 1 Midland Road, London NW1 1AT, UK; stefano.de-tito@crick.ac.uk

[5] Institute of Experimental Endocrinology and Oncology, National Research Council, 80131 Naples, Italy

[†] These authors contributed equally to the work.
* Correspondence: antonio.randazzo@unina.it (A.R.), Tel.: +39081678514; giancesare.tron@uniupo.it (G.C.T.), Tel.: +390321375857

Table of contents | **Page**

Table of contents	Page
General Procedures for the Synthesis of Alkynes	S3
Figure S1	S10
Figure S2	S11
Figure S3	S12
Figure S4	S13
Figure S5	S14
Figure S6	S15
Figure S7	S16
Figure S8	S17
Figure S9	S18
Table S1	S19
Table S2	S20
Table S3	S21
Figure S10	S22
Figure S11	S22
Figure S12	S23
Figure S13	S23
Figure S14	S24
Figure S15	S25
Figure S16	S26
Figure S17	S27
Figure S18	S28
Figure S19	S29
Figure S20	S30
Figure S21	S31
Figure S22	S32
Figure S23	S33
Figure S24	S34
Figure S25	S35
Figure S26	S36
Figure S27	S37
Figure S28	S38
Figure S29	S39
Figure S30	S40
Figure S31	S41
Figure S32	S42
Figure S33	S43
Figure S34	S44
Figure S35	S45
Figure S36	S46
Figure S37	S47
Figure S38	S48
Figure S39	S49
Figure S40	S50
Figure S41	S51
Figure S42	S52
Figure S43	S53
Figure S44	S54
Figure S45	S55
Figure S46	S56
Figure S47	S57
Figure S48	S58
References	S59

General Procedures for the Synthesis of Alkynes

*General procedure A for the synthesis of alkynes **5a-14a** (series a)*

To a solution of the corresponding secondary amine (2 equiv) in dry THF (1.5 M), K_2CO_3 (2.5 equiv) and propargyl bromide (1 equiv) were added. The reaction mixture was stirred at 25 °C for 12 h under nitrogen, then was diluted with water and extracted with EtOAc (x2). The combined organic layers were washed with brine (x1), dried over anhydrous sodium sulfate and concentrated *in vacuo* to obtain the corresponding products (Scheme S1).

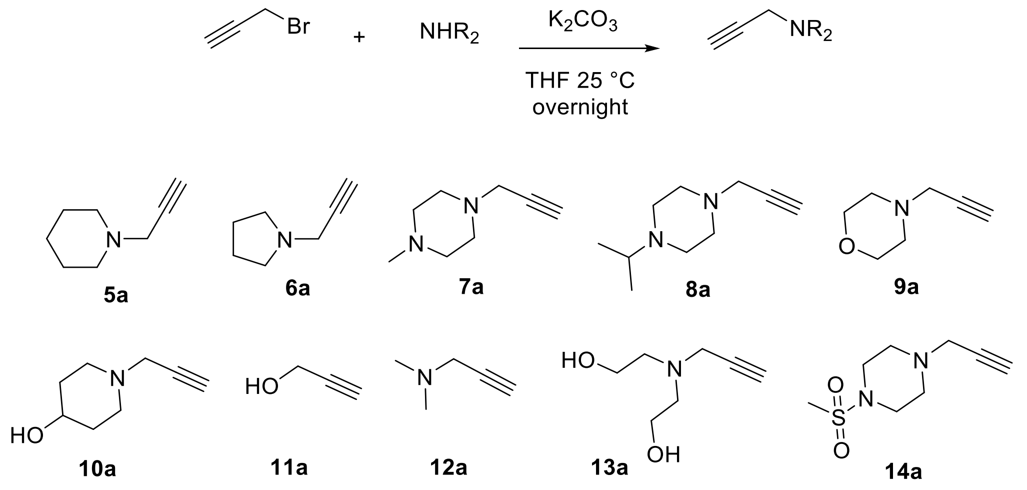

Scheme S1. General procedure for the synthesis of alkynes **5a-14a**.

1-(prop-2-yn-1-yl)piperidine (5a)

The title compound was prepared from piperidine according to general procedure A and was used in the next step without further purification. Light brown oil: yield 68%; **IR** (KBr) 3114, 1671, 1608, 1392, 1370, 1276, 1245, 1134, 1075, 847, 584 cm^{-1}; **^{1}H-NMR** (300 MHz, $CDCl_3$) δ 3.21 (d, J = 1.83 Hz, 2-H) 2.46 (t, J = 4.9 Hz, 4-H), 2.19 (t, J = 1.8 Hz, 1-H), 1.58 (quint, J = 5.5, 4-H), 1.39 (quint, J = 4.9 Hz, 2-H) ppm; **^{13}C-NMR** (75 MHz, $CDCl_3$) δ 79.4, 72.9, 53.2, 47.7, 26.0, 24.0 ppm. The spectral data were consistent with those reported previously [1].

1-(prop-2-yn-1-yl)pyrrolidine (6a)

The title compound was prepared from pyrrolidine according to general procedure A and was used in the next step without further purification. Light brown oil: yield 26%; **IR** (KBr) 3302, 3200, 2968, 2791, 1661, 1432, 1127, 651 cm^{-1}; **^{1}H-NMR** (300 MHz, $CDCl_3$) δ 3.32 (d, J = 2.5 Hz, 2-H), 2.54-2.50 (m, 4-H), 2.13 (t, J = 2.5, Hz, 1-H), 1.73-1.70 (m, 4-H) ppm; **^{13}C-NMR** (75 MHz, $CDCl_3$) δ 79.5, 72.2, 52.3, 42.8, 23.7 ppm. The spectral data were consistent with those reported previously [1].

methyl-4-(prop-2-yn-1-yl)piperazine (7a)

The title compound was prepared from *N*-methylpiperazine according to general procedure A and was used in the next step without further purification. Yellow oil: yield 53%; **IR** (KBr) 3292, 3187, 2936, 2797, 1674, 1456, 1283, 1163, 1013, 819 cm^{-1}; **^{1}H-NMR** (300 MHz, $CDCl_3$) δ 3.24 (d, J = 2.5

Hz, 2-H), 2.56 (br s, 4-H), 2.43 (br s, 4-H), 2.23 (br s, 3-H), 2.19 (t, J = 2.5 Hz, 1-H) ppm; **^{13}C-NMR** (75 MHz, $CDCl_3$) δ 78.6, 73.1, 54.8, 51.7, 46.7, 45.9 ppm. The spectral data were consistent with those reported previously [1].

1-isopropyl-4-(prop-2-yn-1-yl)piperazine (8a)

The title compound was prepared from *N*-isopropylpiperazine according to general procedure A and was used in the next step without further purification. Yellow oil: yield 87%; **IR** (KBr) 3306, 3207, 2965, 2813, 1672, 1611, 1453, 1332, 1178, 1012 cm-^{1}; **^{1}H-NMR** (300 MHz, $CDCl_3$) δ 3.08 (br s, 2-H), 2.40 (br s, 9-H), 2.07 (s, 1-H), 0.84 (br s, 6-H) ppm; **^{13}C-NMR** (75 MHz, $CDCl_3$) δ 78.7, 72.9, 54.1, 52.1, 48.2, 46.6, 18.4 ppm.

4-(prop-2-yn-1-yl)morpholine (9a)

The title compound was prepared from morpholine according to general procedure A and was used in the next step without further purification. Yellow oil: yield 42%; **IR** (KBr) 3289, 2962, 2857, 1677, 1453, 1290, 1116, 1006, 861 cm^{-1}; **^{1}H-NMR** (300 MHz, $CDCl_3$) δ 3.66-3.61 (m, 4-H), 3.21-3.15 (m, 2-H), 2.49-2.44 (m, 4-H), 2.19 (d, J = 1.8 Hz, 1-H) ppm; **^{13}C-NMR** (75 MHz, $CDCl_3$) δ 78.4, 73.4, 66.7, 52.1, 47.1 ppm. The spectral data were consistent with those reported previously [1].

1-(prop-2-yn-1-yl)piperidin-4-ol (10a)

The title compound was prepared from 4-hydroxypiperidine according to general procedure A and was used in the next step without further purification. Yellow solid: yield 89%; mp 103-104 °C; **IR** (KBr) 3197, 2942, 2794, 2113, 1450, 1329, 1070, 1022, 783 cm^{-1}; **^{1}H-NMR** (300 MHz, $CDCl_3$) δ 3.62-3.58 (m, 1-H), 3.24 (d, J = 2.5 Hz, 2-H), 2.88 (br s, 1-H), 2.76-2.72 (m, 2-H), 2.33-2.26 (m, 2-H), 2.21 (t, J = 2.5 Hz, 1-H), 1.88-1.84 (m, 2-H), 1.61-1.57 (m, 2-H) ppm; **^{13}C-NMR** (75 MHz, $CDCl_3$) δ 78.8, 73.3, 67.2, 49.9, 46.9, 34.3 ppm. The spectral data were consistent with those reported previously [2].

propargyl alcohol (11a) and **1-dimethylamino-2-propyne (12a)** were commercially available.

2,2'-(prop-2-yn-1-ylazanediyl)bis(ethan-1-ol) (13a)

The compound was prepared as described previously [3].

1-(methylsulfonyl)-4-(prop-2-yn-1-yl)piperazine (14a)

The title compound was prepared from 1-methylsulfonyl-piperazine according to general procedure A and the crude was purified by column chromatography using EtOAc/MeOH 9:1 + 1% conc. NH_4OH as eluant. Yellow oil: yield 42%; **IR** (KBr) 3289, 2962, 2857, 1677, 1453, 1290, 1116, 1006, 861 cm^{-1}; **^{1}H-NMR** (300 MHz, $CDCl_3$) δ 3.66-3.61 (m, 4-H), 3.21-3.15 (m, 2-H), 2.49-2.44 (m, 4-H), 2.19 (d, J = 1.8 Hz, 1-H) ppm; **^{13}C-NMR** (75 MHz, $CDCl_3$) δ 78.4, 73.4, 66.7, 52.1, 47.1 ppm.

*General procedure B for the synthesis of alkynes **5b-11b** (series b)*

To a solution of the corresponding secondary amine (2 equiv) in dry THF (1.5 M), K_2CO_3 (2.5 equiv) and 4-bromo-1-butyne (1 equiv) were added. The reaction mixture was stirred at reflux overnight under nitrogen, then was diluted with water and extracted with EtOAc (x3). The combined organic

layers were washed with brine (x1), dried over anhydrous sodium sulfate and concentrated *in vacuo* to obtain the corresponding products (Scheme S2).

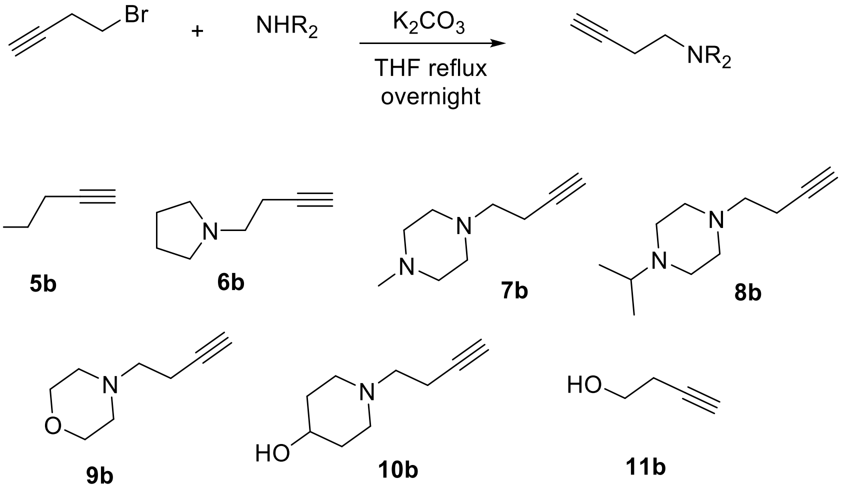

Scheme S2. General procedure for the synthesis of alkynes **5b-11b**.

1-(but-3-yn-1-yl)piperidine (5b)

The title compound was prepared from piperidine according to general procedure B and was used in the next step without further purification. Orange oil: yield 51%; **^{1}H-NMR** (300 MHz, $CDCl_3$) δ 2.53 (td, *J*=8.0/2.1 Hz, 2-H), 2.37-2.32 (m, 6-H), 1.93 (t, *J*=2.5 Hz, 1-H), 1.60-1.48 (m, 4-H), 1.40-1.38 (m, 2-H) ppm; **^{13}C-NMR** (75 MHz, $CDCl_3$) δ 82.6, 68.7, 57.6, 53.9, 25.6, 24.0, 16.4 ppm. The spectral data were consistent with those reported previously [4].

1-(but-3-yn-1-yl)pyrrolidine (6b)

The title compound was prepared from pyrrolidine according to general procedure B and was used in the next step without further purification. Yellow oil: yield 20%; **^{1}H-NMR** (300 MHz, $CDCl_3$) δ 2.63 (td, *J*=7.6/2.2 Hz, 2-H), 2.50 (br s, 4-H), 2.40-2.36 (m, 2-H), 1.95 (t, *J* = 2.6 Hz, 1-H), 1.77-1.74 (m, 4-H) ppm; **^{13}C-NMR** (75 MHz, $CDCl_3$) δ 82.3, 68.6, 54.6, 53.6, 23.1, 18.4 ppm. The spectral data were consistent with those reported previously [4].

1-(but-3-yn-1-yl)-4-methylpiperazine (7b)

The title compound was prepared from *N*-methylpiperazine according to general procedure B and the crude was purified by column chromatography using EtOAc/CH_3OH 5:5 as eluant. Yellow oil: yield 34%; **^{1}H-NMR** (300 MHz, $CDCl_3$) δ 2.34-2.06 (m, 12-H), 2.00 (br s, 3-H), 1.75 (t, *J* = 2.5 Hz, 1-H) ppm; **^{13}C-NMR** (75 MHz, $CDCl_3$) δ 82.8, 68.9, 56.5, 54.5, 52.3, 45.6, 16.3 ppm. The spectral data were consistent with those reported previously [4].

1-(but-3-yn-1-yl)-4-isopropylpiperazine (8b)

The title compound was prepared from *N*-isopropylpiperazine according to general procedure B and the crude was purified by column chromatography using EtOAc/CH_3OH 95:5 as eluant. Yellow oil: yield 60%; **^{1}H-NMR** (300 MHz, $CDCl_3$) δ 2.49-2.35 (m, 11-H), 2.18 (td, *J*= 6.4/1.8 Hz, 2-H), 1.81

(t, *J*= 2.5 Hz, 1-H), 0.85 (dd, *J* = 6.4/2.2 Hz, 6-H) ppm; **^{13}C-NMR** (75 MHz, $CDCl_3$) δ 82.4, 68.9, 56.8, 54.1, 52.9, 48.3, 18.4, 16.4 ppm.

4-(but-3-yn-1-yl)morpholine (9b)

The title compound was prepared from morpholine according to general procedure B and was used in the next step without further purification. Yellow oil; yield 58%; **IR** (neat) 3291, 2951, 2855, 2811, 2118, 1458, 1277, 1138, 1114, 1070, 1006, 866, 632, cm^{-1}; **^{1}H-NMR** (300 MHz, $CDCl_3$) δ 3.57-3.56 (m, 4-H), 2.46-2.24 (m, 8-H), 1.88 (s, 1-H) ppm; **^{13}C-NMR** (75 MHz, $CDCl_3$) δ 82.4, 69.1, 66.7, 57.2, 53.3, 16.5 ppm. The spectral data were consistent with those reported previously [4].

1-(but-3-yn-1-yl)piperidin-4-ol (10b)

The title compound was prepared from 4-hydroxypiperidine according to general procedure B and was extracted with EtOAc (x5). It was used in the next step without further purification. Yellow oil; yield 18%; **IR** (neat) 3290, 2939, 2815, 2117, 1454, 1366, 1283, 1115, 1067, 632, cm^{-1}; **^{1}H-NMR** (300 MHz, $CDCl_3$) δ 3.69-3.63 (m, 1-H), 2.79-2.76 (m, 2-H), 2.60-2.33 (m, 5-H), 2.22-2.15 (m, 2-H), 1.97 (s, 1-H), 1.89-1.85 (m, 2-H), 1.61-1.55 (m, 2-H) ppm; **^{13}C-NMR** (75 MHz, $CDCl_3$) δ 83.1, 69.2, 67.7, 57.0, 50.9, 34.4, 17.0 ppm.

3-butyn-1-ol (11b) was commercially available.

General procedure C for the synthesis of alkynes ***5c-11c*** *(series c)*

A stirred mixture of pent-4-yn-1-yl 4-methylbenzenesulfonate (1.2 equiv) prepared as described previously [5]. K_2CO_3 (2 equiv) and the corresponding secondary amine (1 equiv) in DMF (0.5 M) was heated at 80 °C for 3 h under nitrogen. Then the reaction mixture was diluted with water and extracted with EtOAc (x3). The combined organic layers were washed with brine (x2), dried over anhydrous sodium sulfate and concentrated *in vacuo* to obtain the corresponding products (Scheme S3).

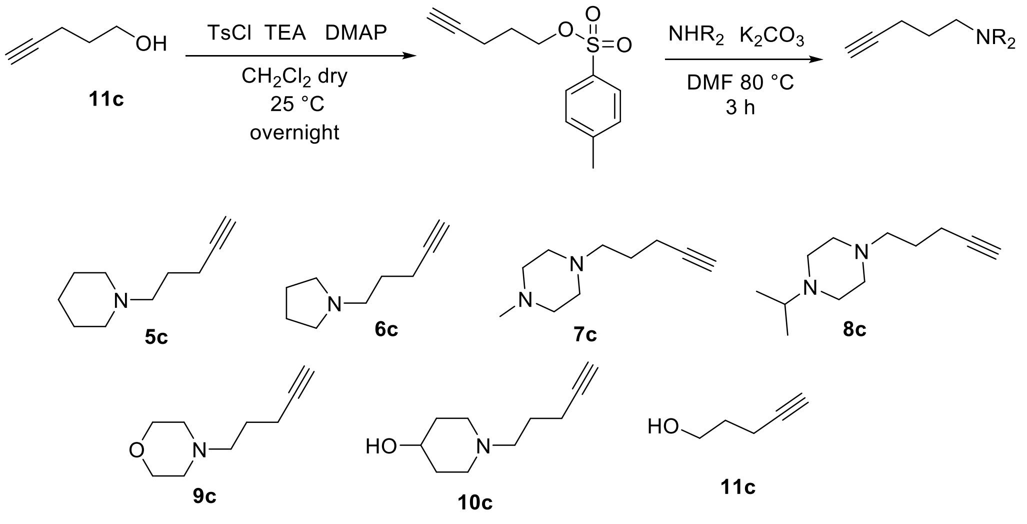

Scheme S3. General procedure for the synthesis of alkynes **5c-11c**.

1-(pent-4-yn-1-yl)piperidine (5c)

The title compound was prepared from piperidine according to general procedure C and was used in the next step without further purification. Orange oil: yield 86%; **^{1}H-NMR** (300 MHz, $CDCl_3$) δ 2.25-2.21 (m, 6-H), 2.06 (td, *J* =7.0/2.7 Hz, 2-H), 1.80 (t, *J* =2.7 Hz, 1-H), 1.56 (quint, *J* = 7.3 Hz, 2-H), 1.47-1.41 (m, 4-H), 1.30-1.28 (m, 2-H) ppm; **^{13}C-NMR** (75 MHz, $CDCl_3$) δ 84.0, 68.2, 58.0, 54.4, 25.9, 25.7, 24.3, 16.4 ppm. The spectral data were consistent with those reported previously [6].

1-(pent-4-yn-1-yl)pyrrolidine (6c)

The title compound was prepared from pyrrolidine according to general procedure C and was used in the next step without further purification. Brown oil: yield 74%; **^{1}H-NMR** (300 MHz, $CDCl_3$) δ 2.48-2.42 (m, 6-H), 2.17 (quint, *J* =6.7 Hz, 2-H), 1.88 (t, *J* =2.7 Hz, 1-H), 1.70-1.62 (m, 6-H) ppm; **^{13}C-NMR** (75 MHz, $CDCl_3$) δ 84.2, 68.4, 55.3, 54.1, 27.9, 23.4, 16.6 ppm. The spectral data were consistent with those reported previously [6].

1-methyl-4-(pent-4-yn-1-yl)piperazine (7c)

The title compound was prepared from *N*-methylpiperazine according to general procedure C and was used in the next step without further purification. Orange oil; yield 68%; **^{1}H-NMR** (300 MHz, $CDCl_3$) δ 2.20-2.16 (m, 10-H), 2.04-1.95 (m, 5-H), 1.74 (t, *J* =2.7 Hz, 1-H), 1.46 (quint, *J* =7.3 Hz, 2-H) ppm; **^{13}C-NMR** (75 MHz, $CDCl_3$) δ 83.8, 68.2, 56.9, 54.8, 52.9, 45.8, 25.5, 16.1 ppm.

1-isopropyl-4-(pent-4-yn-1-yl)piperazine (8c)

The title compound was prepared from *N*-isopropylpiperazine according to general procedure C and was used in the next step without further purification. Orange oil; yield 85%; **^{1}H-NMR** (300 MHz, $CDCl_3$) δ 2.39-2.09 (m, 11-H), 1.92 (td, *J* =6.7/2.4 Hz, 2-H), 1.68 (t, *J* =2.4 Hz, 1-H), 0.74 (br d, 6-H) ppm; **^{13}C-NMR** (75 MHz, $CDCl_3$) δ 83.6, 68.1, 56.9, 53.9, 53.1, 48.2, 25.3, 18.2, 15.9 ppm.

4-(pent-4-yn-1-yl)morpholine (9c)

The title compound was prepared from morpholine according to general procedure C and was used in the next step without further purification. Orange oil; yield 60%; **IR** (neat) 3292, 2952, 2854, 2809, 2117, 1456, 1268, 1137, 1115, 1010, 866, 629 cm^{-1}; **^{1}H-NMR** (300 MHz, $CDCl_3$) δ 3.64 (br s, 4-H), 2.38 (br d, 6-H), 2.21-2.17 (m, 2-H), 1.89 (s, 1-H), 1.65 (quint, *J* = 7.0 Hz, 2H) ppm; **^{13}C-NMR** (75 MHz, $CDCl_3$) δ 84.2, 68.5, 67.1, 57.7, 53.8, 25.6, 16.4 ppm.

1-(pent-4-yn-1-yl)piperidin-4-ol (10c)

The title compound was prepared from 4-hydroxypiperidine according to general procedure C and was extracted with EtOAc (x5). The crude was purified by column chromatography using EtOAc/MeOH 8:2 as eluant. Yellow solid; yield 41%; mp 86-89 °C; **IR** (KBr) 3187, 2922, 2803, 2772, 1428, 1360, 1241, 1187, 1136, 1079, 746, 723, cm^{-1}; **^{1}H-NMR** (300 MHz, $CDCl_3$) δ 3.63-3.58 (m, 1-H), 2.73-2.69 (m, 2-H), 2.36 (t, *J* = 7.1 Hz, 2-H), 2.18-2.04 (m, 4-H), 1.90 (s, 1-H), 1.84-1.80 (m, 2-H), 1.70-1.50 (m, 4-H) ppm; **^{13}C-NMR** (75 MHz, $CDCl_3$) δ 84.2, 68.6, 67.7, 57.3, 51.2, 34.4, 26.0, 16.5 ppm; **MS (ESI)** m/z 168 $[M+H]^+$.

4-pentyn-1-ol (11c) was commercially available.

General procedure D for the synthesis of alkynes ***5d-11d*** *(series d)*

A stirred mixture of hex-5-yn-1-yl 4-methylbenzenesulfonate (1.2 equiv) prepared as described previously [5]. K_2CO_3 (2 equiv) and the corresponding secondary amine (1 equiv) in DMF (0.5 M) was heated at 80 °C for 3 h under nitrogen. Then the reaction mixture was diluted with water and extracted with EtOAc (x3). The combined organic layers were washed with brine (x2), dried over anhydrous sodium sulfate and concentrated *in vacuo* to obtain the corresponding products (Scheme S4).

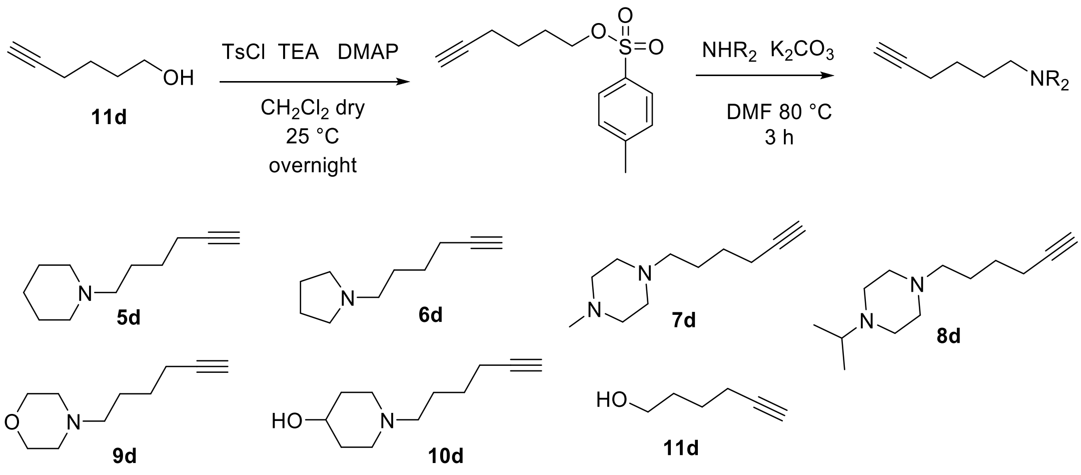

Scheme S4. General procedure for the synthesis of alkynes **5d-11d**.

1-(hex-5-yn-1-yl)piperidine (5d)

The title compound was prepared from piperidine according to general procedure D and was used in the next step without further purification. Orange oil; quantitative yield; **IR** (neat) 3309, 2933, 2855, 2764, 2117, 1681, 1442, 1154, 1121, 1089, 624, cm^{-1}; **^{1}H-NMR** (300 MHz, $CDCl_3$) δ 2.15-1.98 (m, 8-H), 1.76 (s, 1-H), 1.37-1.22 (m, 10-H) ppm; **^{13}C-NMR** (75 MHz, $CDCl_3$) δ 84.0, 68.2, 58.6, 54.3, 26.4, 25.7, 24.2, 18.1 ppm; **MS (ESI)** m/z 166 $[M+H]^+$.

1-(hex-5-yn-1-yl)pyrrolidine (6d)

The title compound was prepared from pyrrolidine according to general procedure D. Considering the volatility of the product, diethyl ether was used as the extraction solvent, which was evaporated under vacuum at a temperature not exceeding 40 °C. The compound was used in the next step without further purification. Yellow oil: quantitative yield; **IR** (neat) 3308, 2937, 2875, 2787, 2117, 1699, 1457, 1429, 1154, 1126, 624, 549, cm^{-1}; **^{1}H-NMR** (300 MHz, $CDCl_3$) δ 2.30-2.23 (m, 6-H), 2.04 (br s, 2-H), 1.77 (s, 1-H), 1.59-1.58 (m, 2-H), 1.45-1.43 (m, 2-H) ppm; **^{13}C-NMR** (75 MHz, $CDCl_3$) δ 84.0, 68.3, 55.8, 54.0, 28.0, 26.5, 23.2, 18.2 ppm; **MS (ESI)** m/z 152 $[M+H]^+$.

1-(hex-5-yn-1-yl)-4-methylpiperazine (7d)

The title compound was prepared from *N*-methylpiperazine according to general procedure D. The crude was purified by column chromatography using EtOAc/MeOH 8:2 as eluant. Yellow oil: yield 35%; **IR** (neat) 3307, 2936, 2793, 1701, 1458, 1284, 1237, 1162, 1147, 1014, 626, cm^{-1}; **^{1}H-NMR** (300 MHz, $CDCl_3$) δ 2.30 (br s, 6-H), 2.22-2.17 (m, 3-H), 2.14-2.05 (m, 6-H), 1.80 (br s, 1-H), 1.47-

1.37 (m, 4-H) ppm; **^{13}C-NMR** (75 MHz, $CDCl_3$) δ 84.1, 68.4, 57.9, 55.0, 53.1, 45.9, 26.3, 25.8, 18.2 ppm; **MS (ESI)** m/z 181 $[M+H]^+$.

1-(hex-5-yn-1-yl)-4-isopropylpiperazine (8d)

The title compound was prepared from *N*-isopropylpiperazine according to general procedure D and was purified by column chromatography using EtOAc/MeOH 7:3 as eluant. Yellow oil: yield: 62%; **IR** (neat) 3310, 2938, 2809, 2117, 1702, 1455, 1361, 1245, 1177, 1132, 983, 625, cm^{-1}; **^{1}H-NMR** (300 MHz, $CDCl_3$) δ 2.52-2.40 (m, 9-H), 2.19-2.17 (m, 2-H), 2.08-2.06 (m, 2-H), 1.80 (s, 1-H), 1.42-1.39 (m, 4-H), 0.91-0.89 (m, 6-H) ppm; **^{13}C-NMR** (75 MHz, $CDCl_3$) δ 84.1, 68.3, 58.0, 54.3, 53.4, 48.5, 26.4, 25.8, 18.5, 18.2 ppm; **MS (ESI)** m/z 209 $[M+H]^+$.

4-(hex-5-yn-1-yl)morpholine (9d)

The title compound was prepared from morpholine according to general procedure D. Considering the volatility of the product, diethyl ether was used as the extraction solvent, which was evaporated under vacuum at a temperature not exceeding 40 °C. The compound was used in the next step without further purification. Yellow oil: quantitative yield; **IR** (neat) 3293, 2941, 2858, 2808, 2769, 1701, 1446, 1242, 1115, 865, 628, cm^{-1}; **^{1}H-NMR** (300 MHz, $CDCl_3$) δ 3.45-3.36 (m, 4-H), 2.17-2.04 (m, 6-H), 1.96 (br s, 2-H), 1.75 (t, J = 2.4 Hz, 1-H), 1.32 (br s, 4-H) ppm; **^{13}C-NMR** (75 MHz, $CDCl_3$) δ 83.8, 68.3, 66.4, 58.0, 53.3, 25.9, 25.1, 17.9 ppm; **MS (ESI)** m/z 168 $[M+H]^+$. The spectral data were consistent with those reported previously [7].

1-(hex-5-yn-1-yl)piperidin-4-ol (10d)

The title compound was prepared from 4-hydroxypiperidine according to general procedure D and was extracted with EtOAc (x5). The crude was purified by column chromatography using EtOAc/MeOH 8:2 as eluant. Yellow oil: yield 66%; **IR** (neat) 3299, 2939, 2863, 2810, 2772, 2115, 1453, 1366, 1242, 1065, 731, 626, cm^{-1}; **^{1}H-NMR** (300 MHz, $CDCl_3$) δ 3.67-3.66 (m, 1-H), 2.77-2.75 (m, 2-H), 2.33-2.31 (m, 2-H), 2.19-2.12 (m, 4-H), 1.92-1.86 (m, 3-H), 1.59-1.52 (m, 6-H) ppm; **^{13}C-NMR** (75 MHz, $CDCl_3$) δ 83.9, 68.4, 66.9, 57.6, 50.9, 33.9, 26.2, 25.6, 18.0 ppm; **MS (ESI)** m/z 182 $[M+H]^+$.

5-hexyn-1-ol (11d) was commercially available.

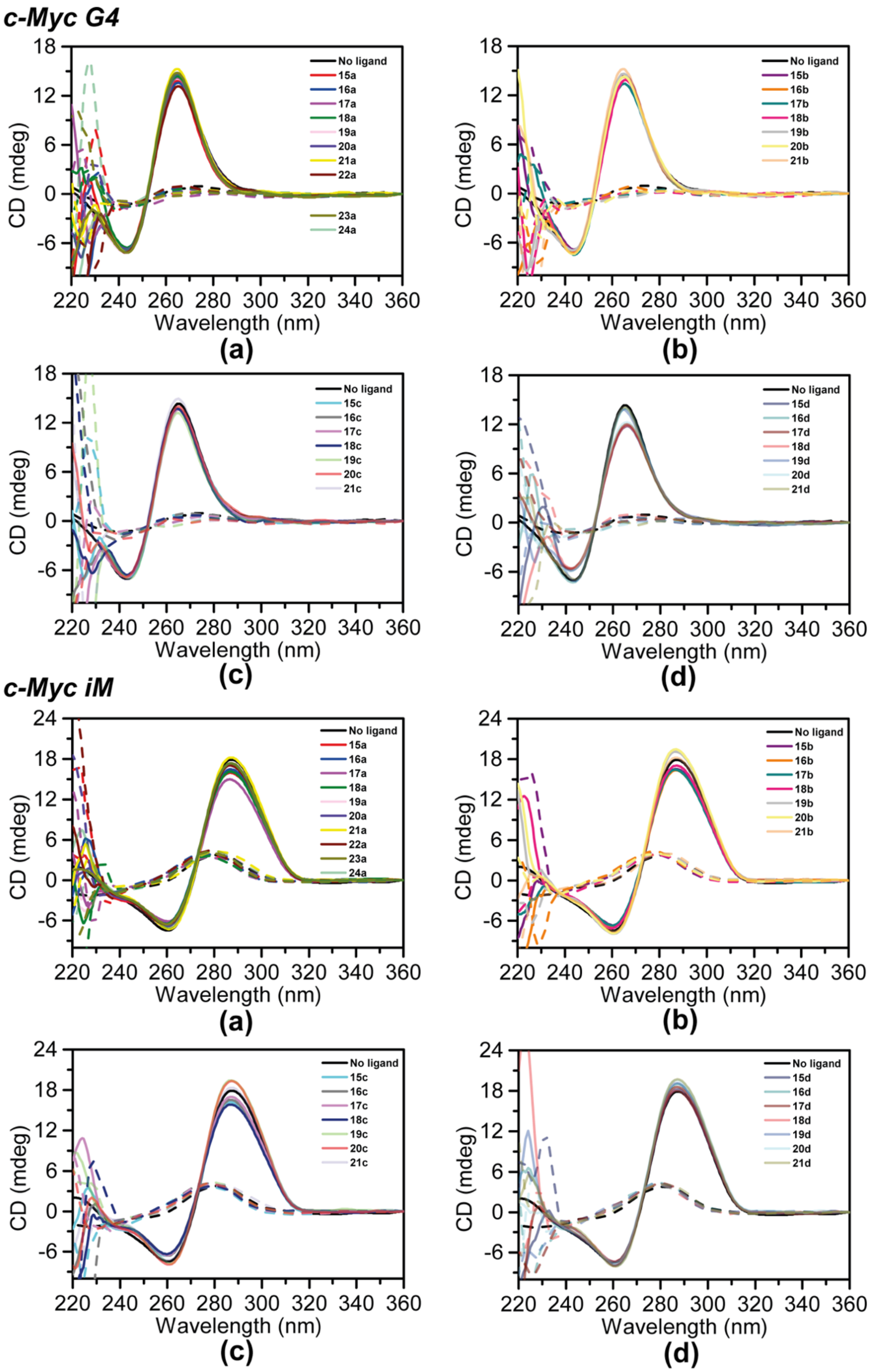

Figure S1. CD spectra of *c-Myc G4* and *c-Myc iM* in the absence (black line) and presence of 10 equivalents of compounds from series a **(a)**, b **(b)**, c **(c)** and d **(d)**. The spectra were acquired at 20 °C (solid lines) and 100 °C (dashed lines) for *c-Myc G4*, and at 5° (solid lines) and 90 °C (dashed lines) for *c-Myc iM*.

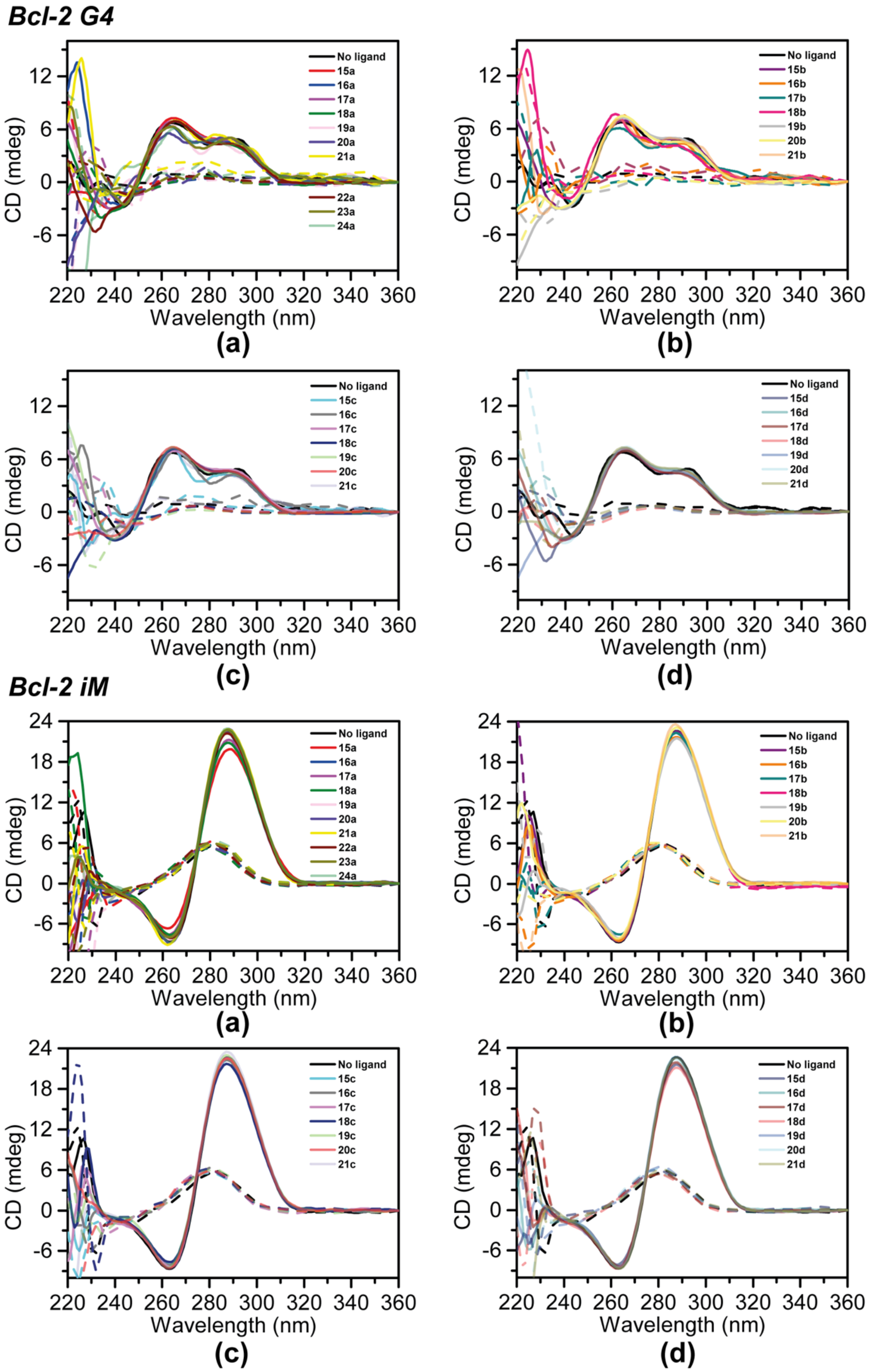

Figure S2. CD spectra of *Bcl-2 G4* and *Bcl-2 iM* in the absence (black line) and presence of 10 equivalents of compounds from series a **(a)**, b **(b)**, c **(c)** and d **(d)**. The spectra were acquired at 20 °C (solid lines) and 100 °C (dashed lines) for *Bcl-2 G4*, and at 5° (solid lines) and 90 °C (dashed lines) for *Bcl-2 iM*.

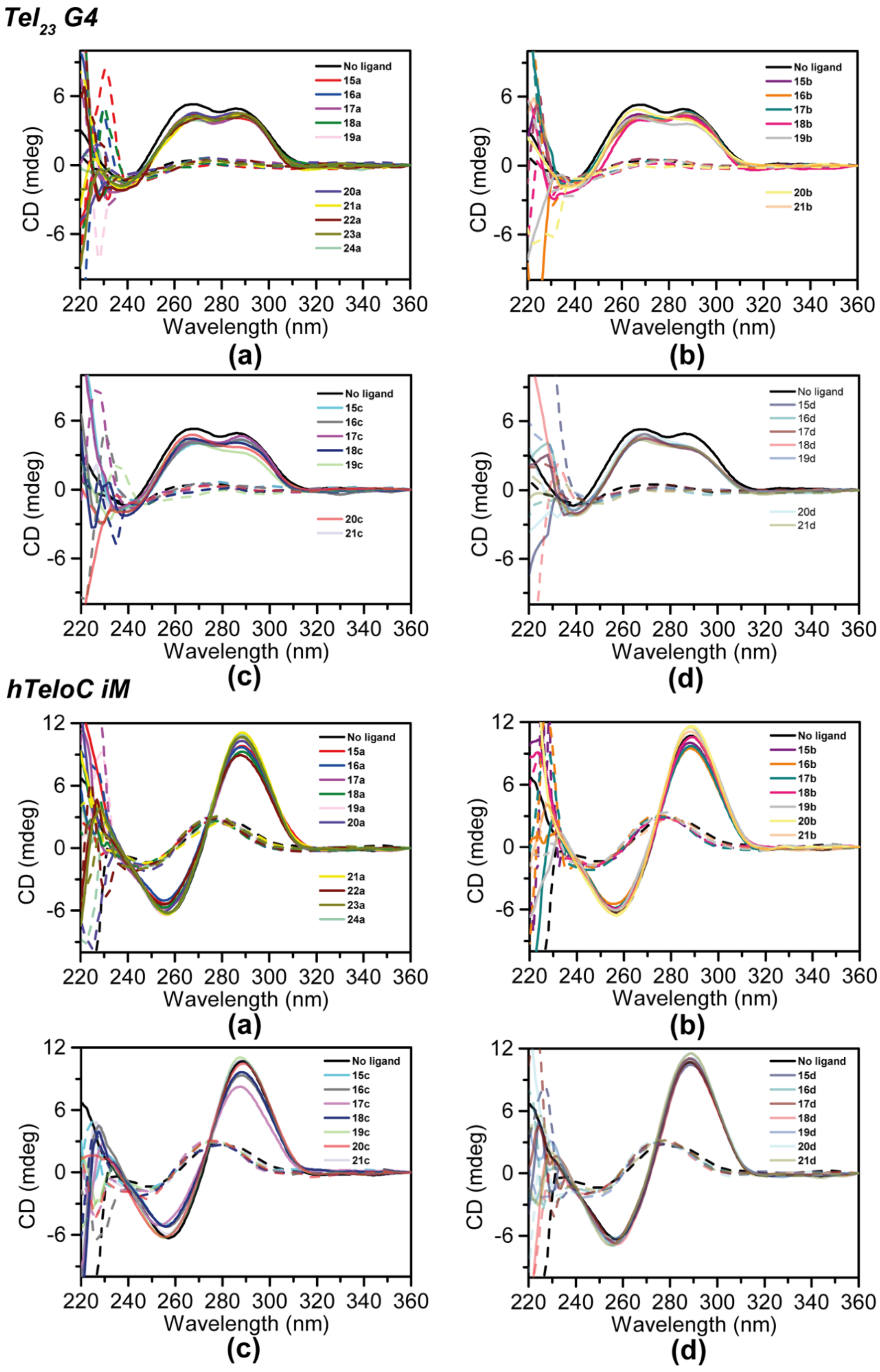

Figure S3. CD spectra of *Tel₂₃ G4* and *hTeloC iM* in the absence (black line) and presence of 10 equivalents of compounds from series a **(a)**, b **(b)**, c **(c)** and d **(d)**. The spectra were acquired at 20 °C (solid lines) and 100 °C (dashed lines) for *Tel₂₃ G4*, and at 5° (solid lines) and 90 °C (dashed lines) for *hTeloC iM*.

Hairpin

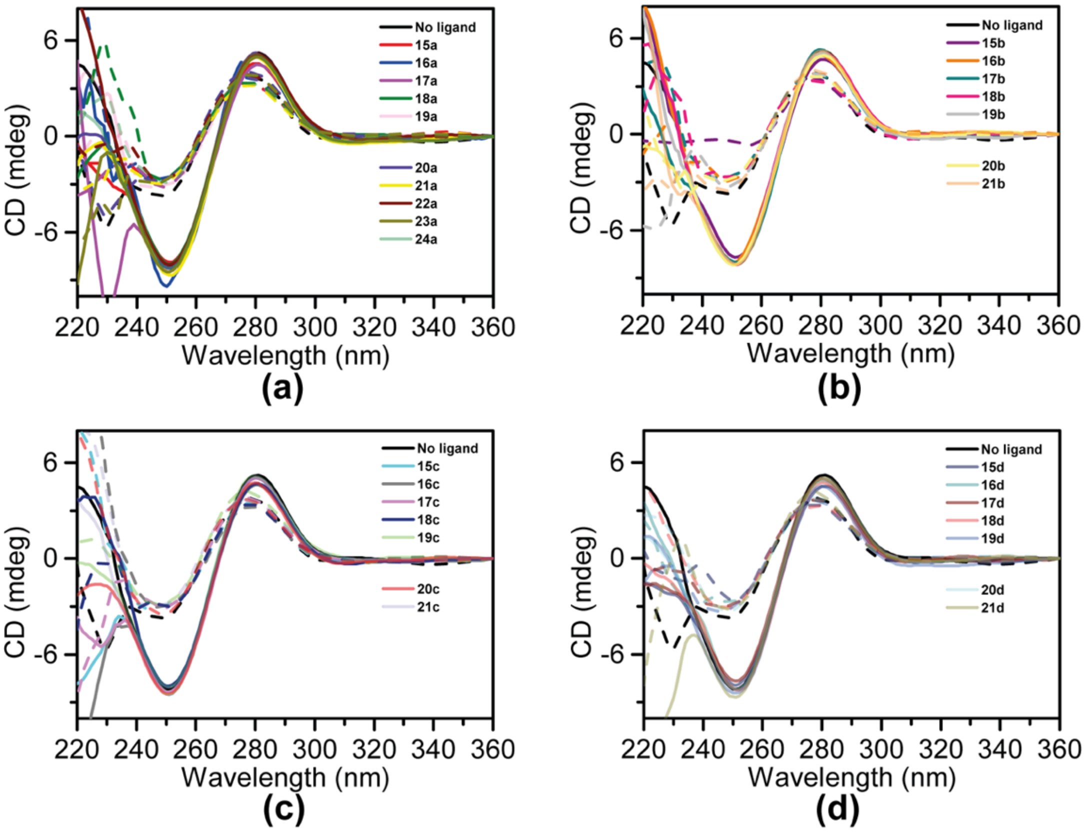

Figure S4. CD spectra of *Hairpin* in the absence (black line) and presence of 10 equivalents of compounds from series a **(a)**, b **(b)**, c **(c)** and d **(d)**. The spectra were acquired at 20 °C (solid lines) and 100 °C (dashed lines).

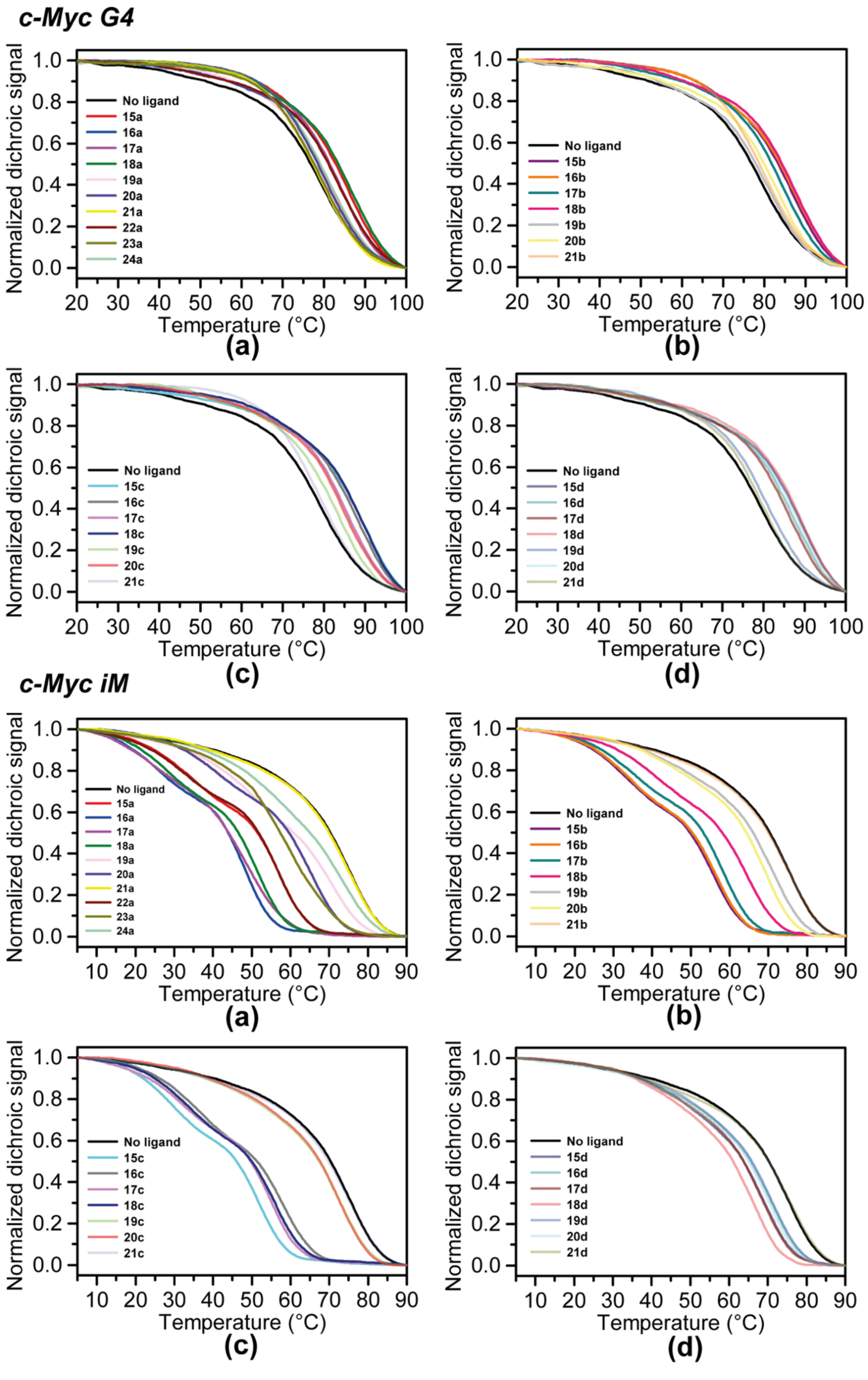

Figure S5. CD melting curves of *c-Myc G4* and *c-Myc iM* in the absence (black line) and presence of 10 equivalents of compounds from series a **(a)**, b **(b)**, c **(c)** and d **(d)**.

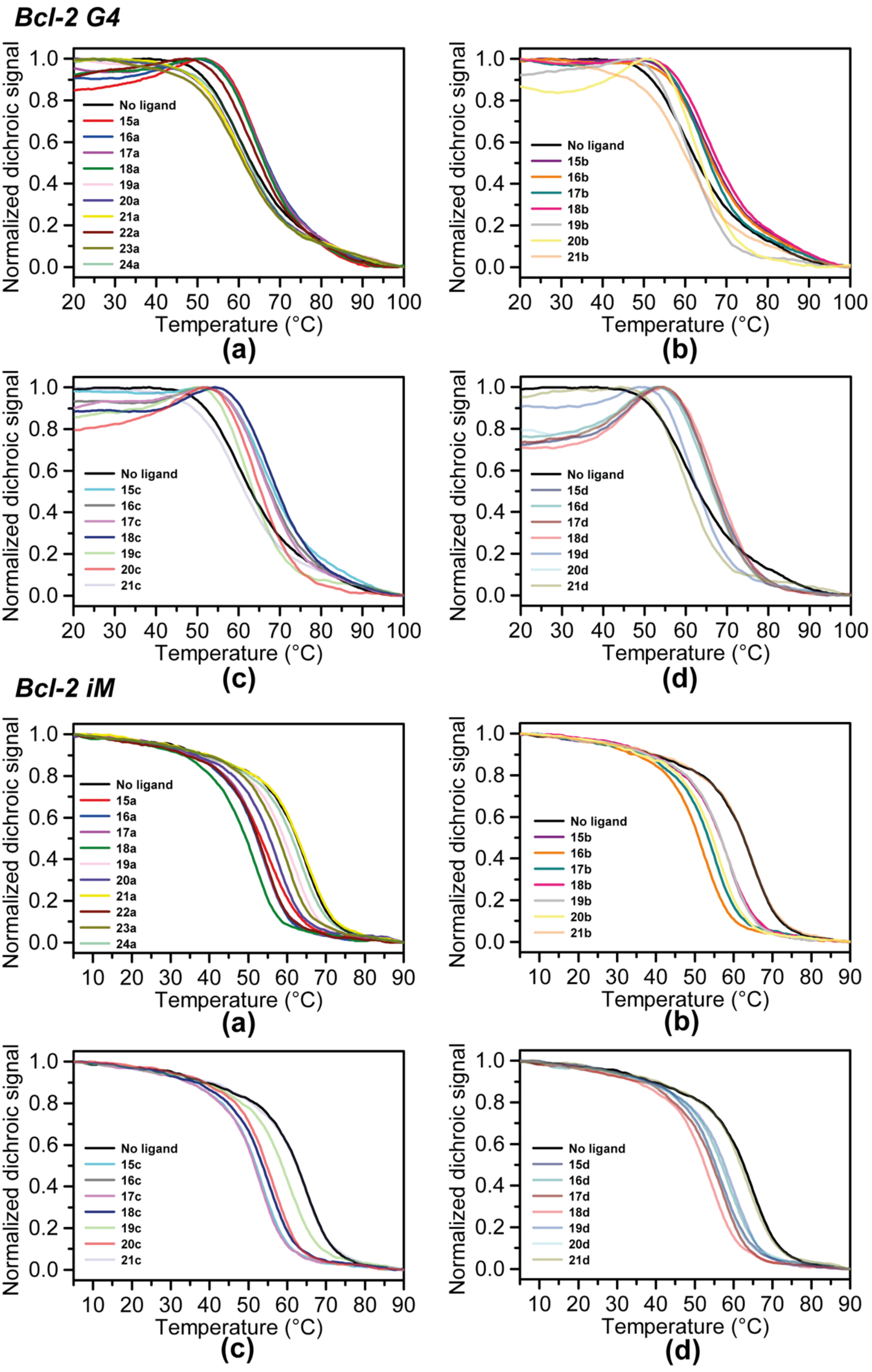

Figure S6. CD melting curves of *Bcl-2 G4* and *Bcl-2 iM* in the absence (black line) and presence of 10 equivalents of compounds from series a **(a)**, a **(b)**, c **(c)** and d **(d)**.

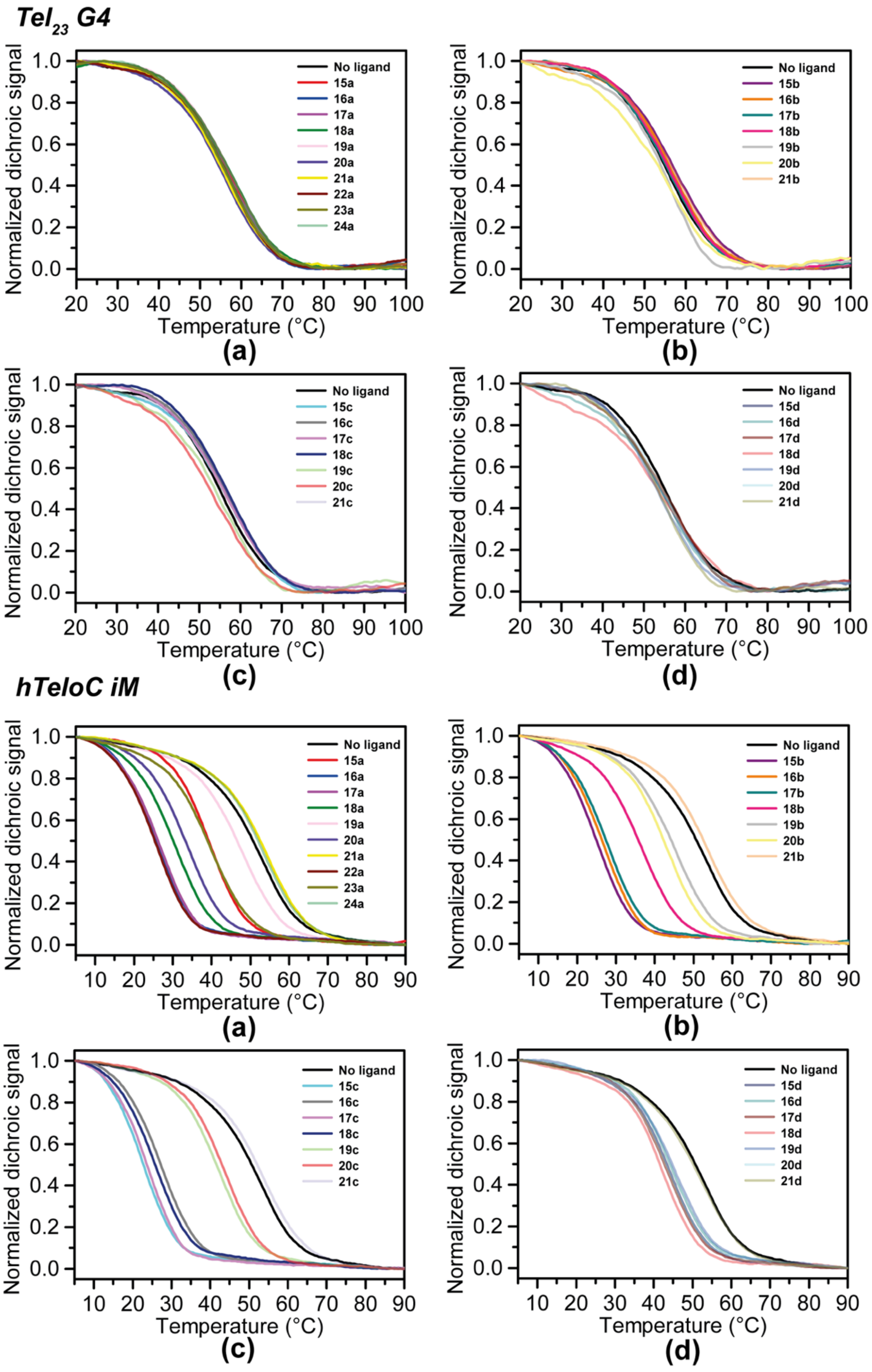

Figure S7. CD melting curves of *Tel₂₃ G4* and *hTeloC iM* in the absence (black line) and presence of 10 equivalents of compounds from series a **(a)**, b **(b)**, c **(c)** and d **(d)**.

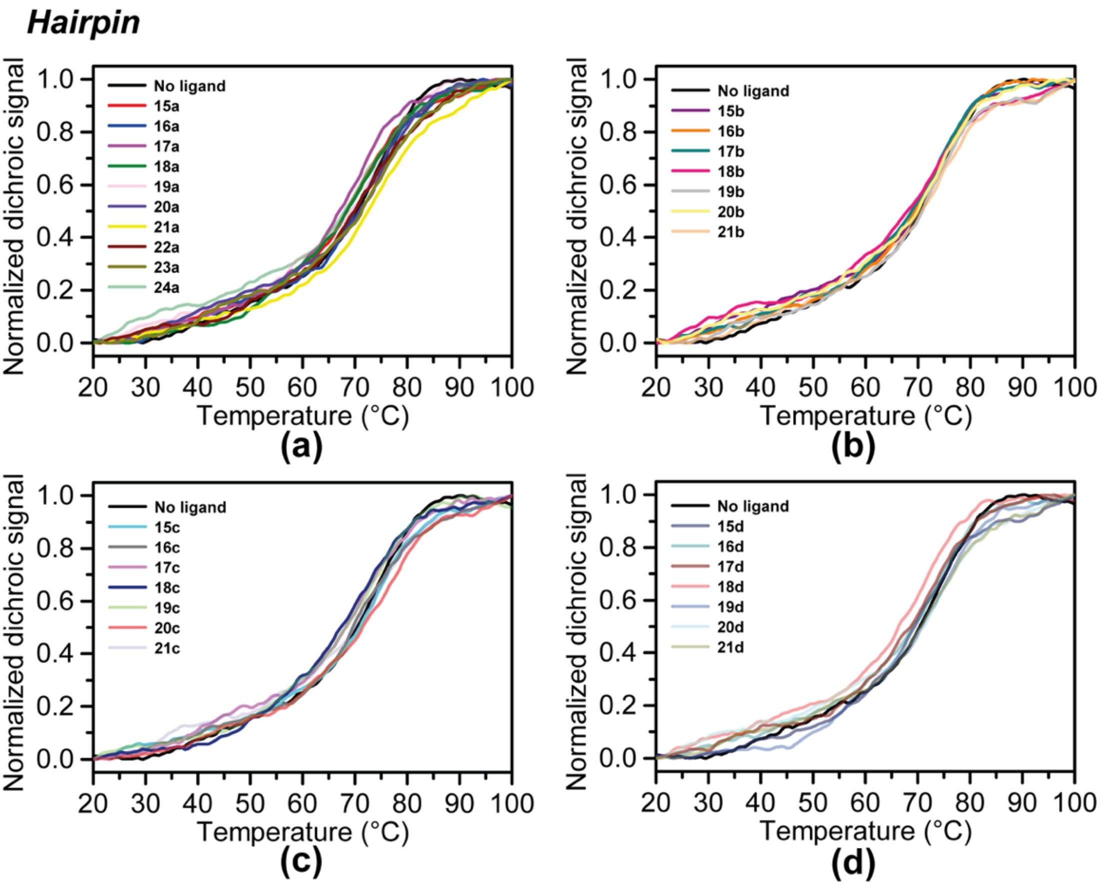

Figure S8. CD melting curves of *Hairpin* in the absence (black line) and presence of 10 equivalents of compounds from series a **(a)**, b **(b)**, c **(c)** and d **(d)**.

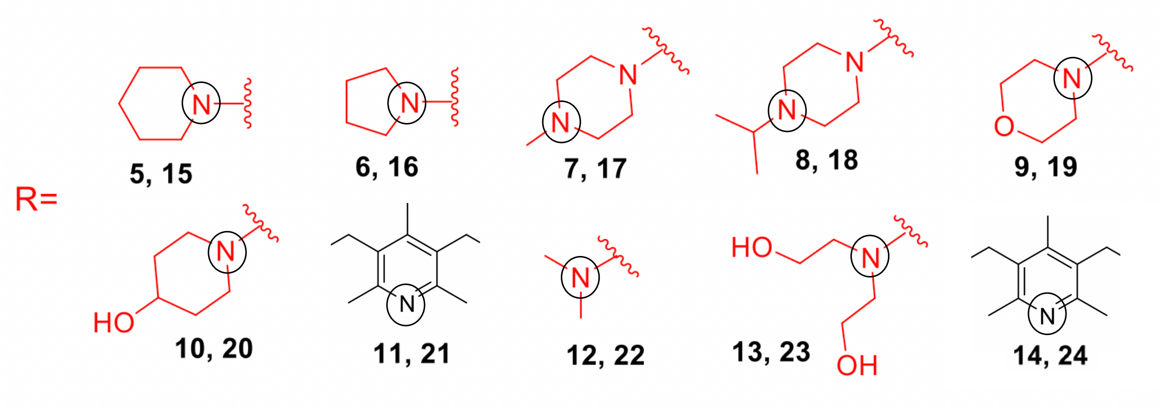

Figure S9. The strongest basic groups of the investigated compounds are circled in black. The pK_a values of their conjugated acids were used in the multivariate data analysis.

Table S1. pK_a values of the conjugated acid of the strongest base (see Figure S9) of the investigated compounds, with the corresponding pK_a interval.

Compound	pK_a value	pK_a interval
**15a**	7.5	7.1 – 8.0
**16a**	8.6	> 8.0
**17a**	7.7	7.1 – 8.0
**18a**	8.1	> 8.0
**19a**	5.8	5.1 – 6.0
**20a**	6.6	6.1 – 7.0
**21a**	5.7	5.1 – 6.0
**22a**	7.5	7.1 – 8.0
**23a**	6.1	6.1 – 7.0
**24a**	5.8	5.1 – 6.0
**15b**	9.0	> 8.0
**16b**	10.0	> 8.0
**17b**	7.9	7.1 – 8.0
**18b**	8.3	> 8.0
**19b**	6.8	6.1 – 7.0
**20b**	8.4	> 8.0
**21b**	5.7	5.1 – 6.0
**15c**	9.7	> 8.0
**16c**	10.5	> 8.0
**17c**	8.0	7.1 – 8.0
**18c**	8.4	> 8.0
**19c**	7.5	7.1 – 8.0
**20c**	8.8	> 8.0
**21c**	5.7	5.1 – 6.0
**15d**	10.0	> 8.0
**16d**	10.7	> 8.0
**17d**	8.0	7.1 – 8.0
**18d**	8.4	> 8.0
**19d**	7.7	7.1 – 8.0
**20d**	9.1	> 8.0
**21d**	5.8	5.1 – 6.0

Table S2. Differences in chemical shifts (Δδ) of selected signals of *c-Myc G4* upon addition (2 equiv) of **20a**, **23a**, **15c**, **18c**, and **21d** (G4 in the presence of compounds minus G4 alone).

Signal	Proton	δ (ppm)	Δδ (ppm)				
			20a	23a	15c	18c	21d
1	G16 (H1)	11.672	-0.02	-0.02	-0.04	-0.06	0
2	G7 (H1)	11.549	0.01	0.01	0	0	0
3	G11 (H1)	11.485	0	0	0	0	0
4	G12 (H1)	11.252	0	0	-0.01	-0.03	0
5	G21 (H1)	11.117	0	0	-0.01	-0.05	0
6	G20 (H1)	11.009	N.D.[1]	N.D.[1]	N.D.[1]	N.D.[1]	N.D.[1]
7	G8/G17 (H1)	10.919	0	0	0	-0.13	0
8	G13 (H1)	10.833	0.02	0.02	0.02	-0.02	0
9	G22 (H1)	10.808	-0.01	-0.01	-0.01	-0.03	0
10	G18 (H1)	10.794	-0.02	-0.02	-0.02	-0.01	0
11	G9 (H1)	10.384	0.02	0.02	-0.01	-0.10	0
12	A15 (H8)	8.364	0.03	0.03	0.04	0.05	0
13	A15 (H2)	8.234	0.06	0.07	0.09	0.12	0.01
14	G16 (H8)	7.894	0	0	-0.01	-0.03	0
15	G7 (H8)	7.875	0.03	0.04	0.05	0.02	0
16	G5 (H8)	7.827	0.01	0.01	0	-0.02	0
17	G11 (H8)	7.774	0.01	0.01	0.01	0	0
18	G12/G20 (H8)	7.684	0.01	0.01	0	0	0
19	G21 (H8)	7.664	0.01	0.01	0.01	0.01	0
20	G13 (H8)/T19 (H6)	7.651	N.D.[1]	N.D.[1]	N.D.[1]	N.D.[1]	N.D.[1]
21	T10 (H6)	7.637	0	0	0	0	0
22	G17/G18 (H8)	7.632	0	0	0	0	0
23	A6 (H2)	7.615	N.D.[1]	N.D.[1]	N.D.[1]	N.D.[1]	N.D.[1]
24	A24 (H8)	7.580	N.D.[1]	N.D.[1]	-0.01	-0.02	0
25	G9 (H8)	7.553	0	0	-0.01	-0.02	0
26	G8 (H8)	7.532	0	0	-0.01	-0.02	0
27	T14 (H6)	7.425	0.01	0.01	0.01	0.01	0
28	A6 (H8)	7.418	0	0	0	0.01	0
29	G22 (H8)	7.377	-0.01	-0.02	-0.03	-0.03	0
30	A25 (H8)	7.327	0.03	0.02	-0.01	N.D. [1]	0.01
31	A25 (H2)	7.254	0.06	0.06	0.05	-0.01	0.01
32	T4 (H6)	7.008	0.01	0.01	0	-0.02	N.D.[1]
33	T23 (H6)	6.961	N.D.[1]	N.D.[1]	N.D.[1]	N.D.[1]	N.D.[1]
34	A24 (H2)	6.906	N.D.[1]	N.D.[1]	N.D.[1]	N.D.[1]	N.D.[1]

[1] N.D. = Not determined.

Table S3. Differences in chemical shifts (Δδ) of selected signals of *hTeloC iM* upon addition (2 equiv) of **20a**, **23a**, **15c**, **18c**, and **21d** (iM in the presence of compounds minus iM alone).

Signal	Proton	δ (ppm)	Δδ (ppm)				
			**20a**	**23a**	**15c**	**18c**	**21d**
1	C7:C19$^+$/C8:C20$^+$	15.498	0	0	0	0	0
2	C3$^+$:C15/C2$^+$:C14	15.377	0	0	0	0	0
3	C9:C21$^+$/C1$^+$:C13	15.212	0	0	0	0	0
4	A6/A11/A12 (H8)	8.209	0.02	0.02	0.03	0.03	0
5	A6/A12 (H2)	8.130	0.02	0.02	0.02	0.02	0
6	A11 (H2)/A17 (H2, H8)	7.974	0.04	0.04	0.06	0.07	0
7	T22 (H6)	7.748	N.D.[1]	N.D.[1]	N.D.[1]	N.D.[1]	0
8	A5 (H2)	7.738	0.03	0.03	0.03	0.04	0
9	T10 (H6)	7.531	0	0	0	0	0
10	A18 (H2)	7.408	0.02	0.02	0.02	0.03	0
11	A5 (H8)	7.398	0.01	0.01	0.02	0.02	0
12	T16 (H6)	7.354	0.01	0.01	0.01	0.01	0
13	A18 (H8)	7.206	0.01	0.01	0.01	0.01	0

[1] N.D. = Not determined.

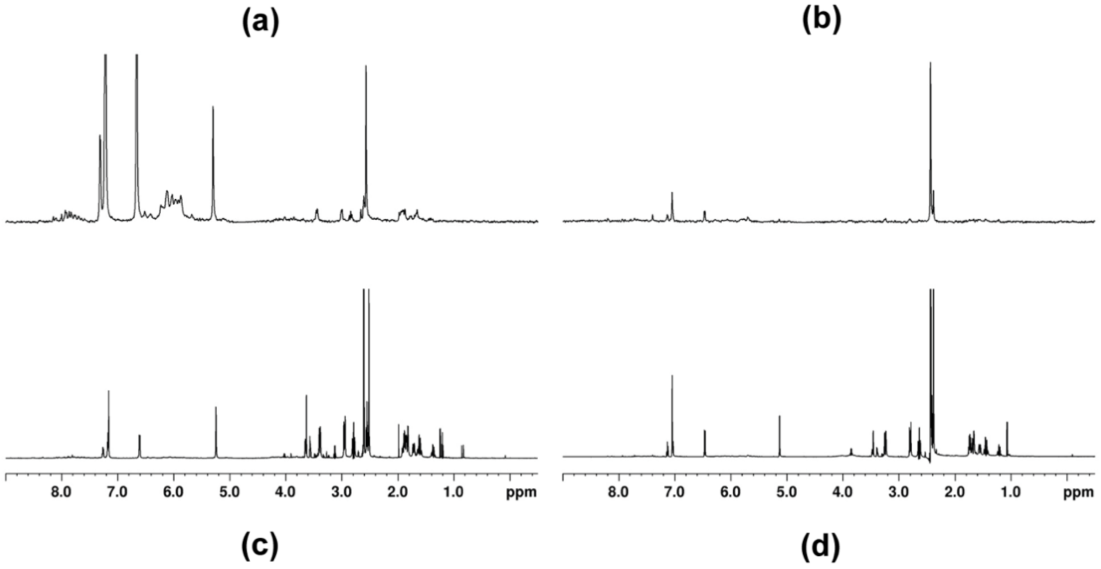

Figure S10. 1D ^{1}H NMR spectra (**c** and **d**) and STD spectra (**a** and **b)** of compound **15c** (0.250 mM) in presence of *c-Myc G4* (**a** and **c**) and *hTeloC iM* (**b** and **d**).

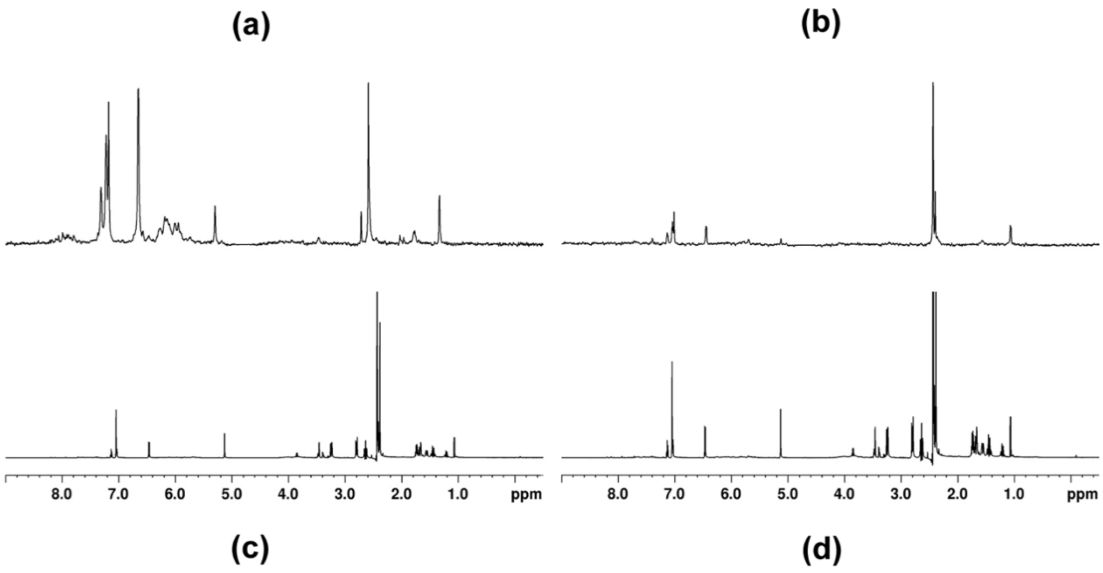

Figure S11. 1D ^{1}H NMR spectra (**c** and **d**) and STD spectra (**a** and **b**) of compound **18c** (0.250 mM) in presence of *c-Myc G4* (**a** and **c**) and *hTeloC iM* (**b** and **d**).

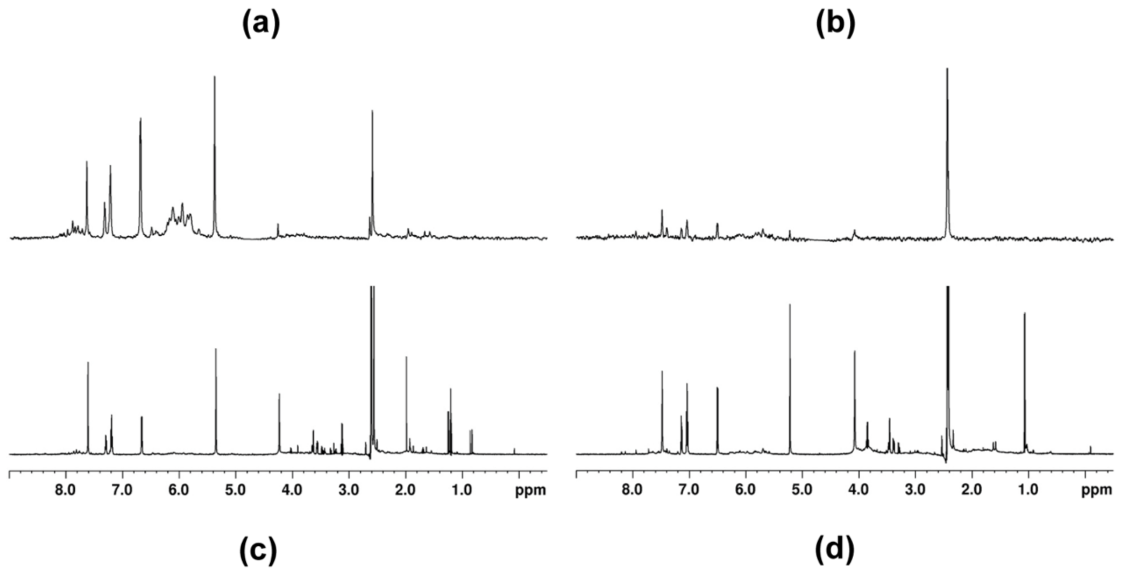

Figure S12. 1D ^{1}H NMR spectra (**c** and **d**) and STD spectra (**a** and **b**) of compound **20a** (0.250 mM) in presence of *c-Myc G4* (**a** and **c**) and *hTeloC iM* (**b** and **d**).

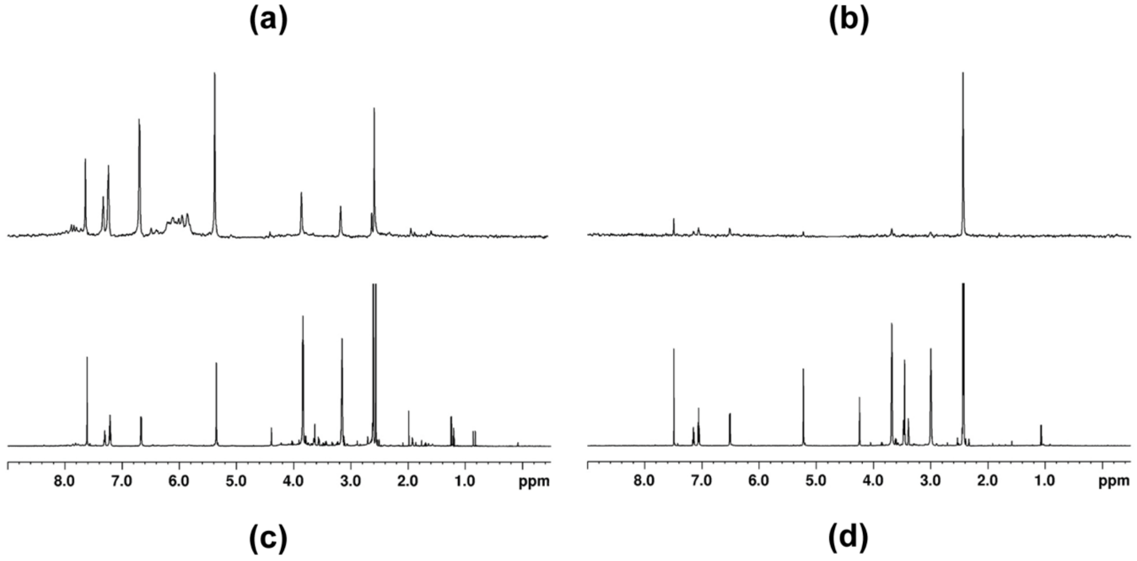

Figure S13. 1D ^{1}H NMR spectra (**c** and **d**) and STD spectra (**a** and **b**) of compound **23a** (0.250 mM) in presence of *c-Myc G4* (**a** and **c**) and *hTeloC iM* (**b** and **d**).

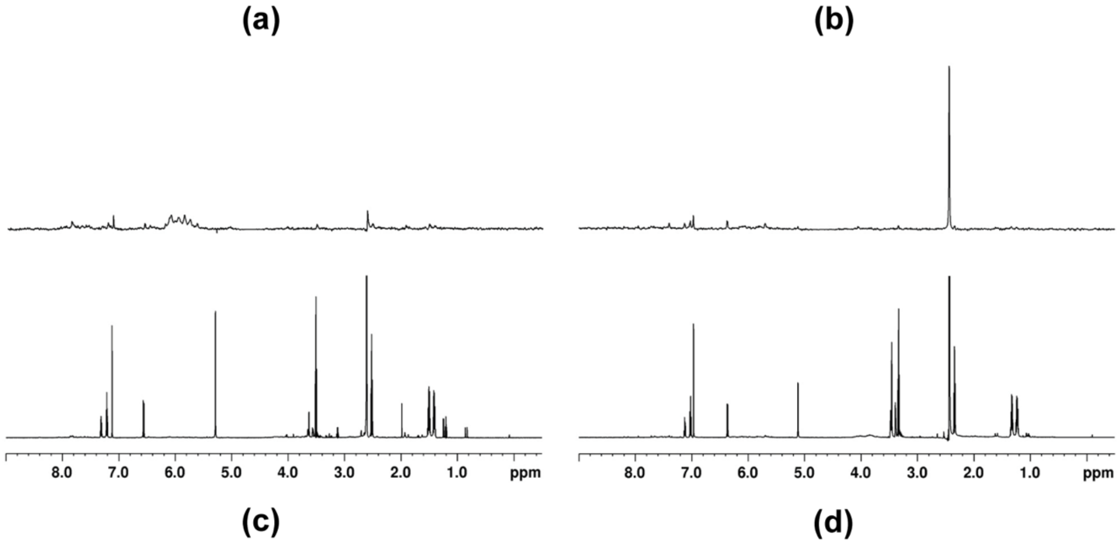

Figure S14. 1D ^{1}H NMR spectra (**c** and **d**) and STD spectra (**a** and **b**) of compound **21d** (0.250 mM) in presence of *c-Myc G4* (**a** and **c**) and *hTeloC iM* (**b** and **d**).

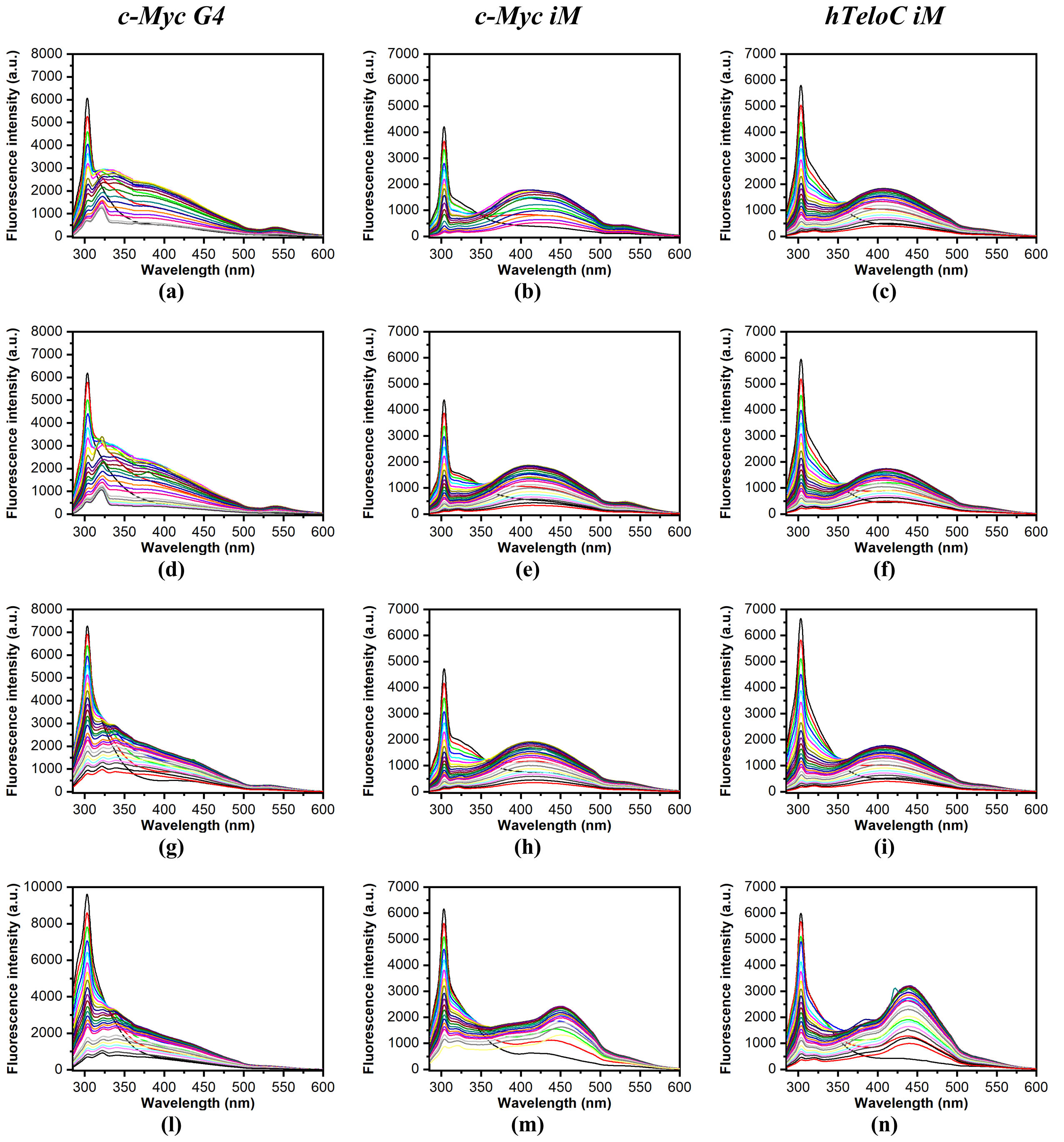

Figure S15. Representative fluorescence emission spectra of (**a**,**b**,**c**) **20a**, (**d**,**e**,**f**) **23a**, (**g**,**h**,**i**) **15c**, and (**l**,**m**,**n**) **18c** (2.5–3.5 µM) in the absence and presence of stepwise additions (5 µL) of *c-Myc G4*, *c-Myc iM*, or *hTeloC iM* (150–200 µM), at 25 °C.

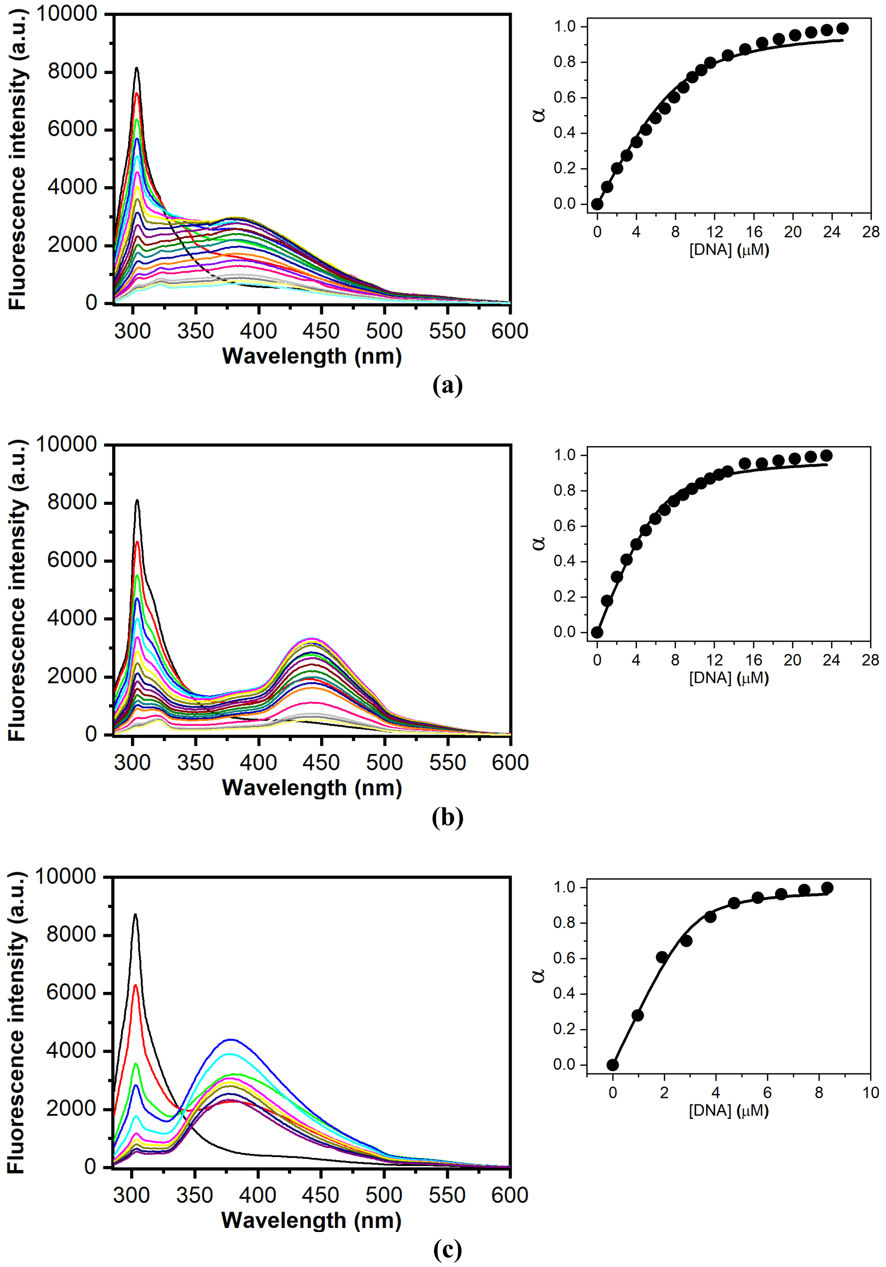

Figure S16. Representative fluorescence emission spectra of (**a**,**b**) **21d**, and (**c**) **18c** (2.5–3.5 μM) in the absence and presence of stepwise additions (5 μL) of (**a**) *c-Myc G4*, (**b**) *c-Myc iM*, or (**c**) single-stranded DNA d(CT)$_{15}$ (150–200 μM), at 25 °C. Insets show the titration curves obtained by plotting the fraction of bound compound (*α*) versus DNA concentration.

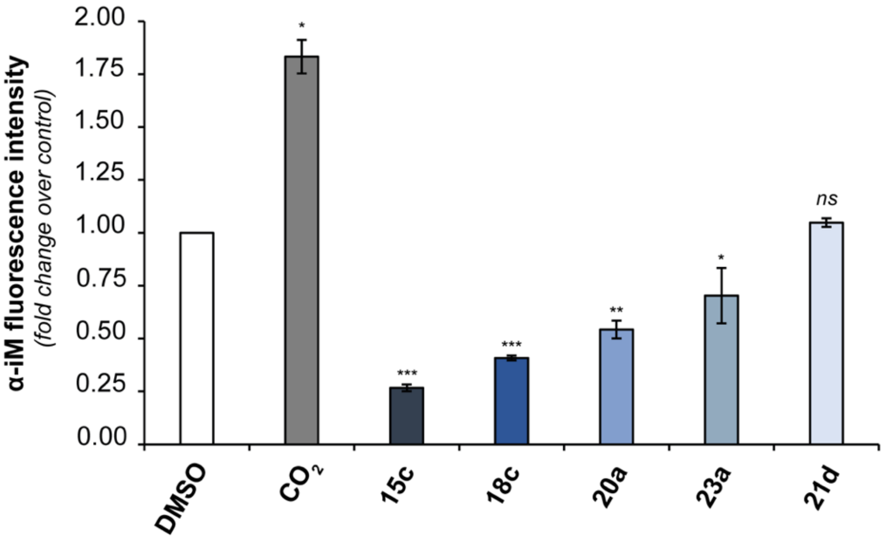

(a)

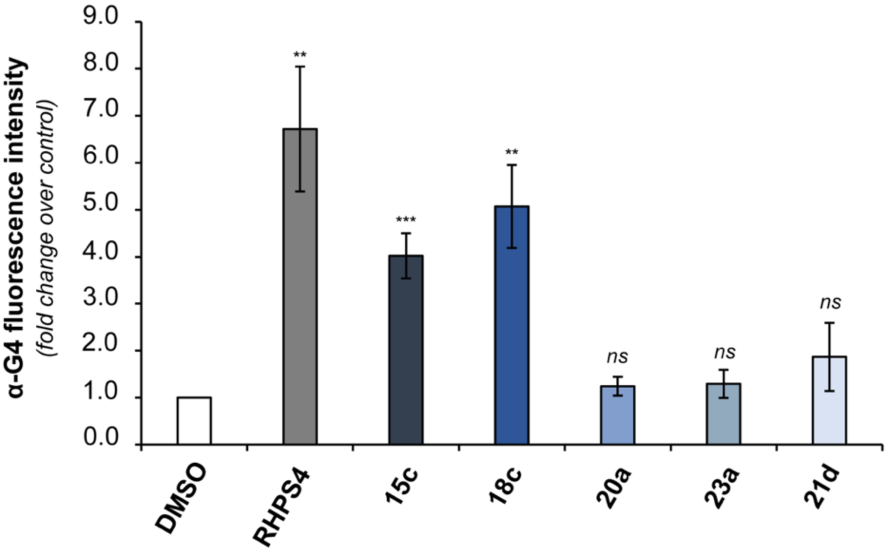

(b)

Figure S17. Quantitative analysis of fluorescence intensity of anti-iM (**a**) and anti-G4 (**b**) signal. For each panel, 25 cells/condition were analysed by ImageJ software and the results were expressed as fold change of fluorescence intensity over the negative control (DMSO). Results are referred to the images shown in Figures 6 and 7, respectively. Histograms show the mean ± SD of three independent experiments. Statistical significance was calculated using unpaired student *t*-tests on GraphPad Prism 6 (****p*<0.001; ***p*<0.01; **p*<0.05).

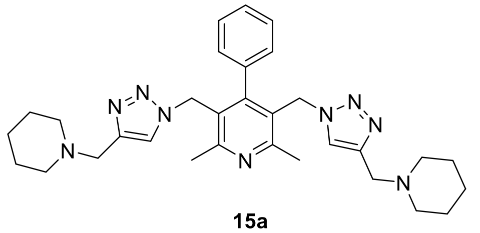

15a

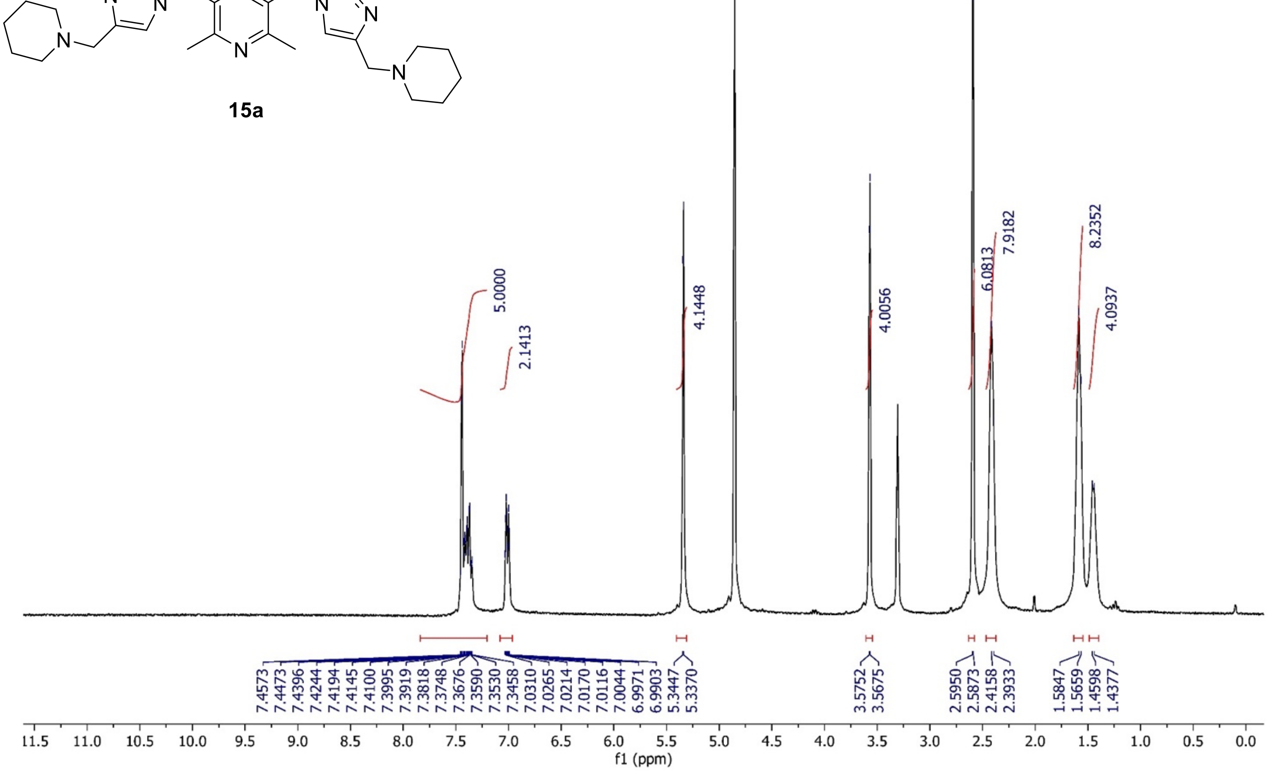

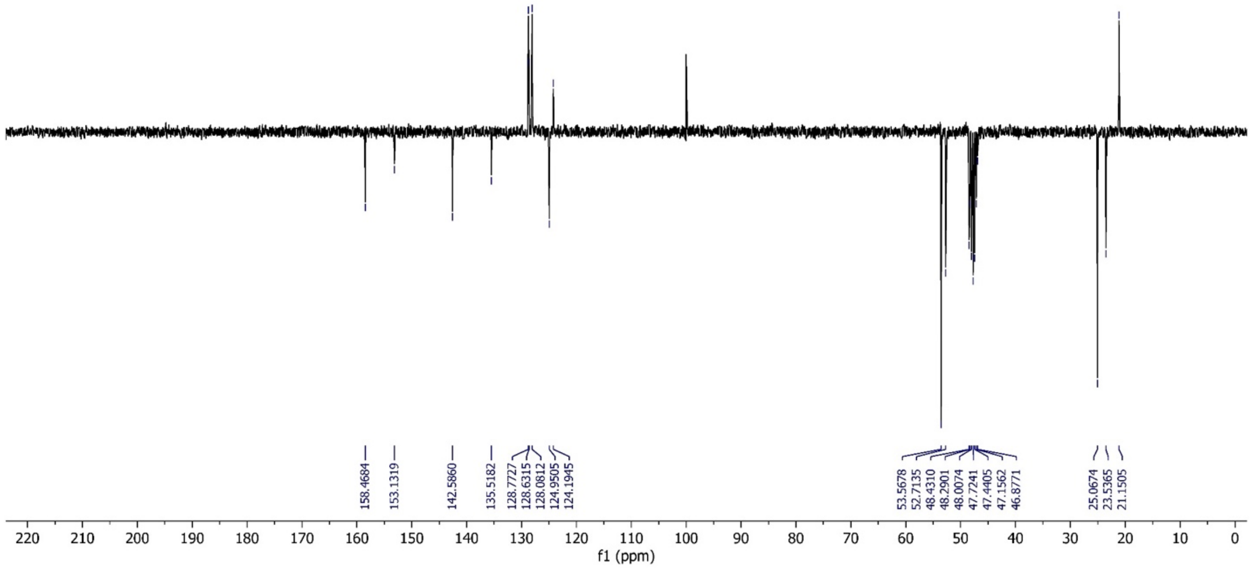

Figure S18. 1D ^{1}H (*top*) and ^{13}C APT (*bottom*) NMR spectra of compound **15a**.

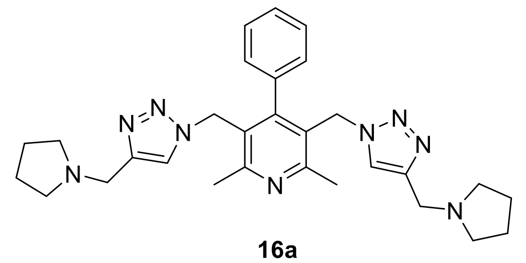

16a

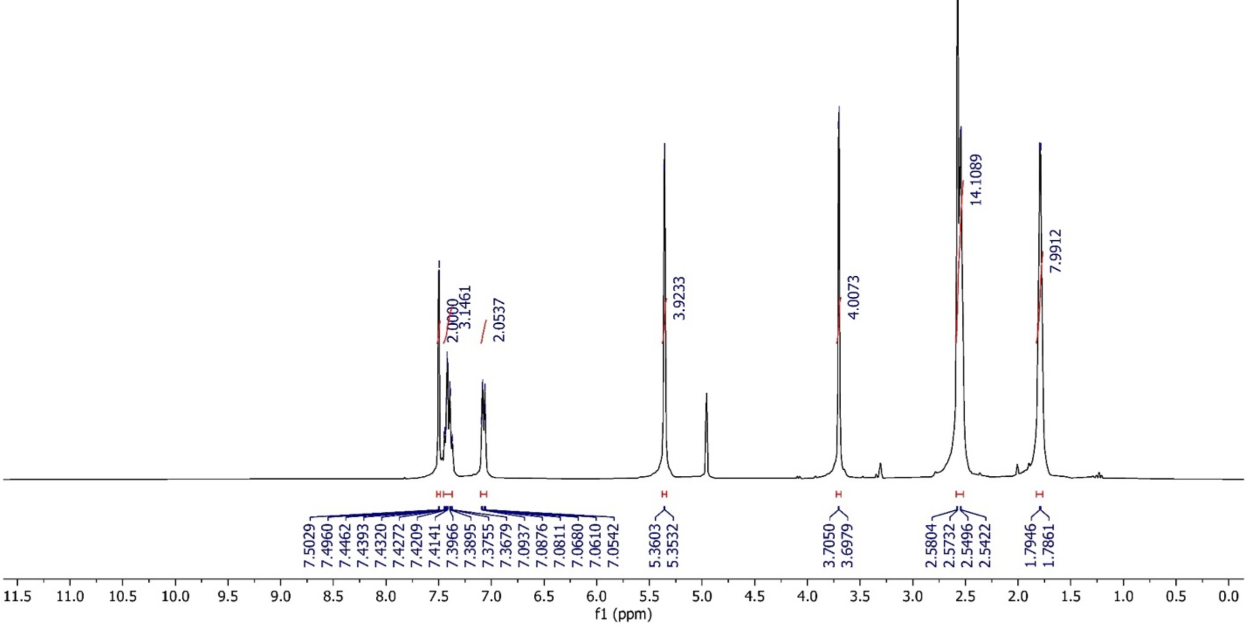

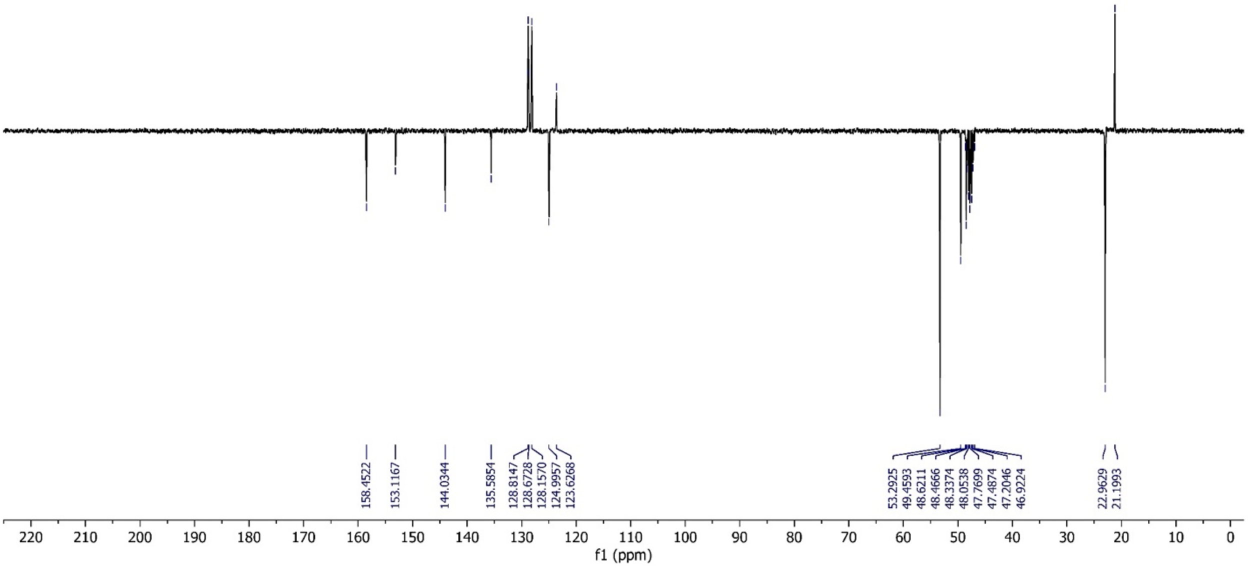

Figure S19. 1D ^{1}H (*top*) and ^{13}C APT (*bottom*) NMR spectra of compound **16a**.

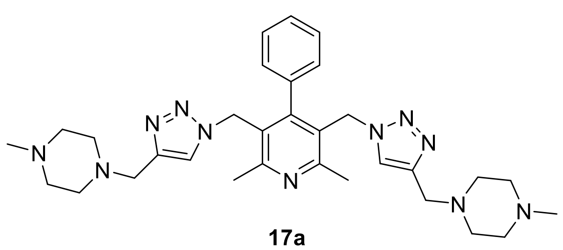

17a

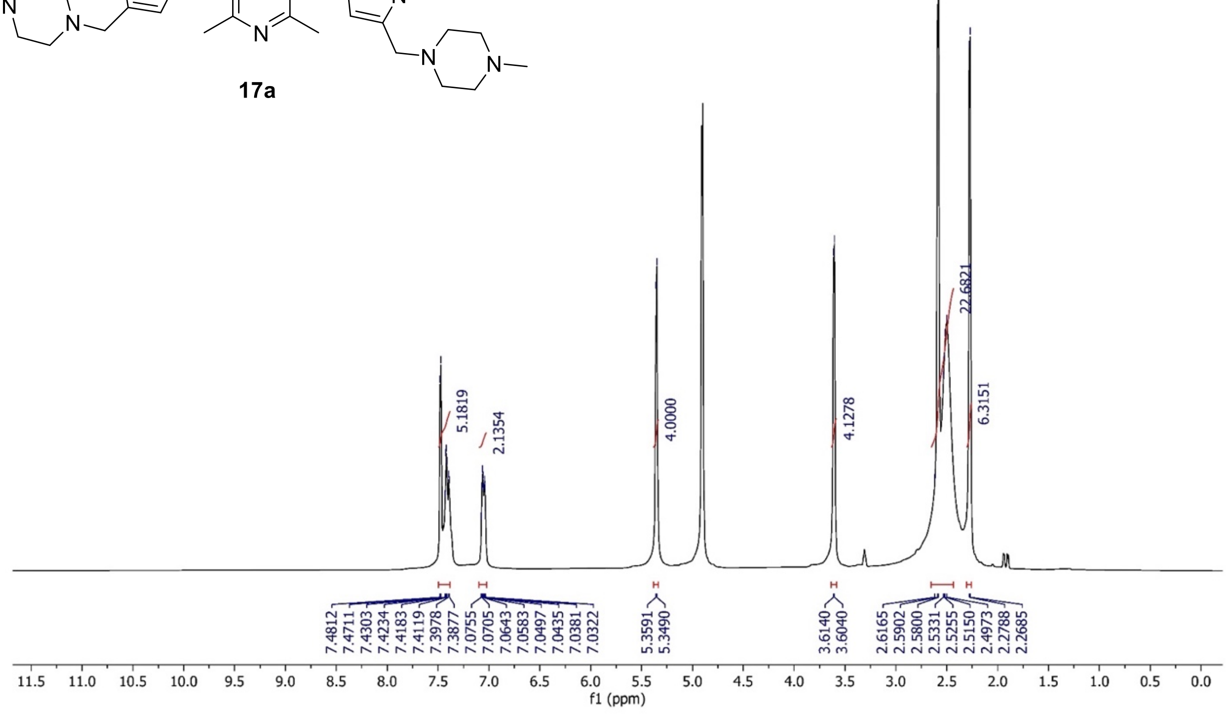

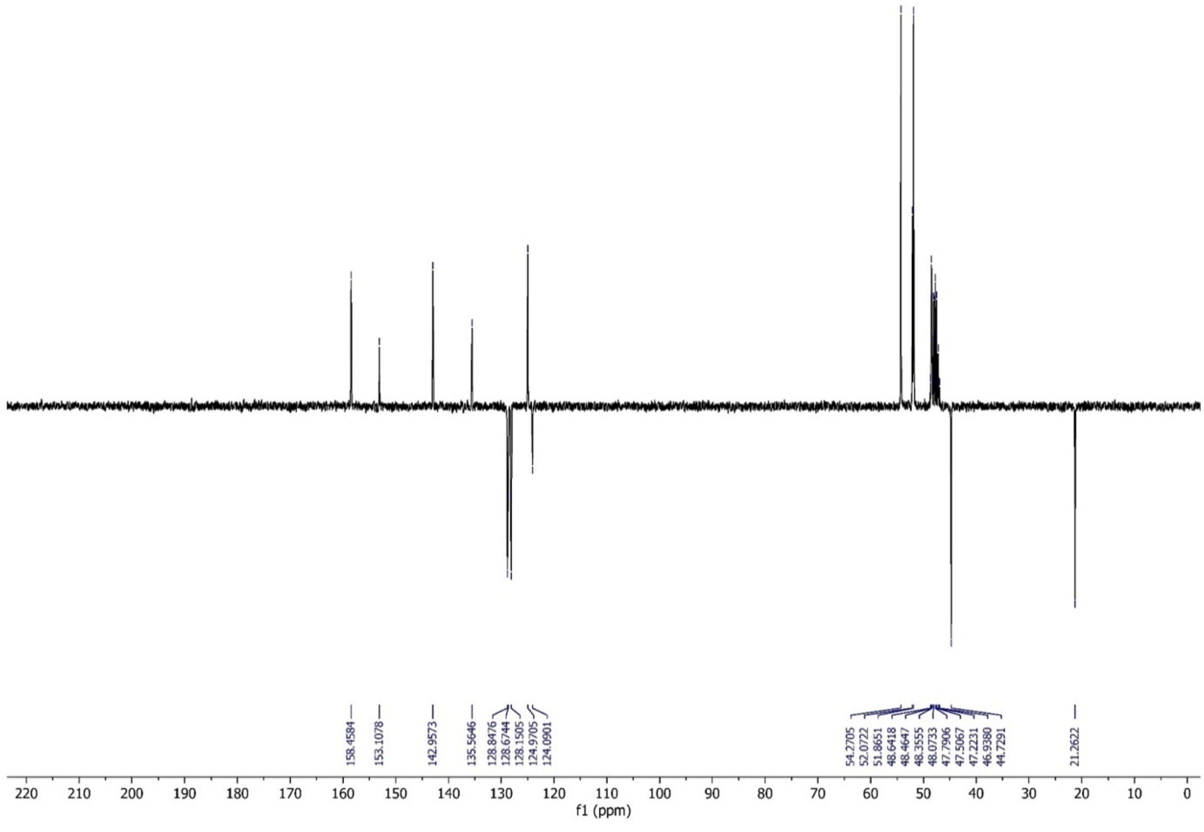

Figure S20. 1D ^{1}H (*top*) and ^{13}C APT (*bottom*) NMR spectra of compound **17a**.

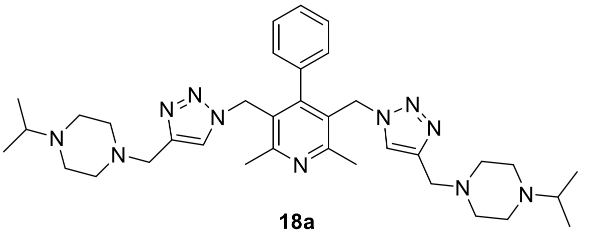

18a

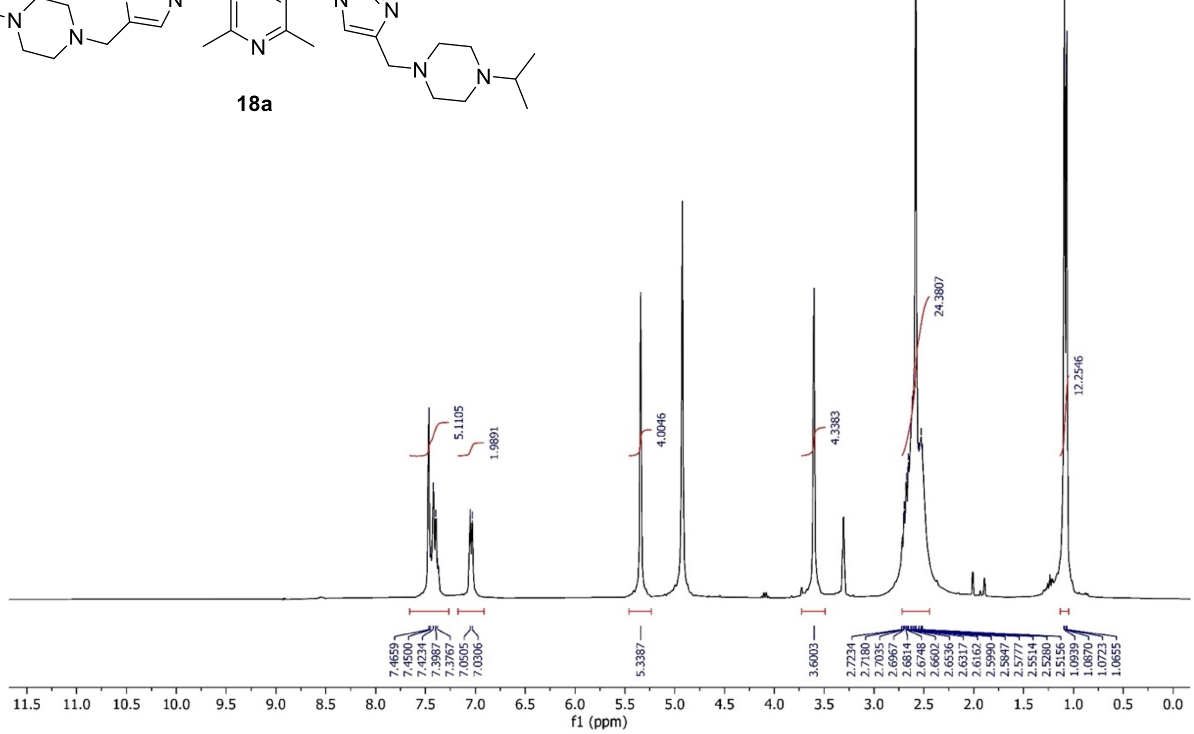

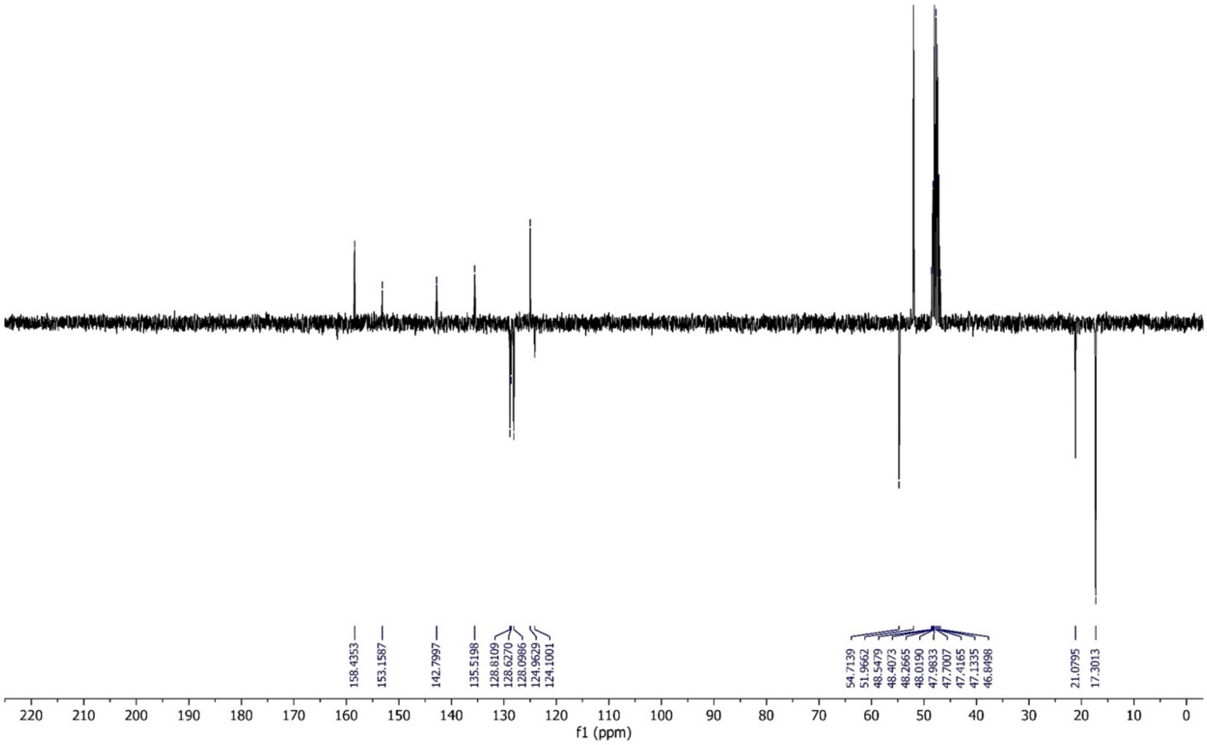

Figure S21. 1D ^{1}H (*top*) and ^{13}C APT (*bottom*) NMR spectra of compound **18a**.

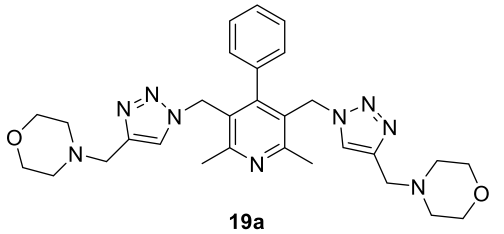

19a

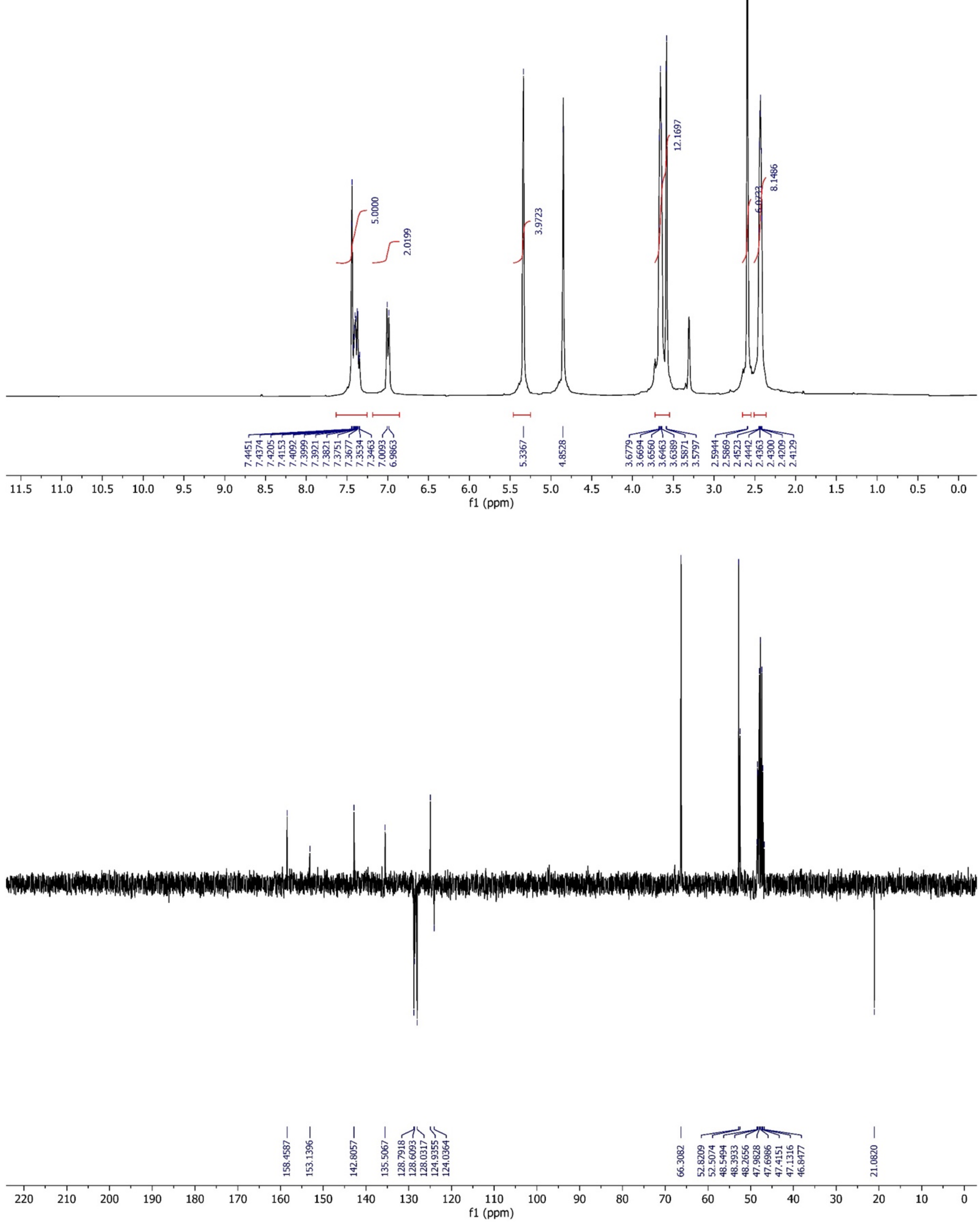

Figure S22. 1D ^{1}H (*top*) and ^{13}C APT (*bottom*) NMR spectra of compound **19a.**

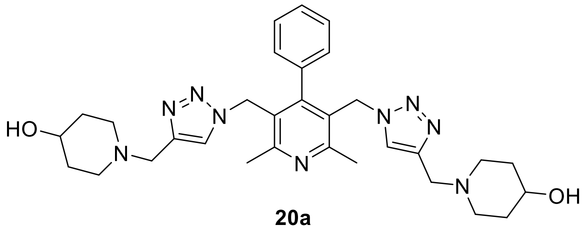

20a

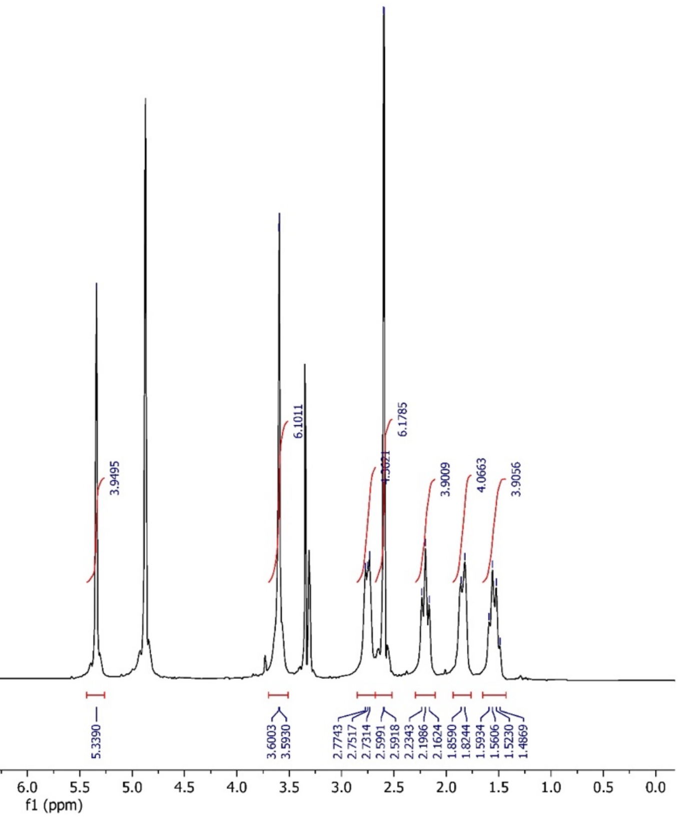

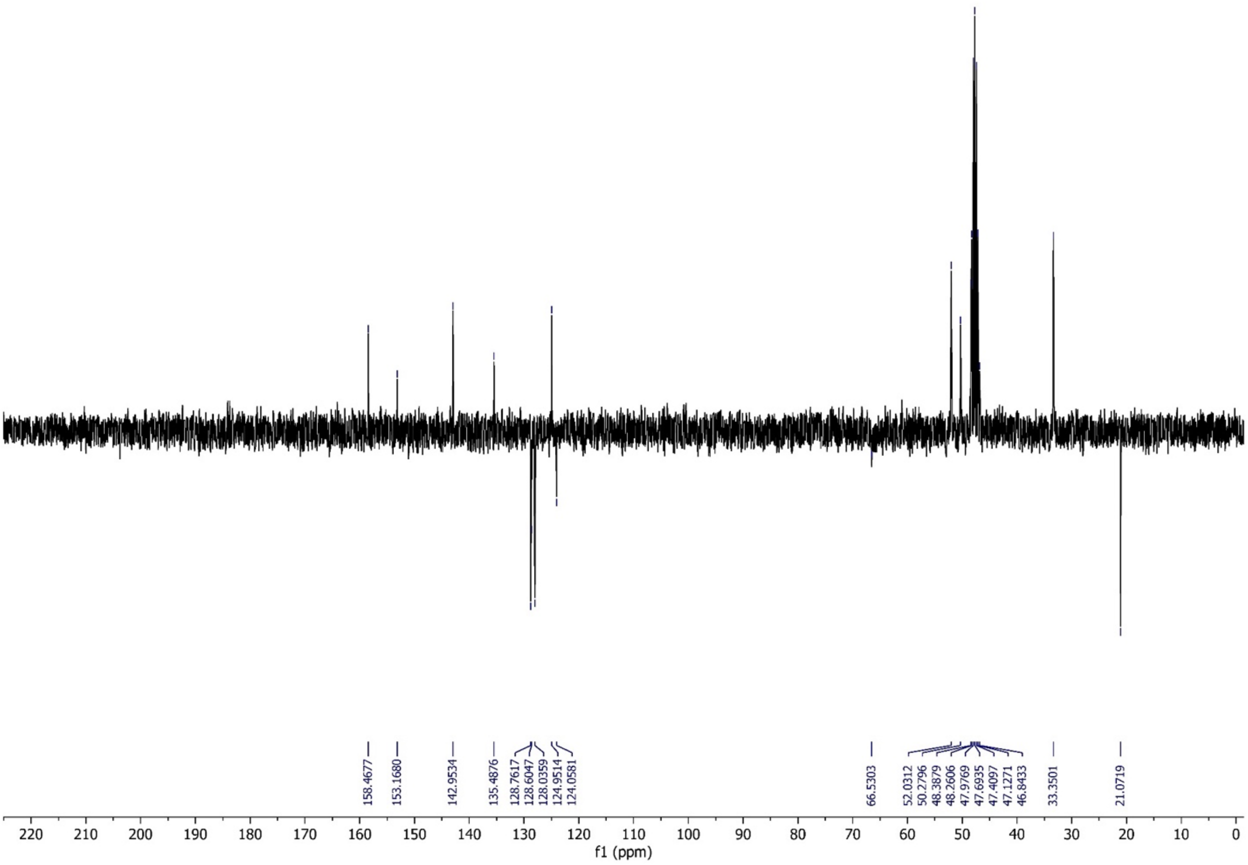

Figure S23. 1D ^{1}H (*top*) and ^{13}C APT (*bottom*) NMR spectra of compound **20a**.

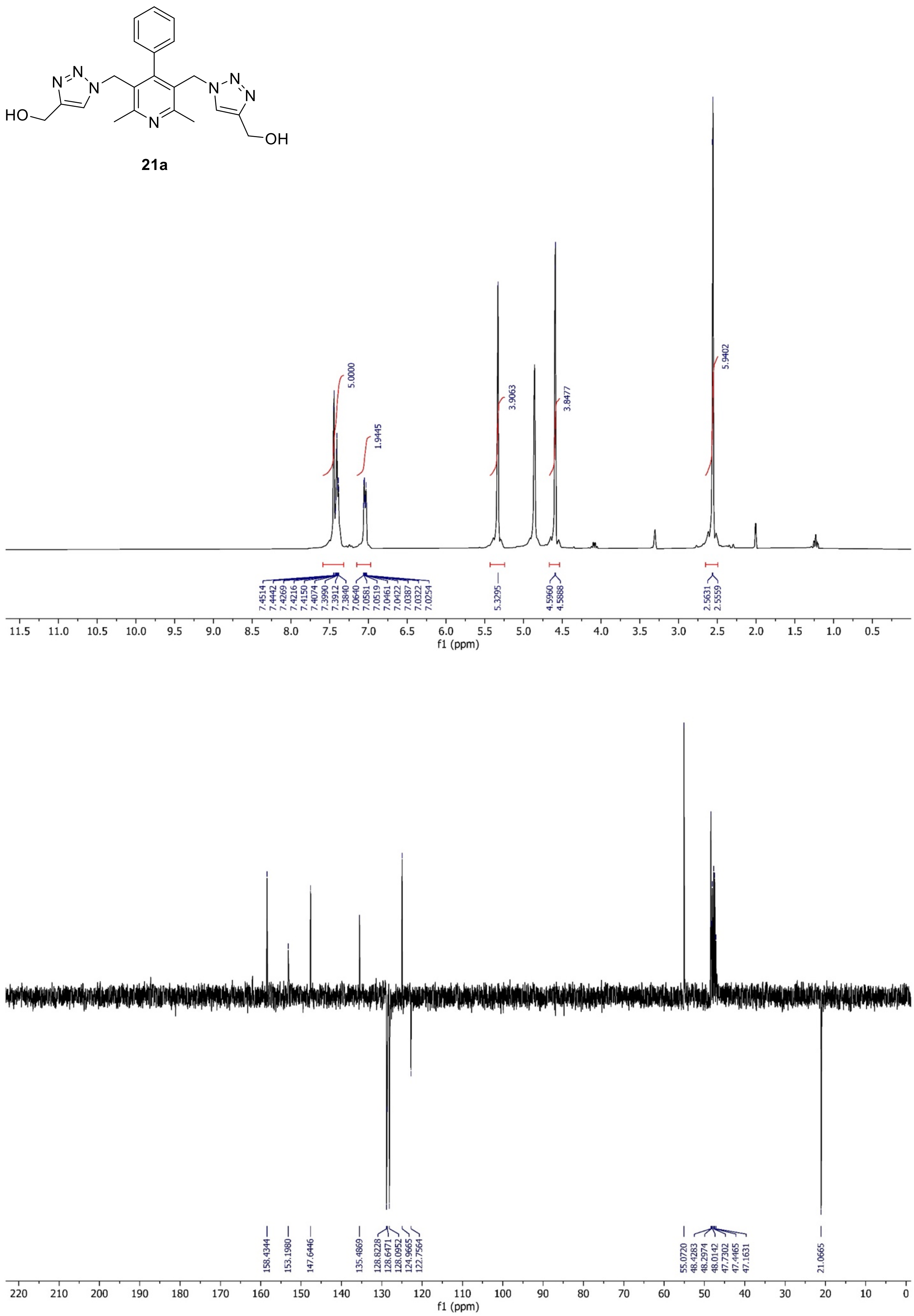

Figure S24. 1D ^{1}H (*top*) and ^{13}C APT (*bottom*) NMR spectra of compound **21a**.

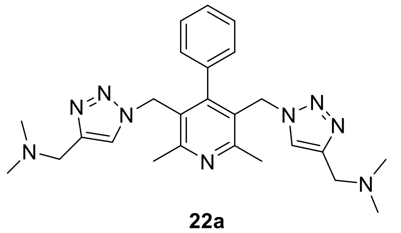

22a

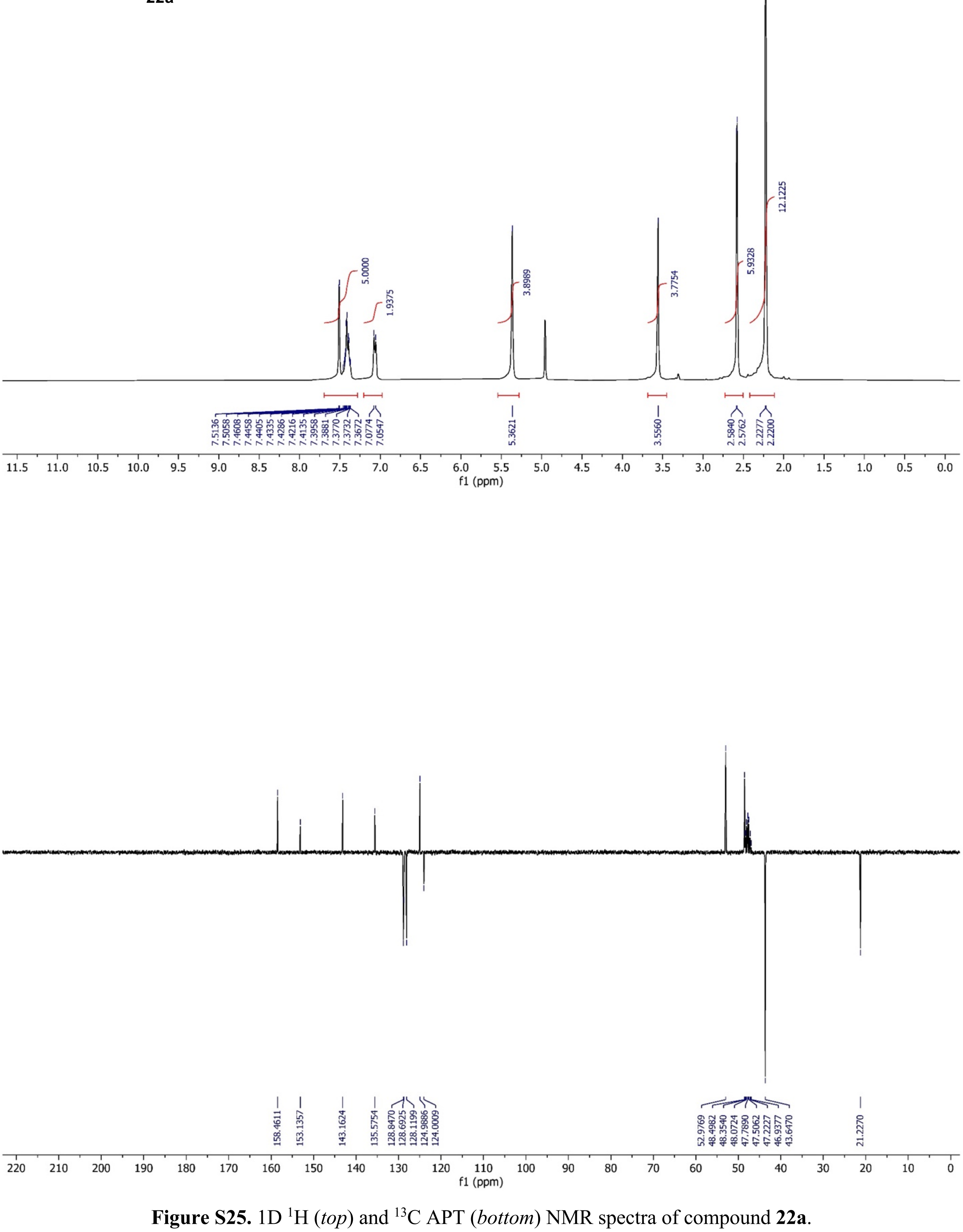

Figure S25. 1D ^{1}H (*top*) and ^{13}C APT (*bottom*) NMR spectra of compound **22a**.

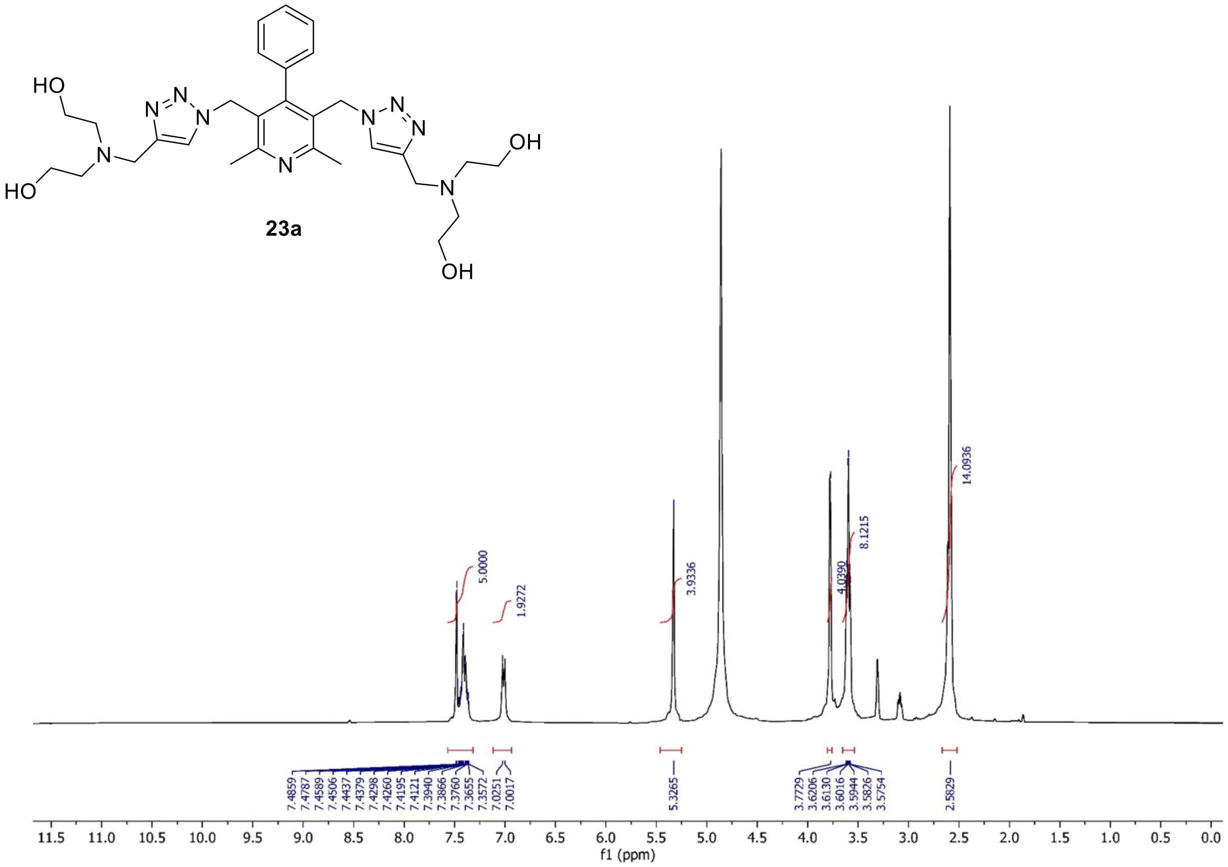

23a

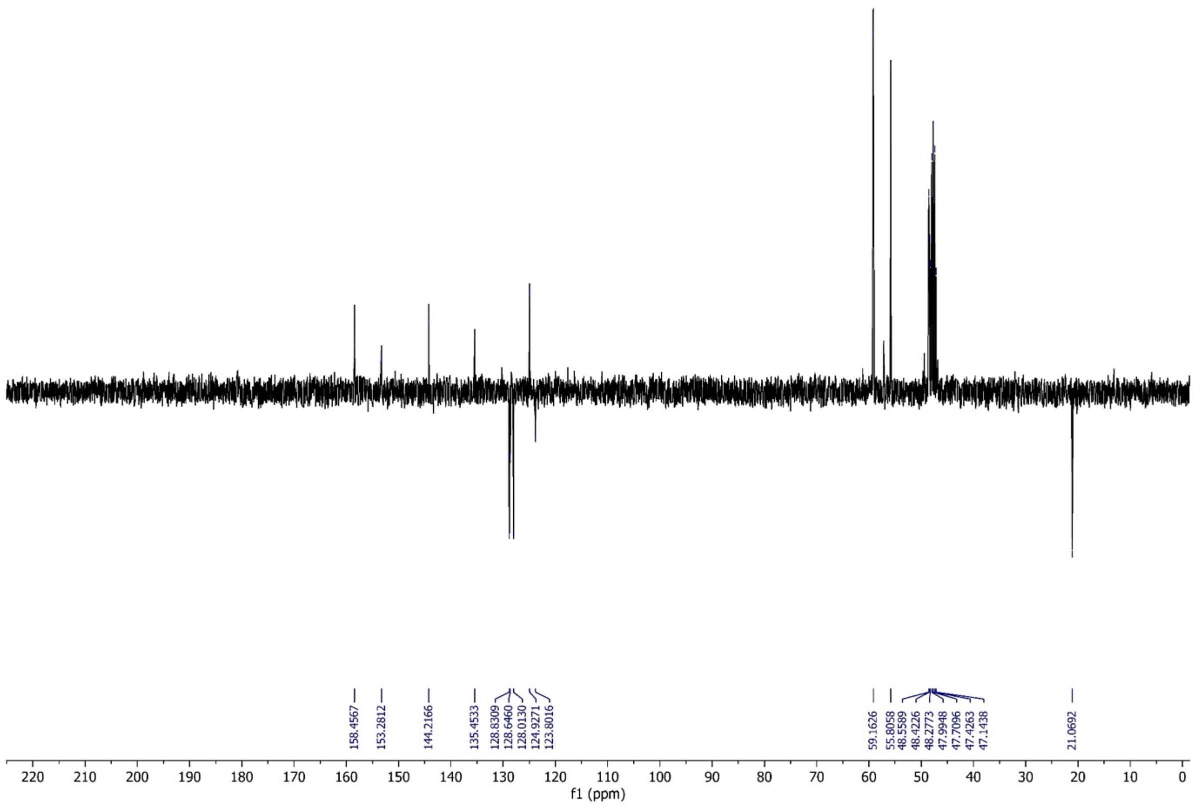

Figure S26. 1D ^{1}H (*top*) and ^{13}C APT (*bottom*) NMR spectra of compound **23a**.

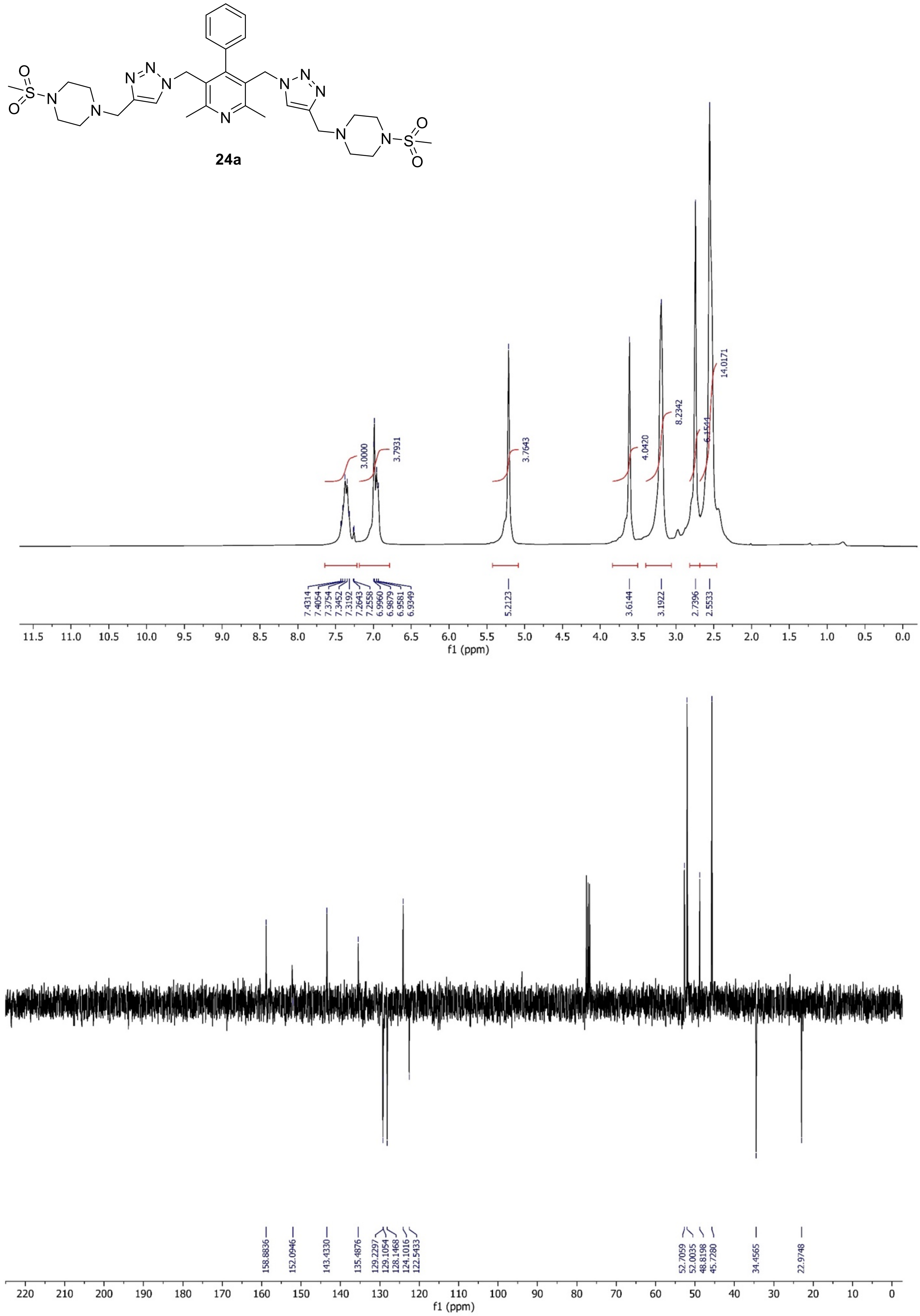

Figure S27. 1D ^{1}H (*top*) and ^{13}C APT (*bottom*) NMR spectra of compound **24a**.

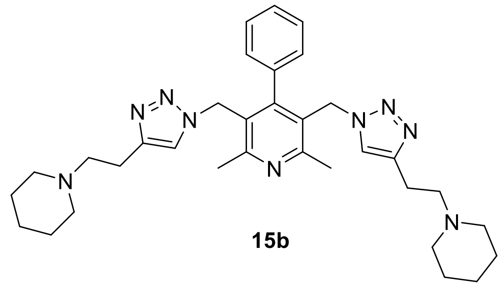

15b

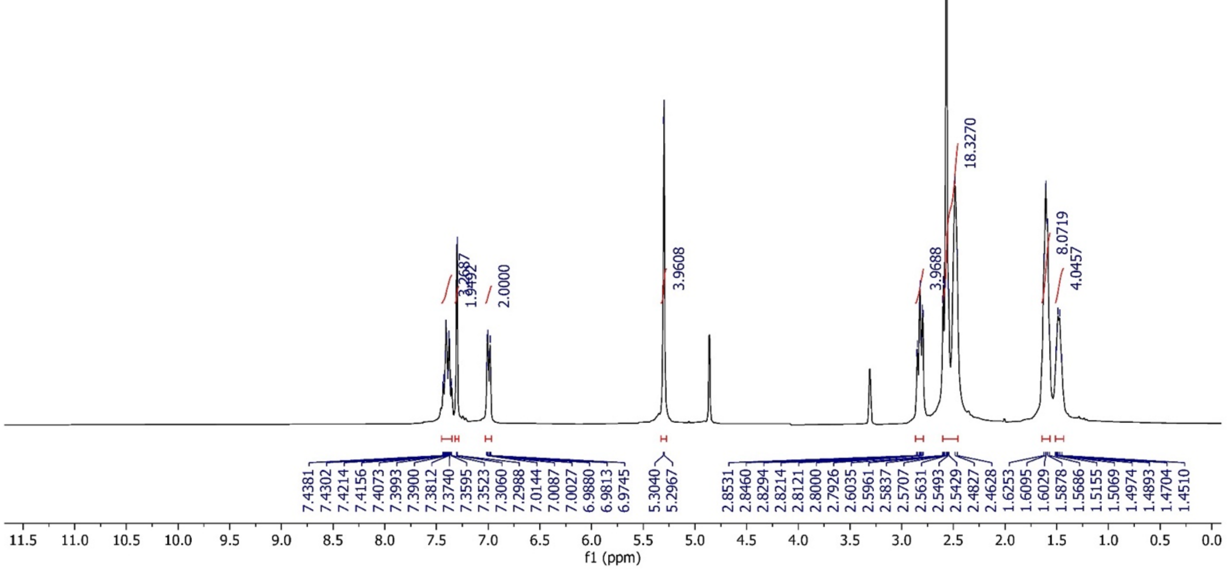

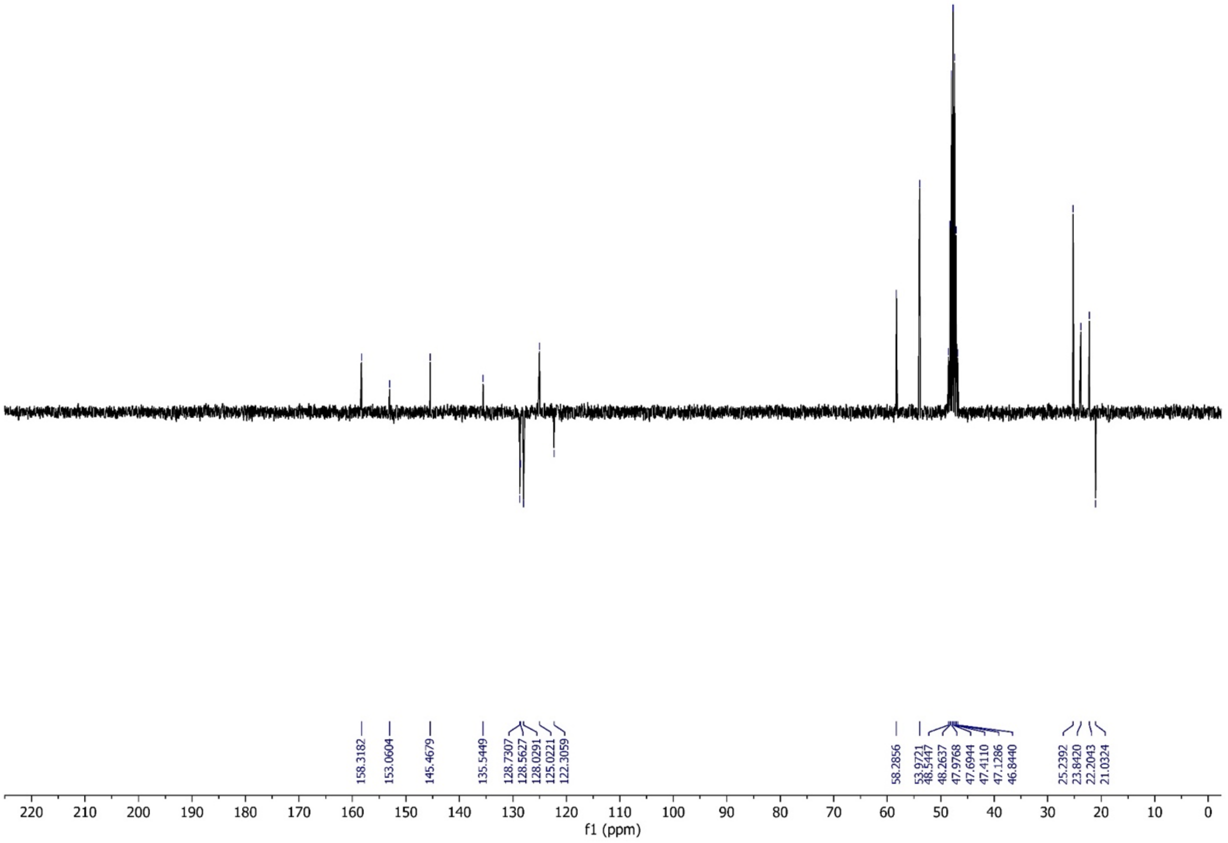

Figure S28. 1D ^{1}H (*top*) and ^{13}C APT (*bottom*) NMR spectra of compound **15b**.

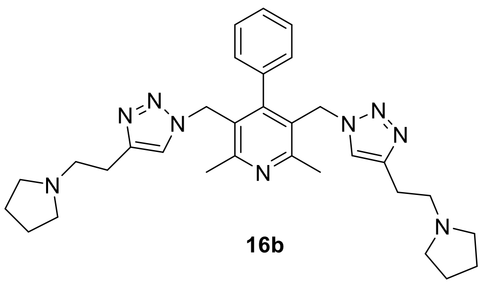

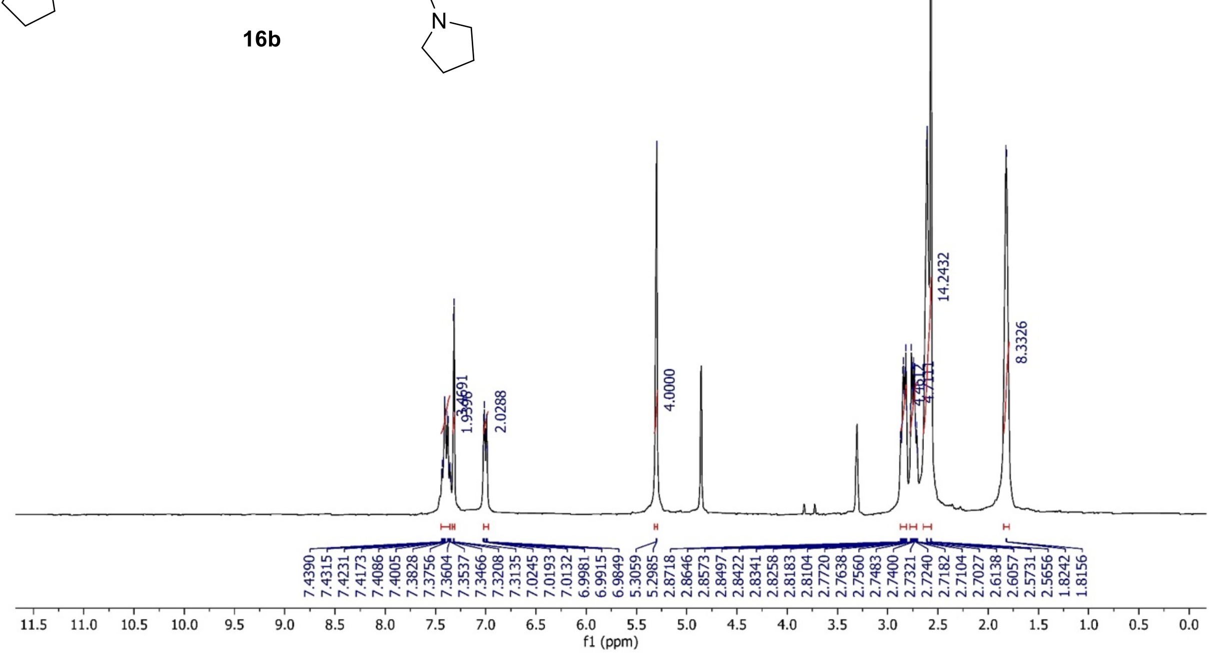

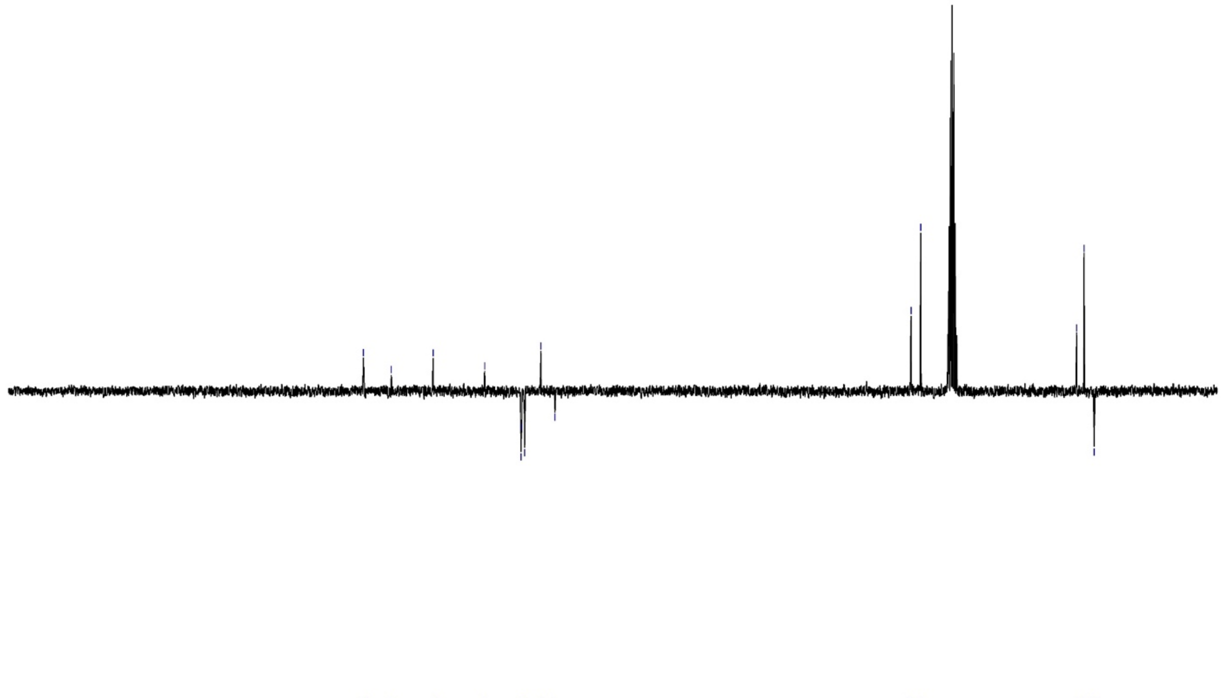

Figure S29. 1D ^{1}H (*top*) and ^{13}C APT (*bottom*) NMR spectra of compound **16b**.

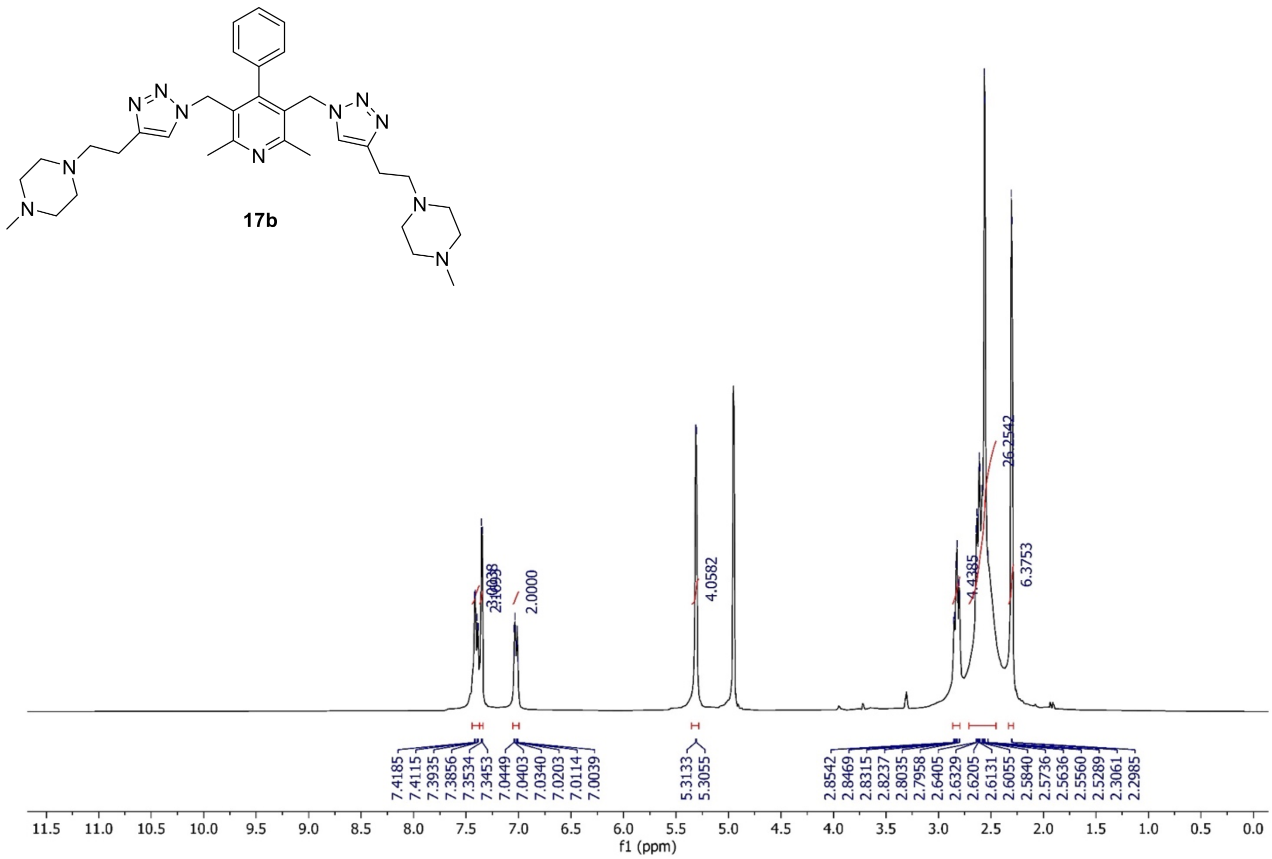

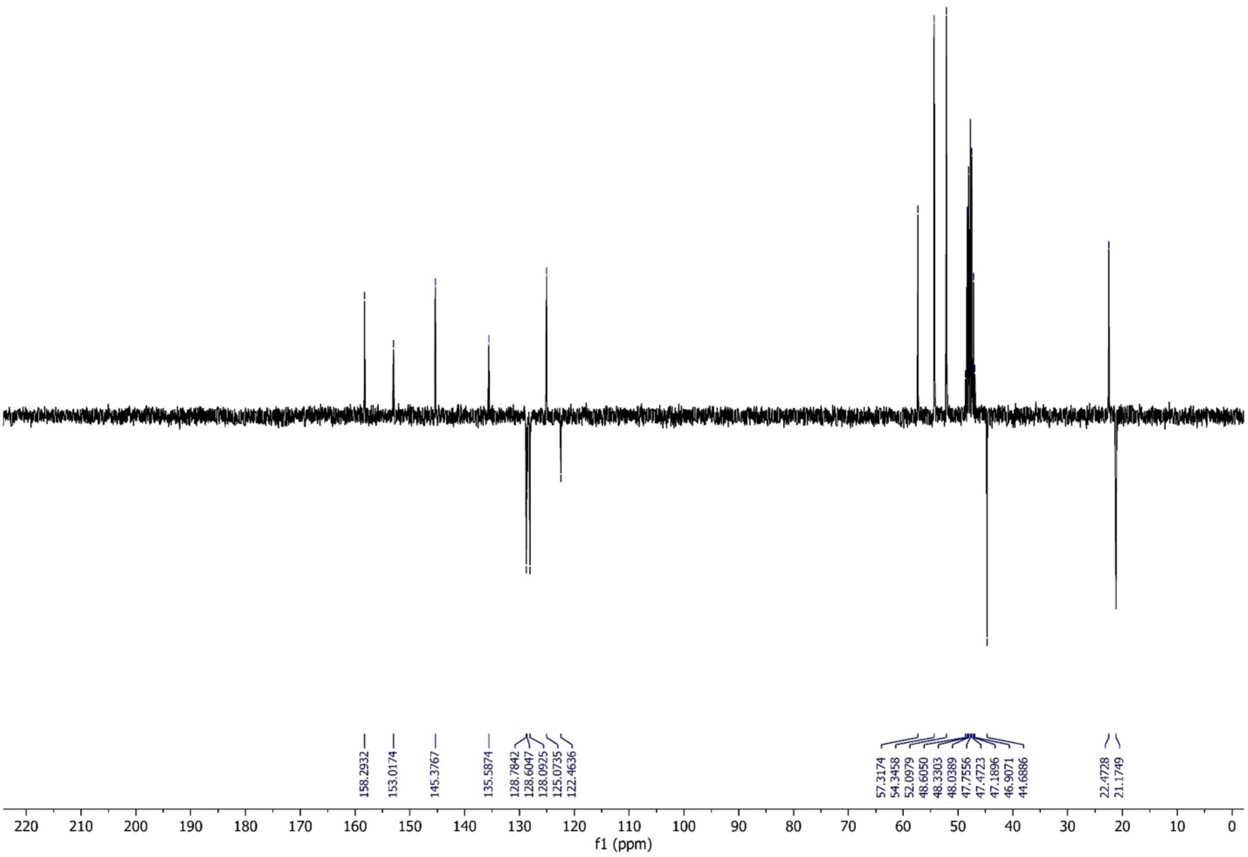

Figure S30. 1D ^{1}H (*top*) and ^{13}C APT (*bottom*) NMR spectra of compound **17b**.

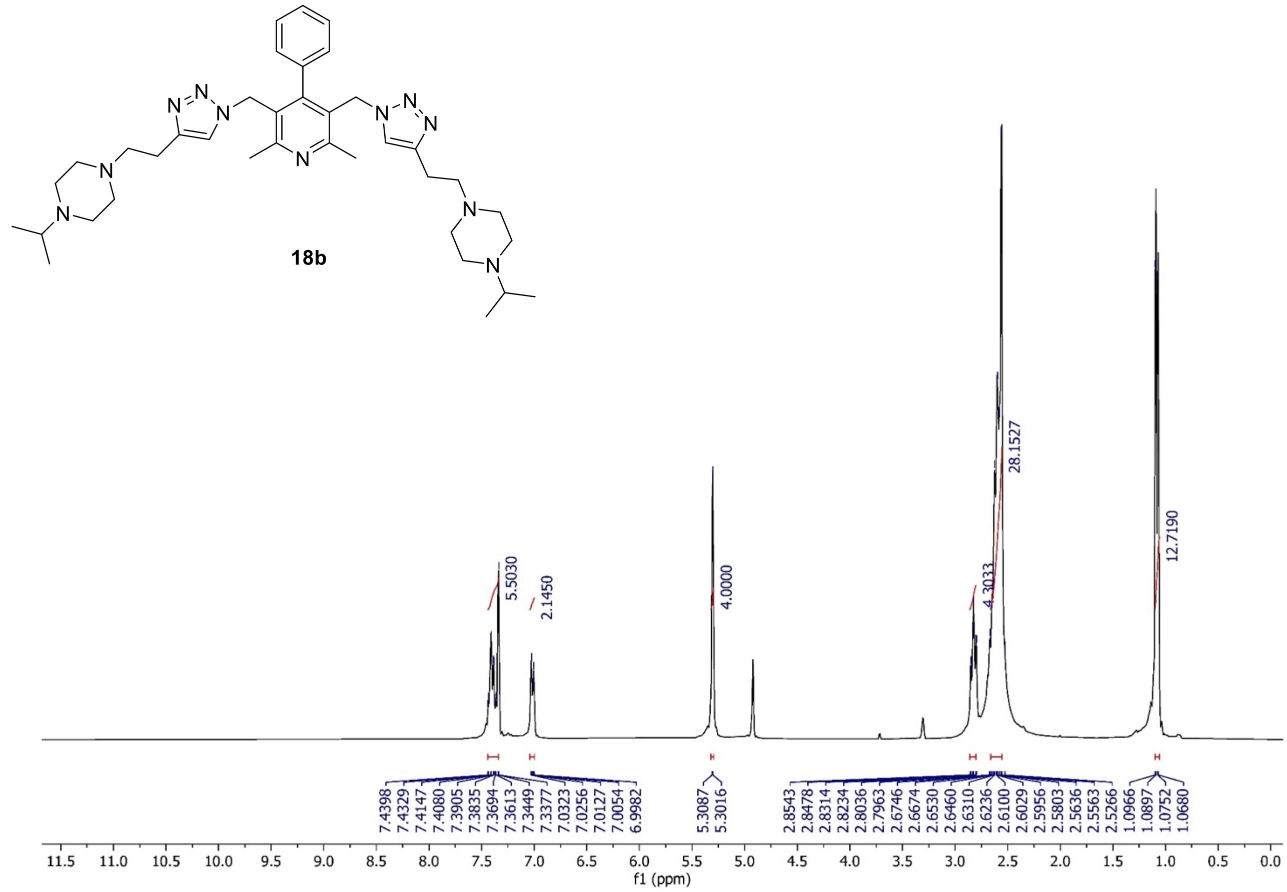

18b

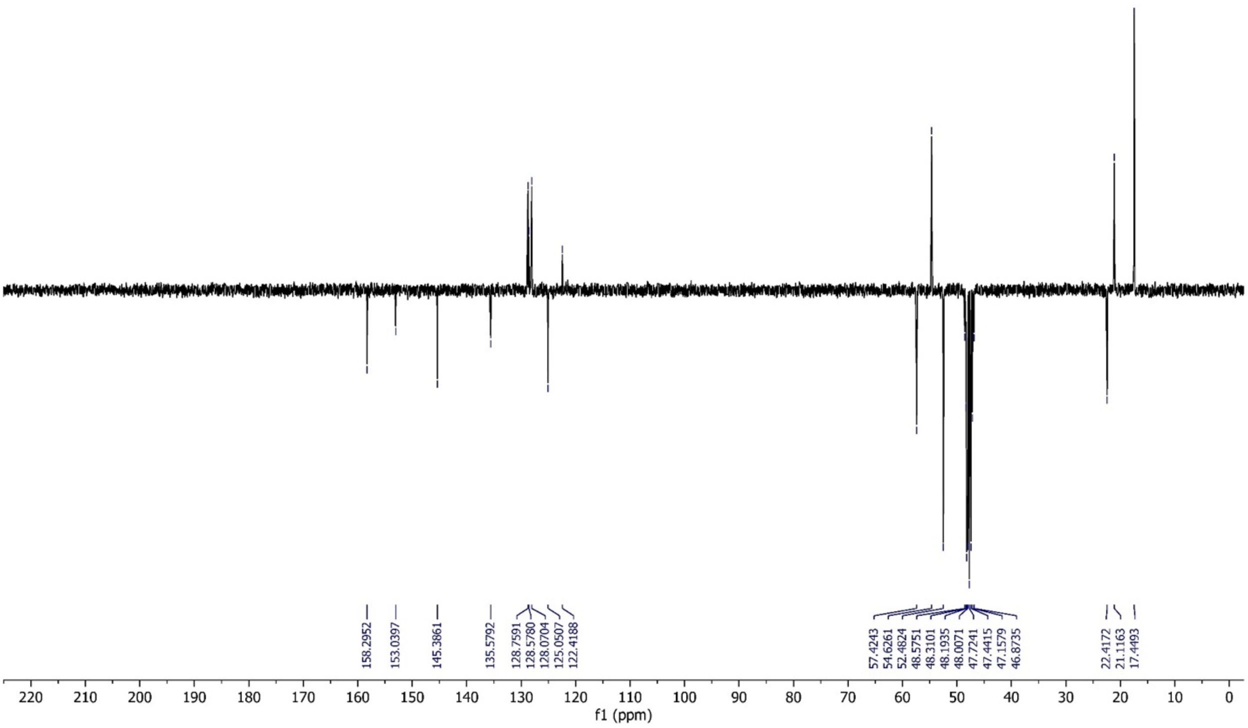

Figure S31. 1D ^{1}H (*top*) and ^{13}C APT (*bottom*) NMR spectra of compound **18b**.

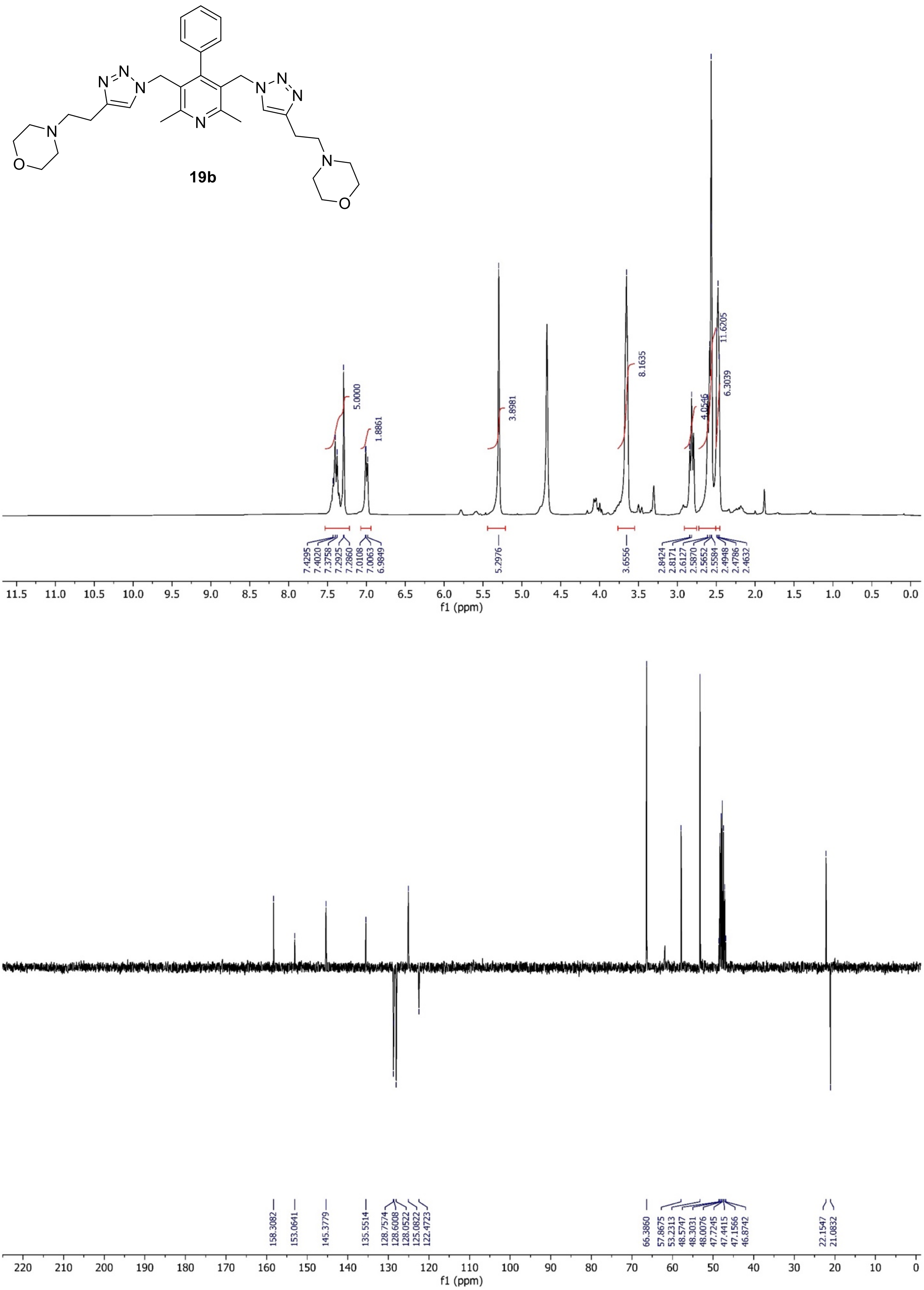

Figure S32. 1D ^{1}H (*top*) and ^{13}C APT (*bottom*) NMR spectra of compound **19b**.

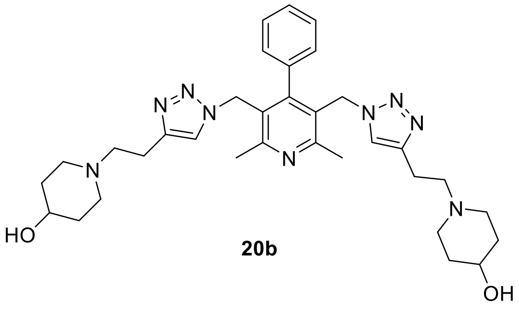

20b

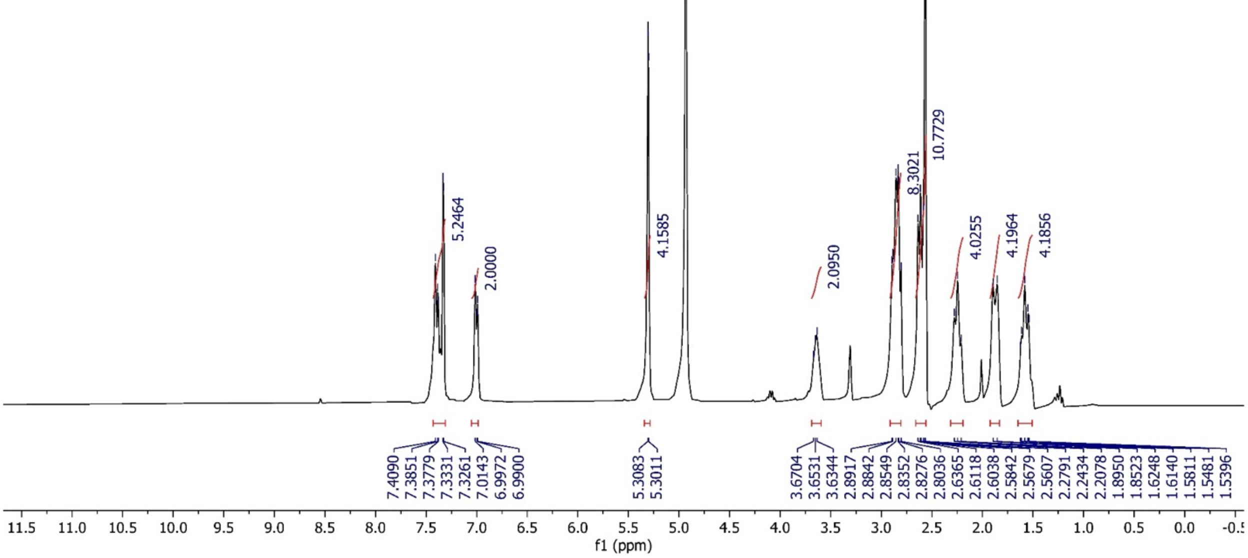

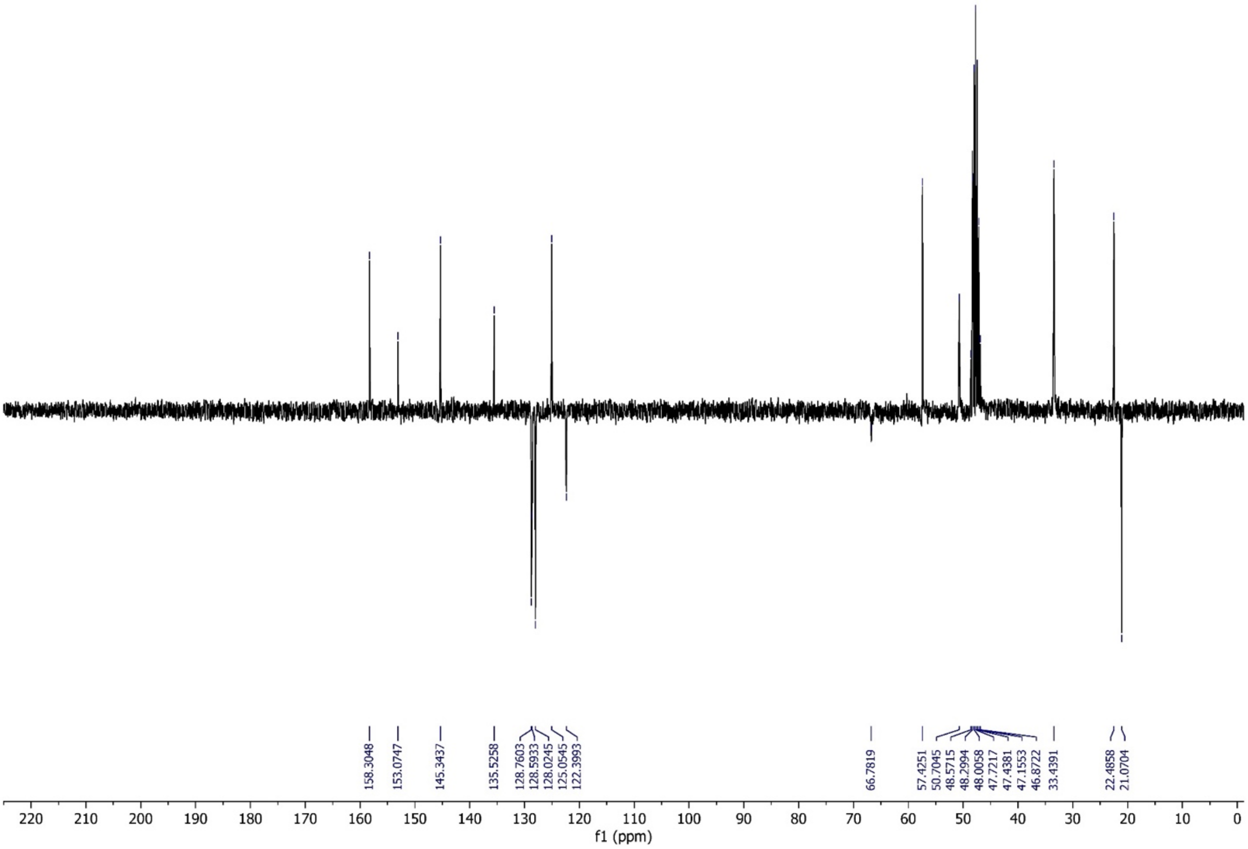

Figure S33. 1D ^{1}H (*top*) and ^{13}C APT (*bottom*) NMR spectra of compound **20b**.

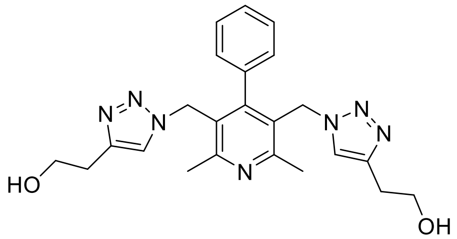

21b

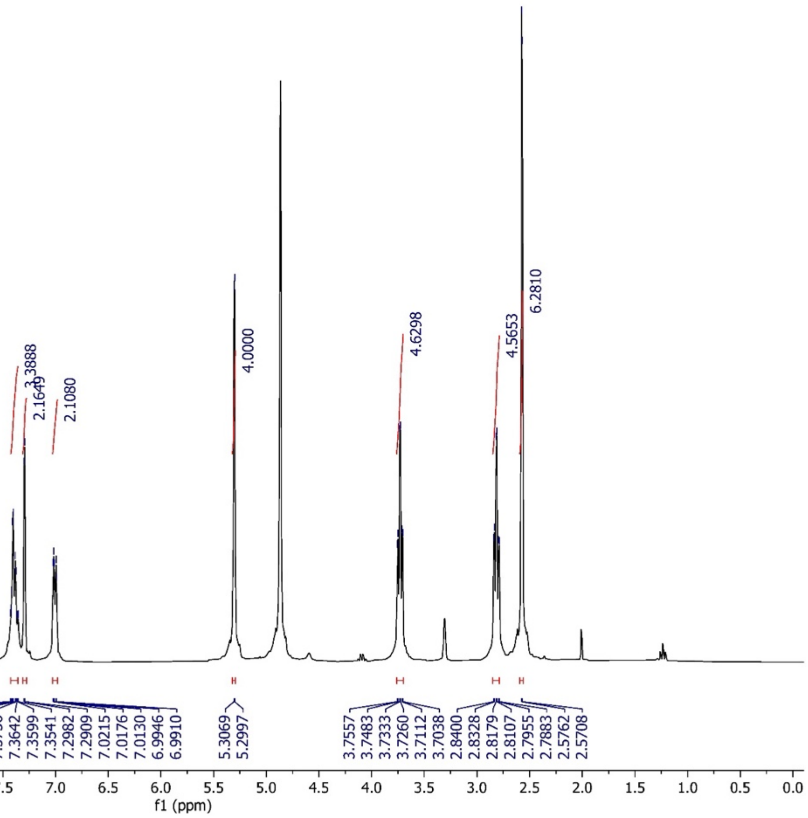

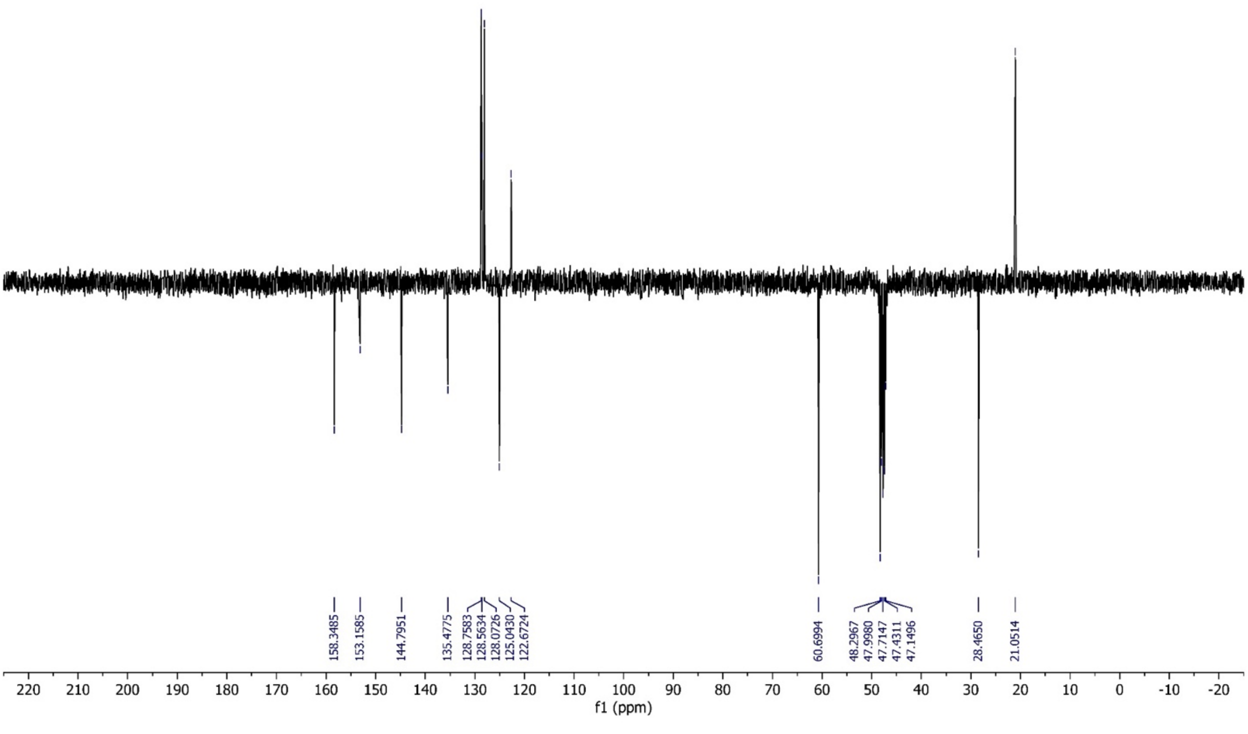

Figure S34. 1D ^{1}H (*top*) and ^{13}C APT (*bottom*) NMR spectra of compound **21b**.

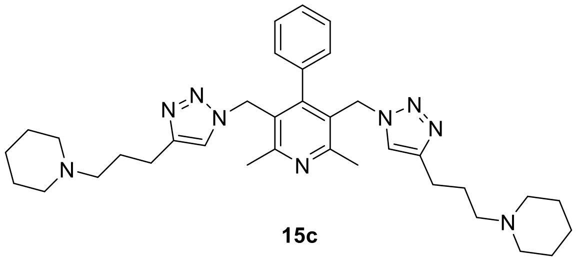

15c

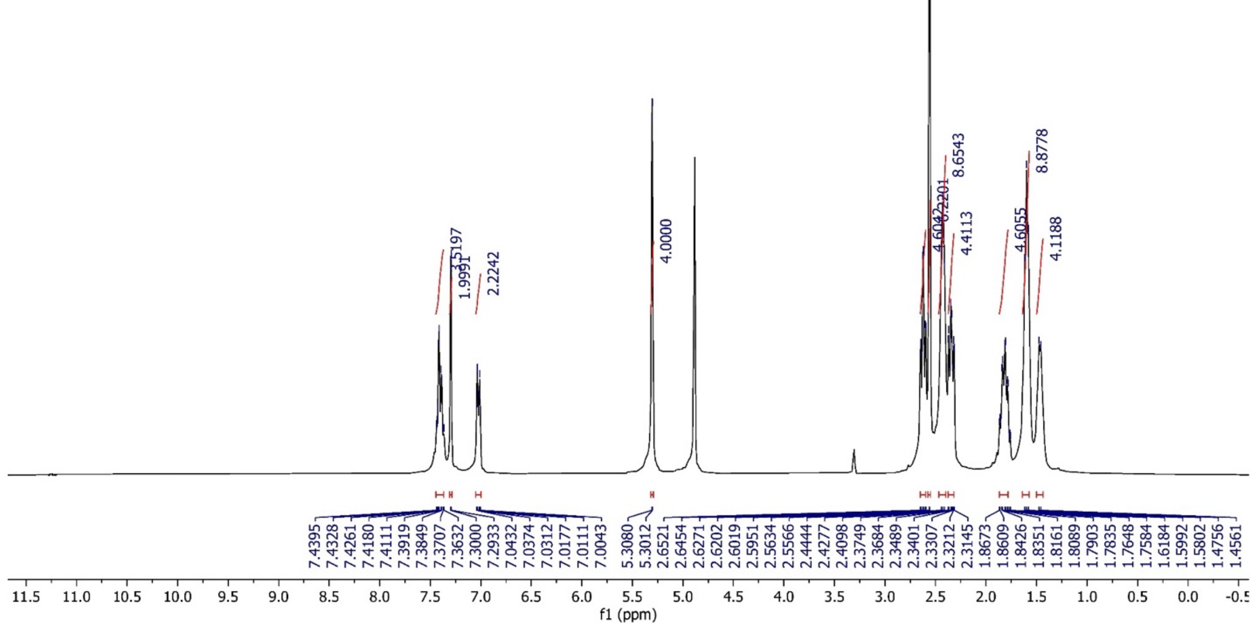

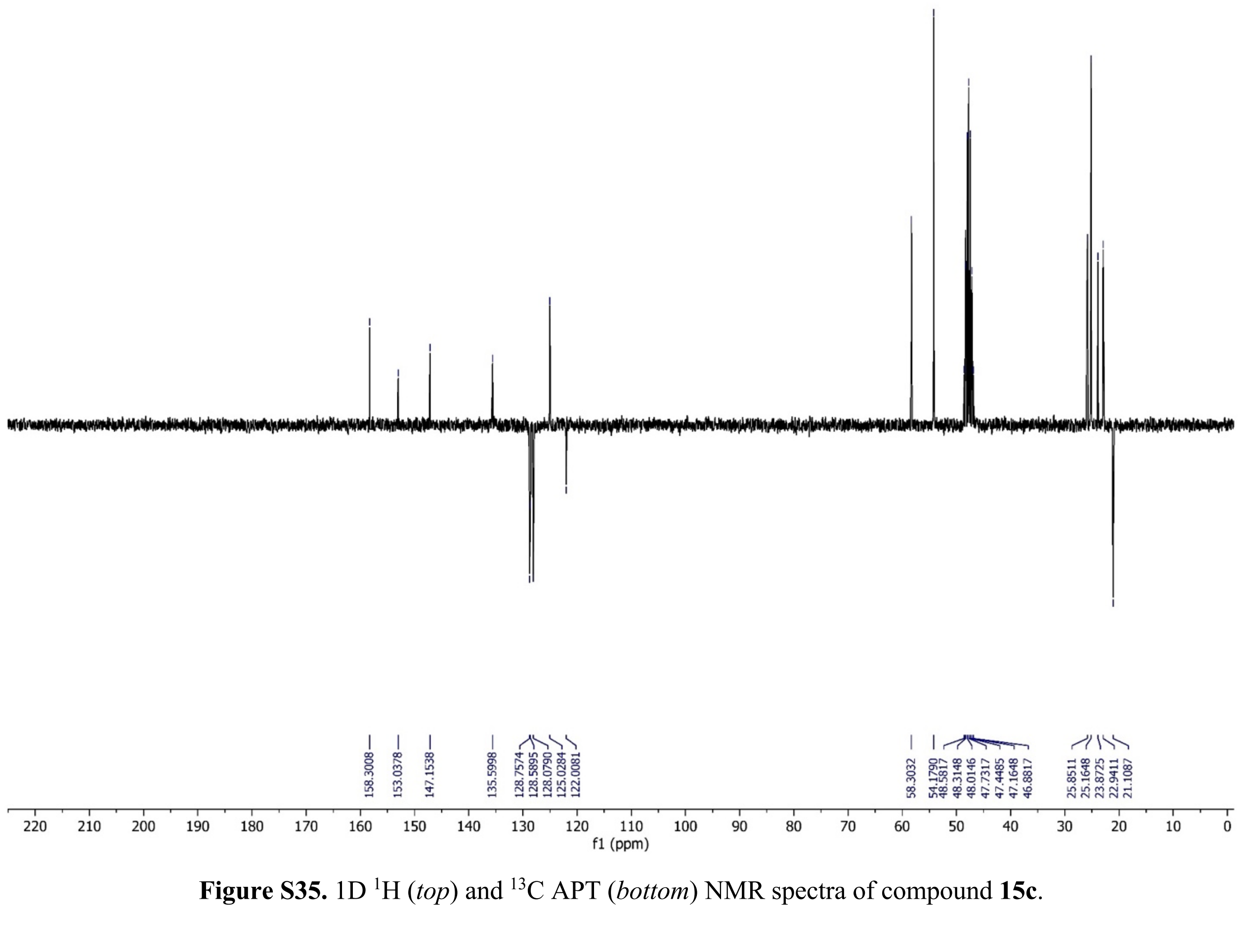

Figure S35. 1D ^{1}H (*top*) and ^{13}C APT (*bottom*) NMR spectra of compound **15c**.

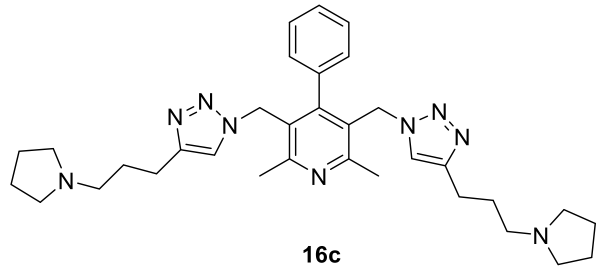

16c

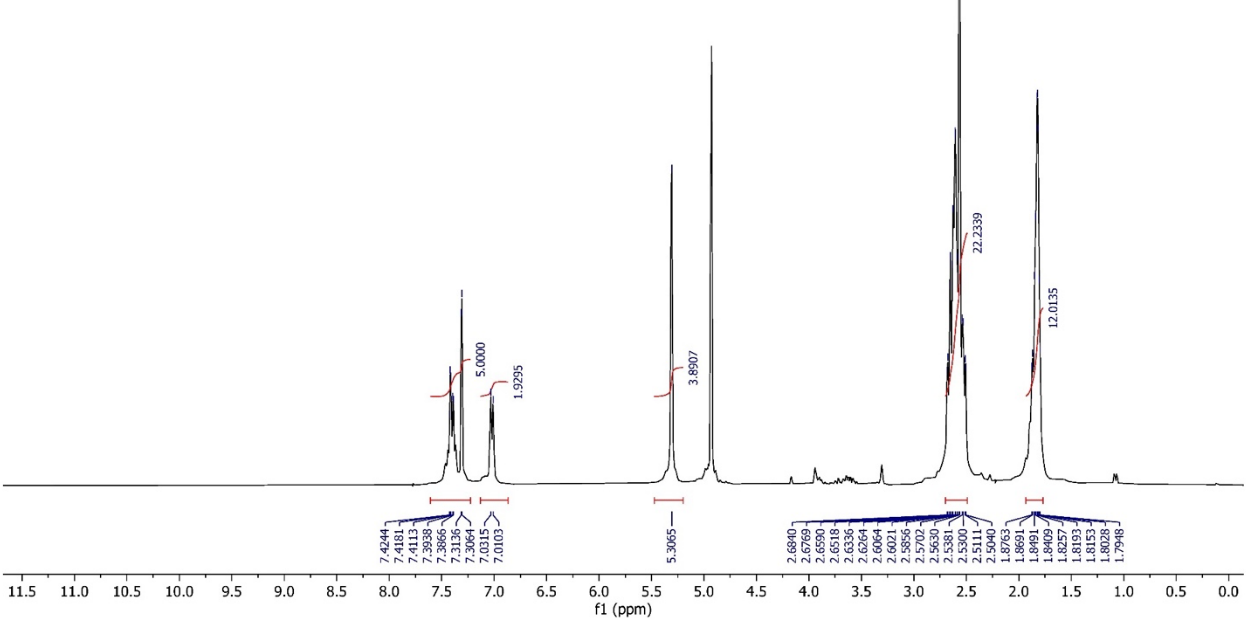

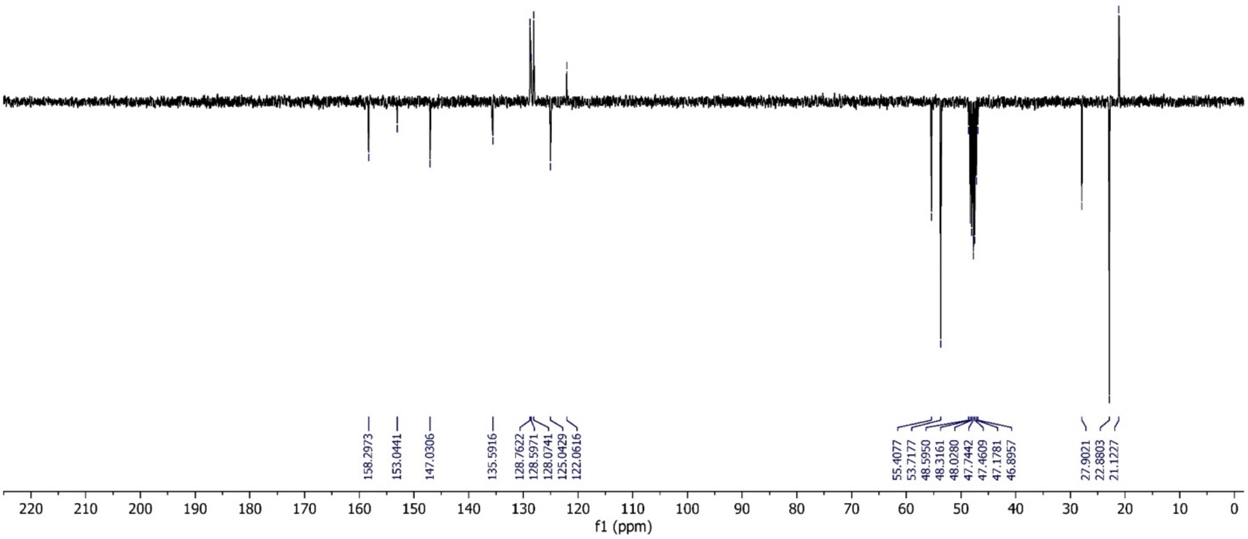

Figure S36. 1D ^{1}H (*top*) and ^{13}C APT (*bottom*) NMR spectra of compound **16c**.

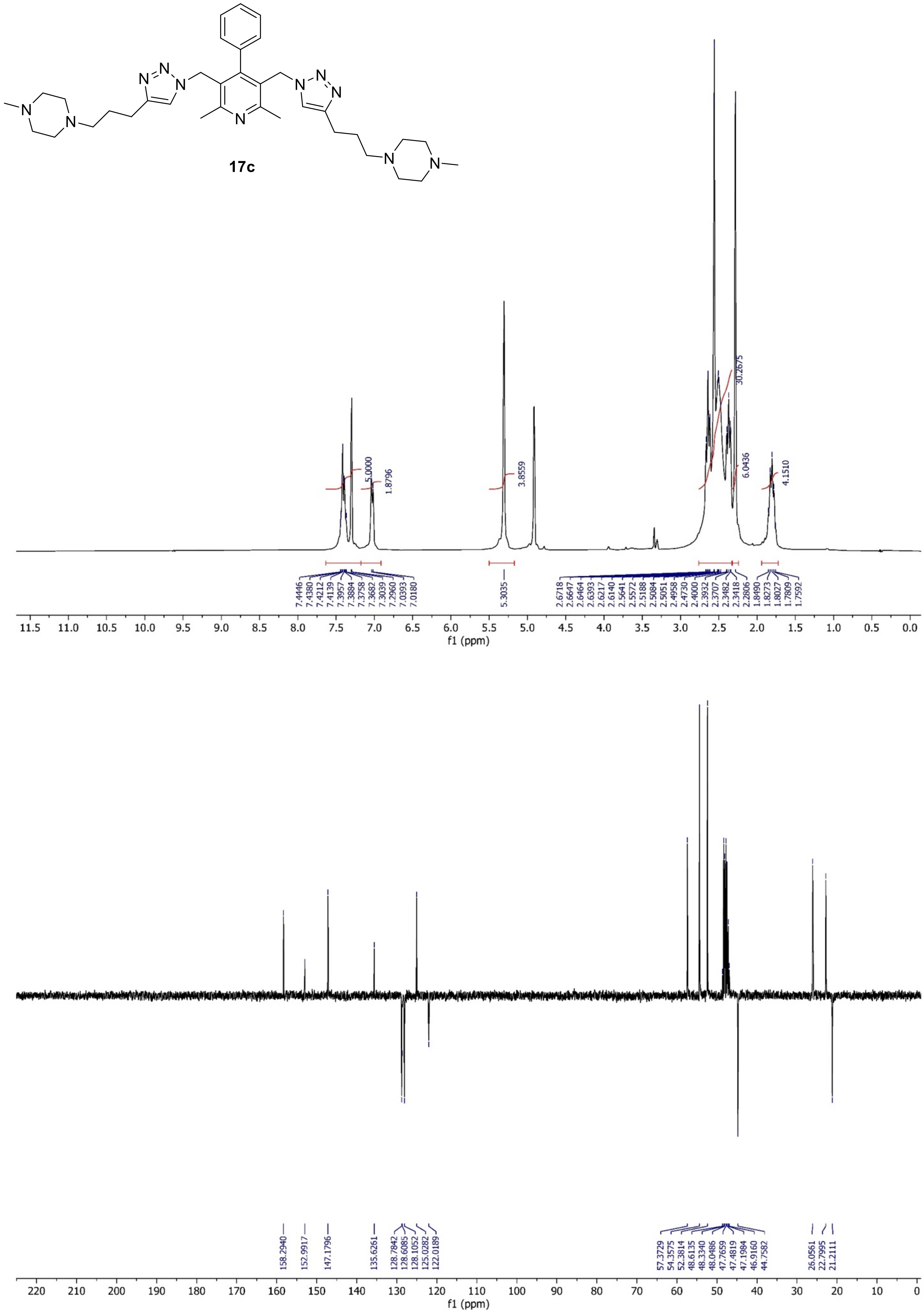

17c

Figure S37. 1D ^{1}H (*top*) and ^{13}C APT (*bottom*) NMR spectra of compound **17c**.

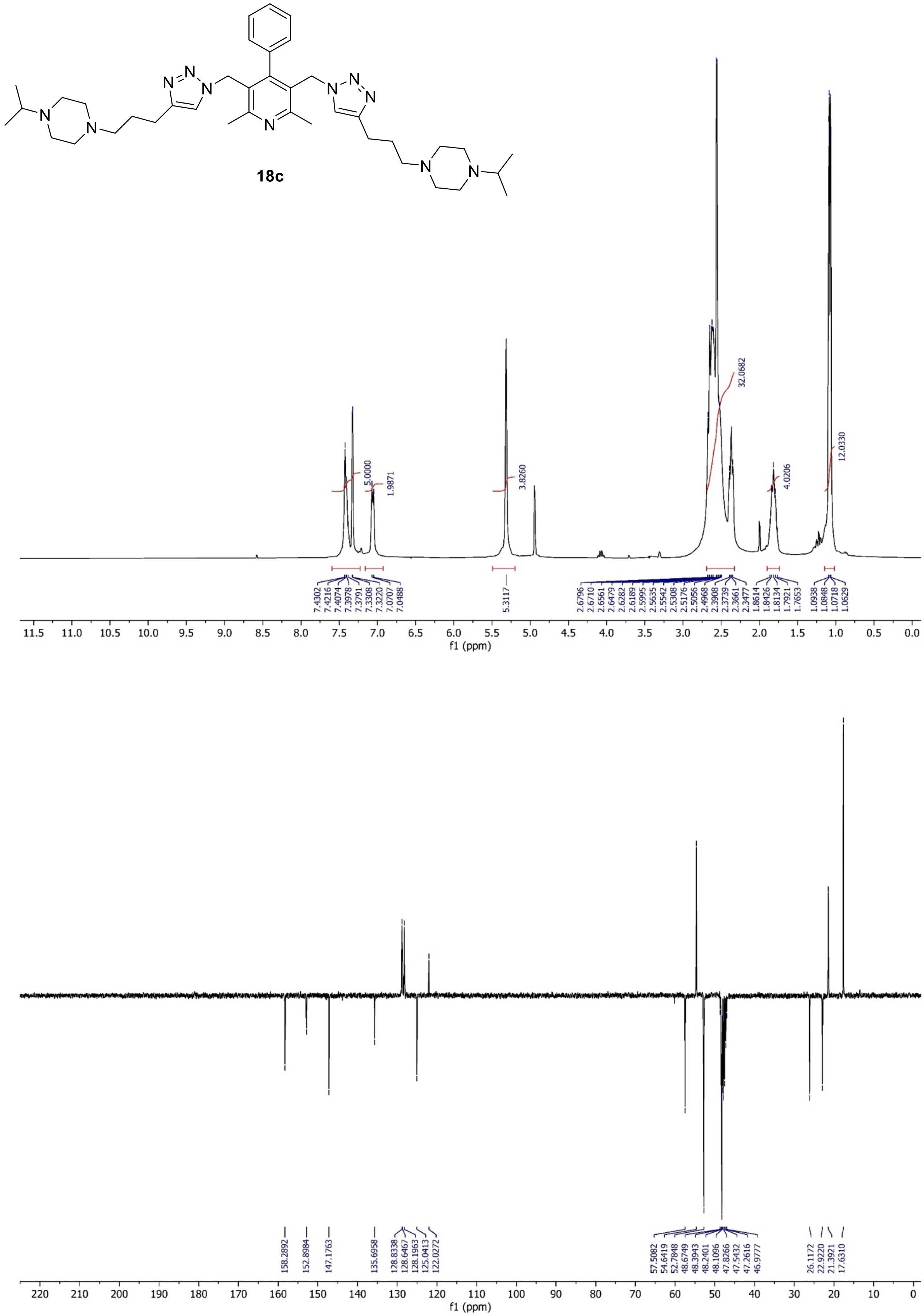

Figure S38. 1D ^{1}H (*top*) and ^{13}C APT (*bottom*) NMR spectra of compound **18c**.

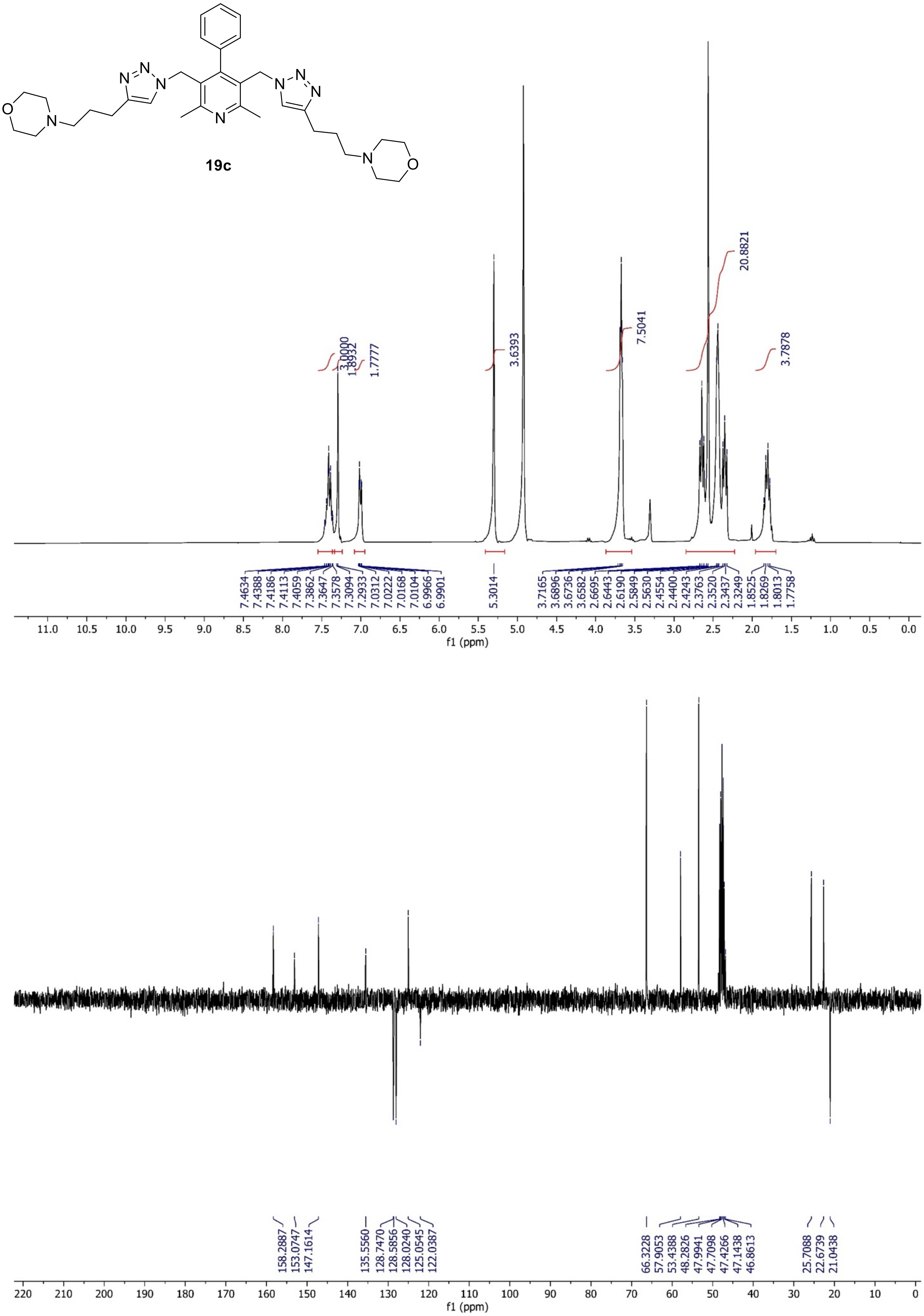

Figure S39. 1D ^{1}H (*top*) and ^{13}C APT (*bottom*) NMR spectra of compound **19c**.

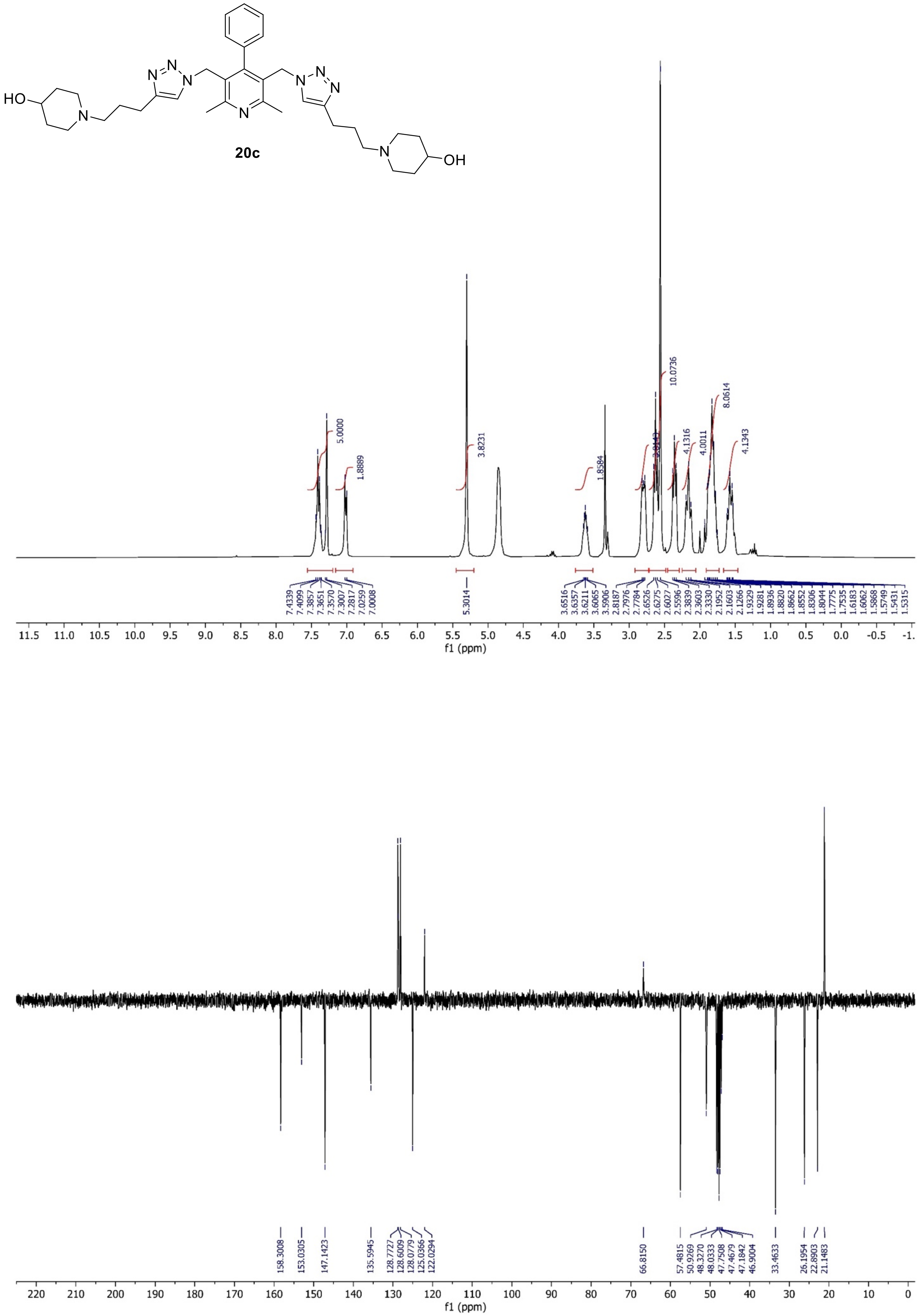

Figure S40. 1D ^{1}H (*top*) and ^{13}C APT (*bottom*) NMR spectra of compound **20c**.

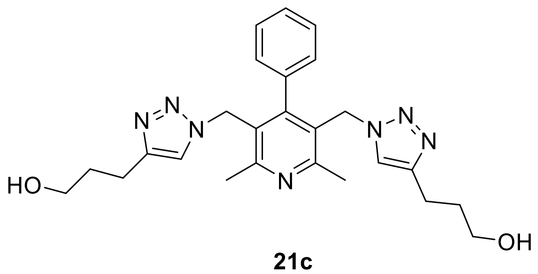

21c

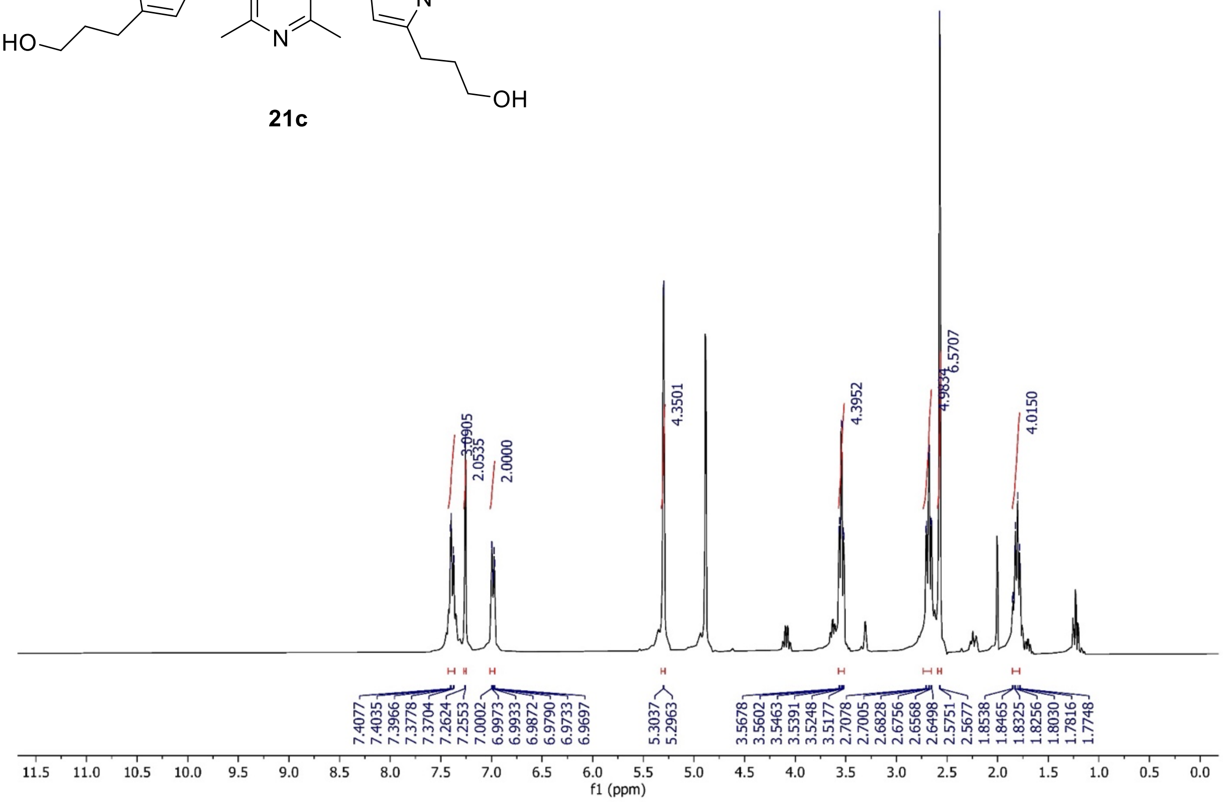

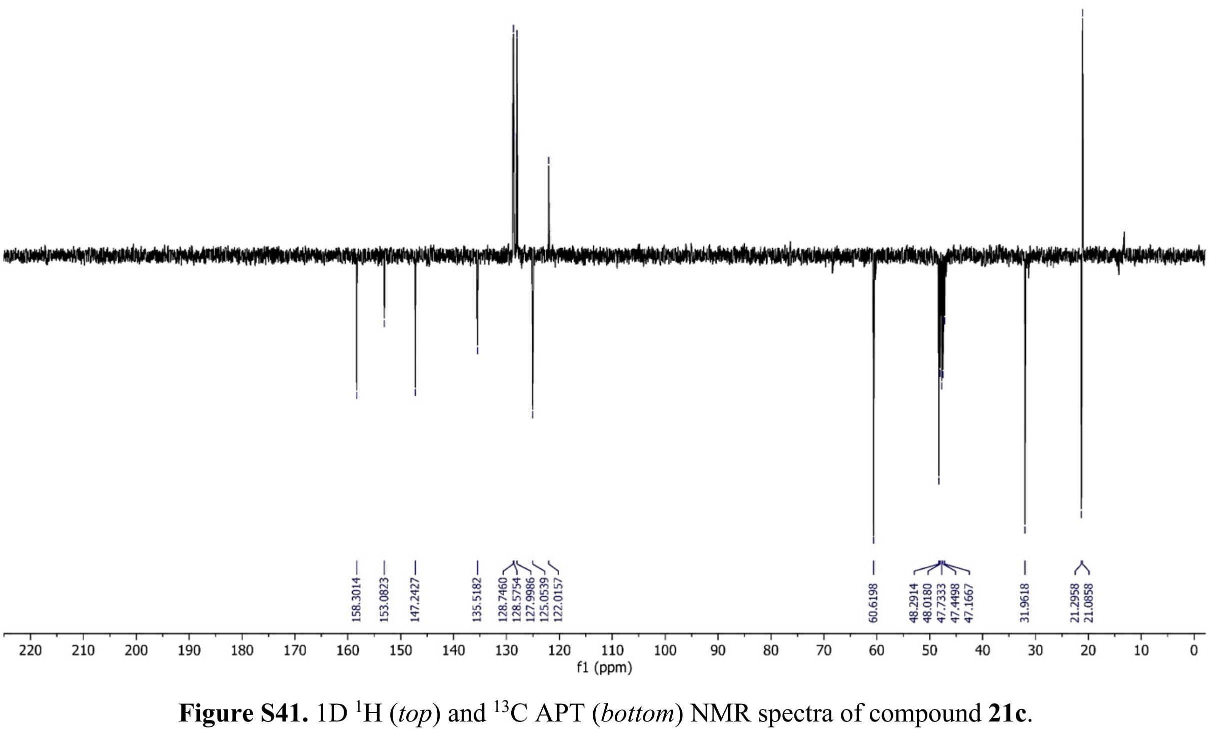

Figure S41. 1D ^{1}H (*top*) and ^{13}C APT (*bottom*) NMR spectra of compound **21c**.

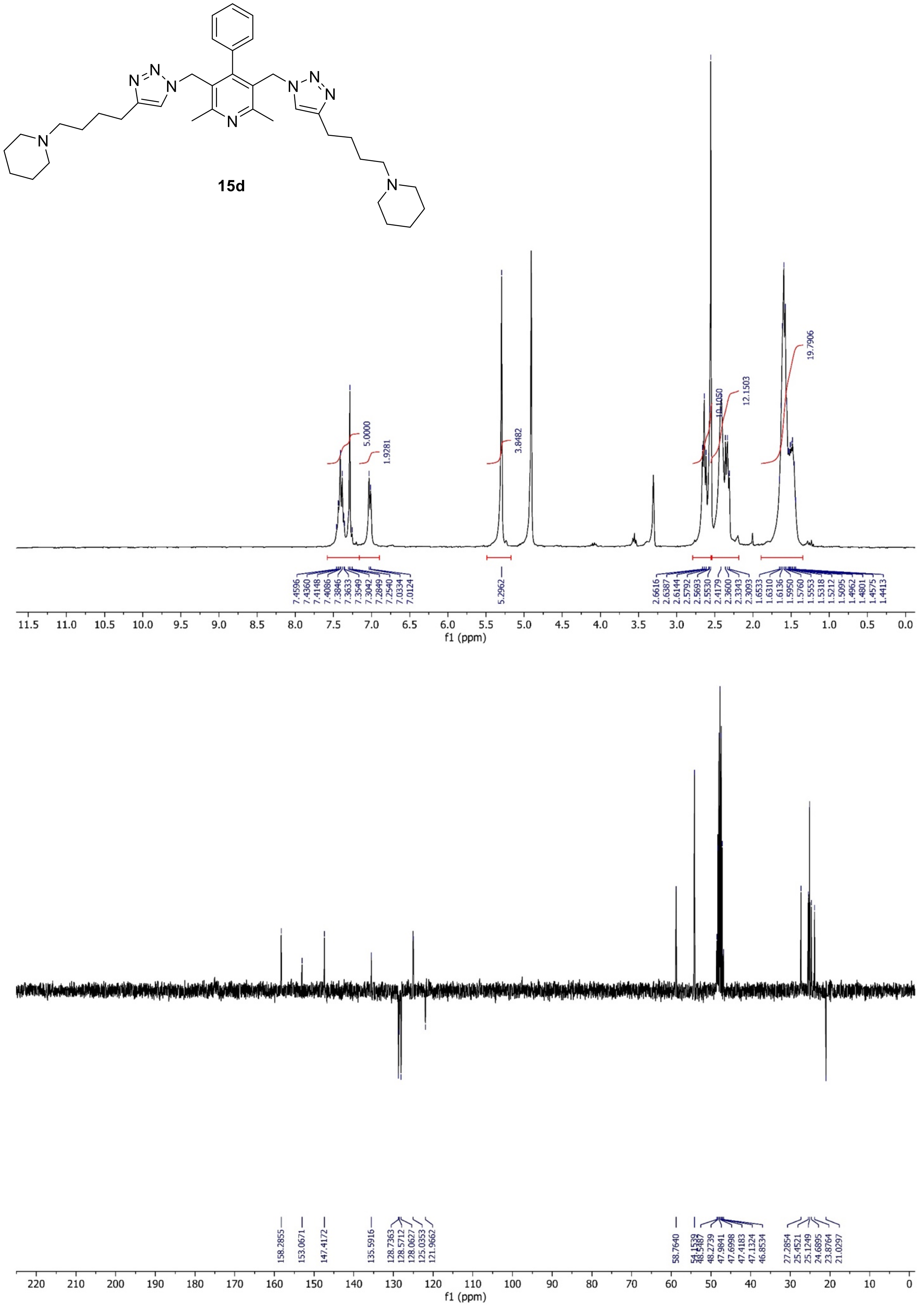

Figure S42. 1D ^{1}H (*top*) and ^{13}C APT (*bottom*) NMR spectra of compound **15d**.

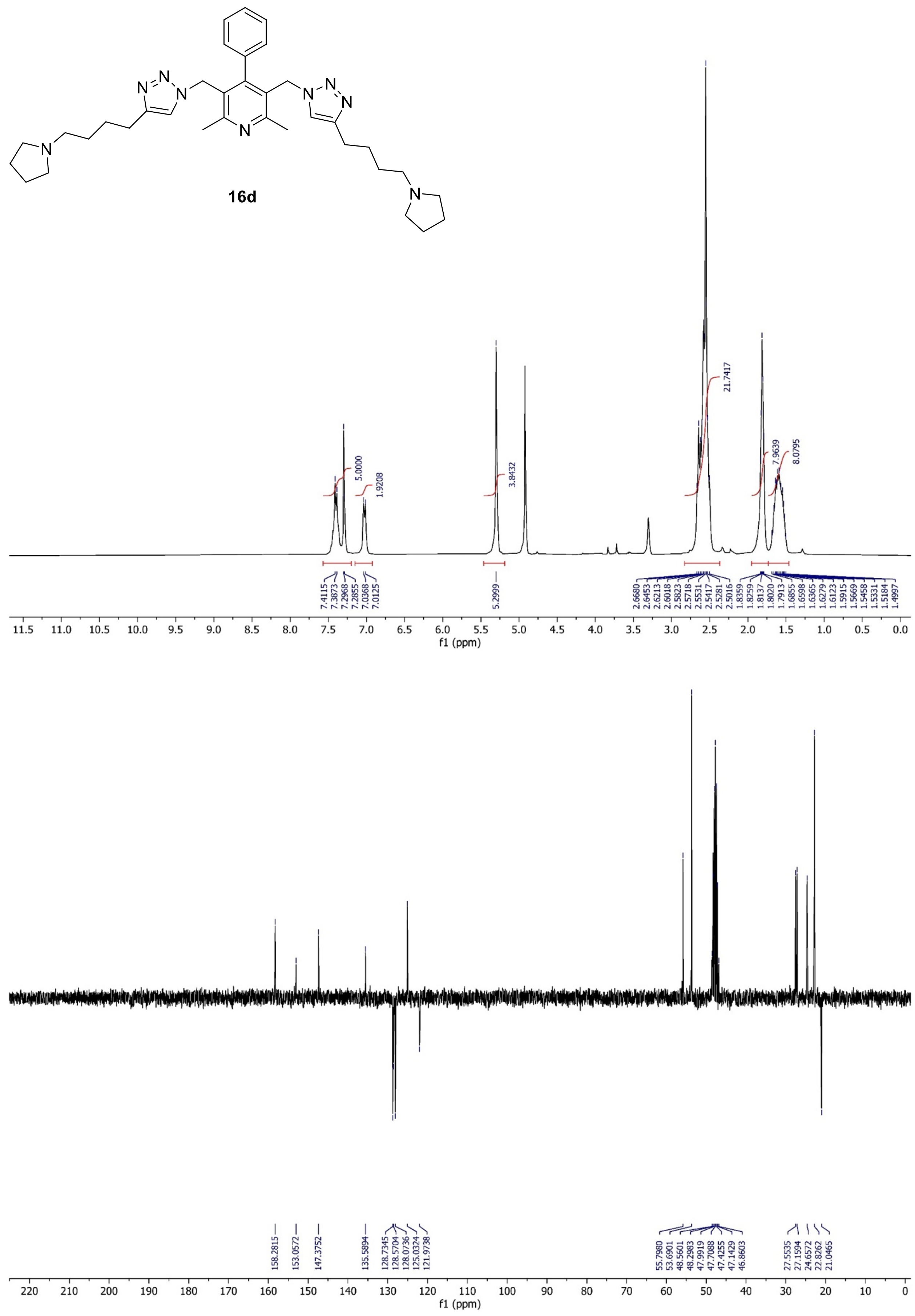

Figure S43. 1D ^{1}H (*top*) and ^{13}C APT (*bottom*) NMR spectra of compound **16d**.

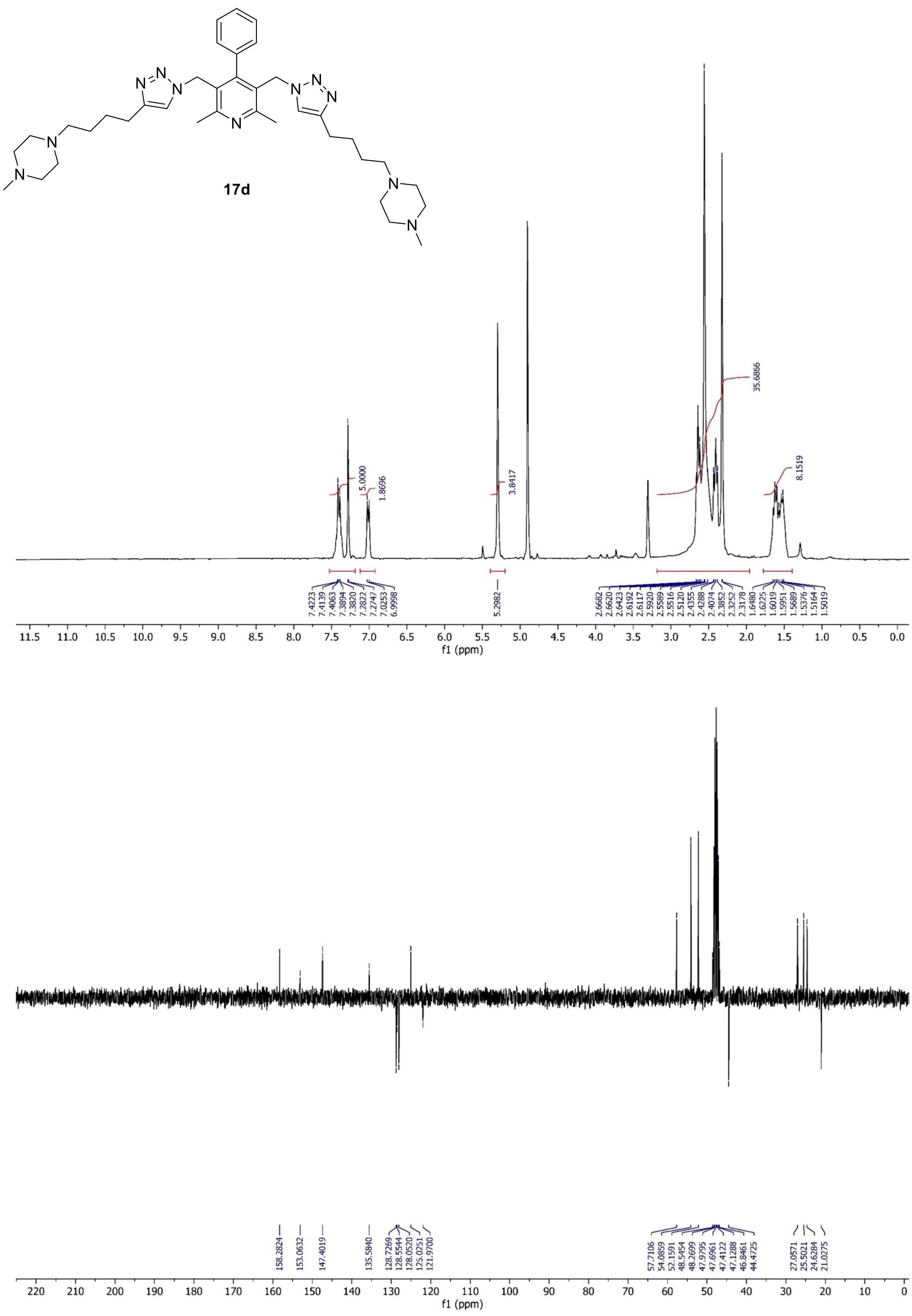

Figure S44. 1D ^{1}H (*top*) and ^{13}C APT (*bottom*) NMR spectra of compound **17d**.

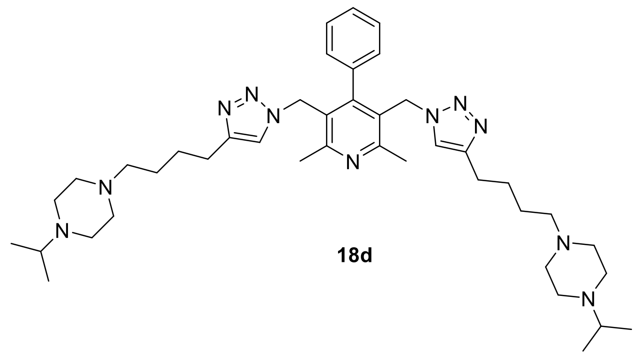

18d

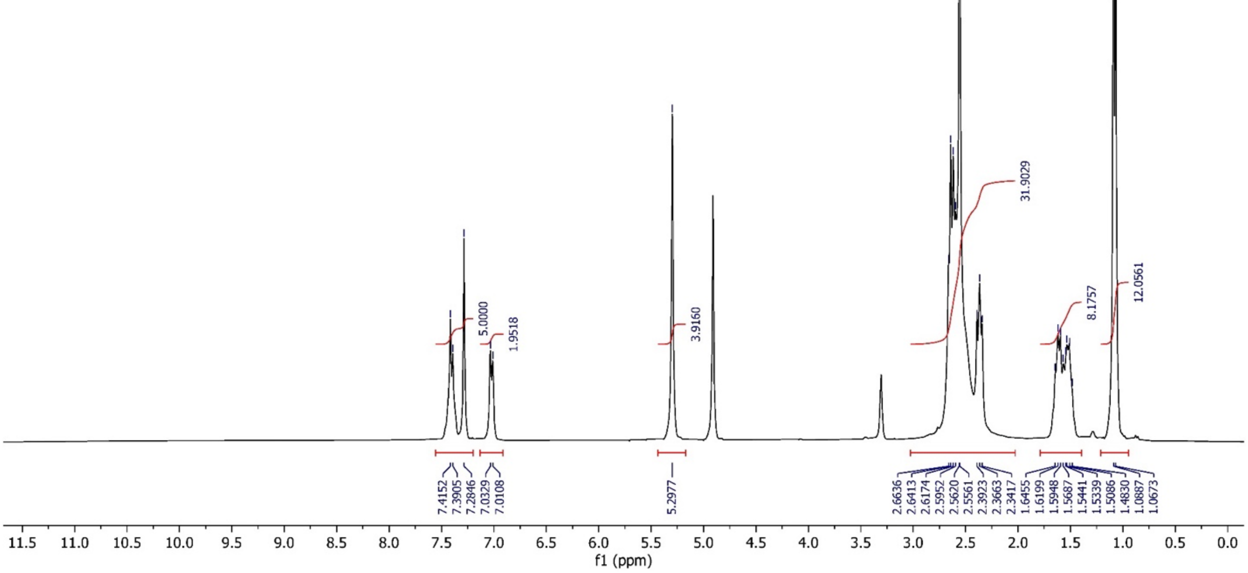

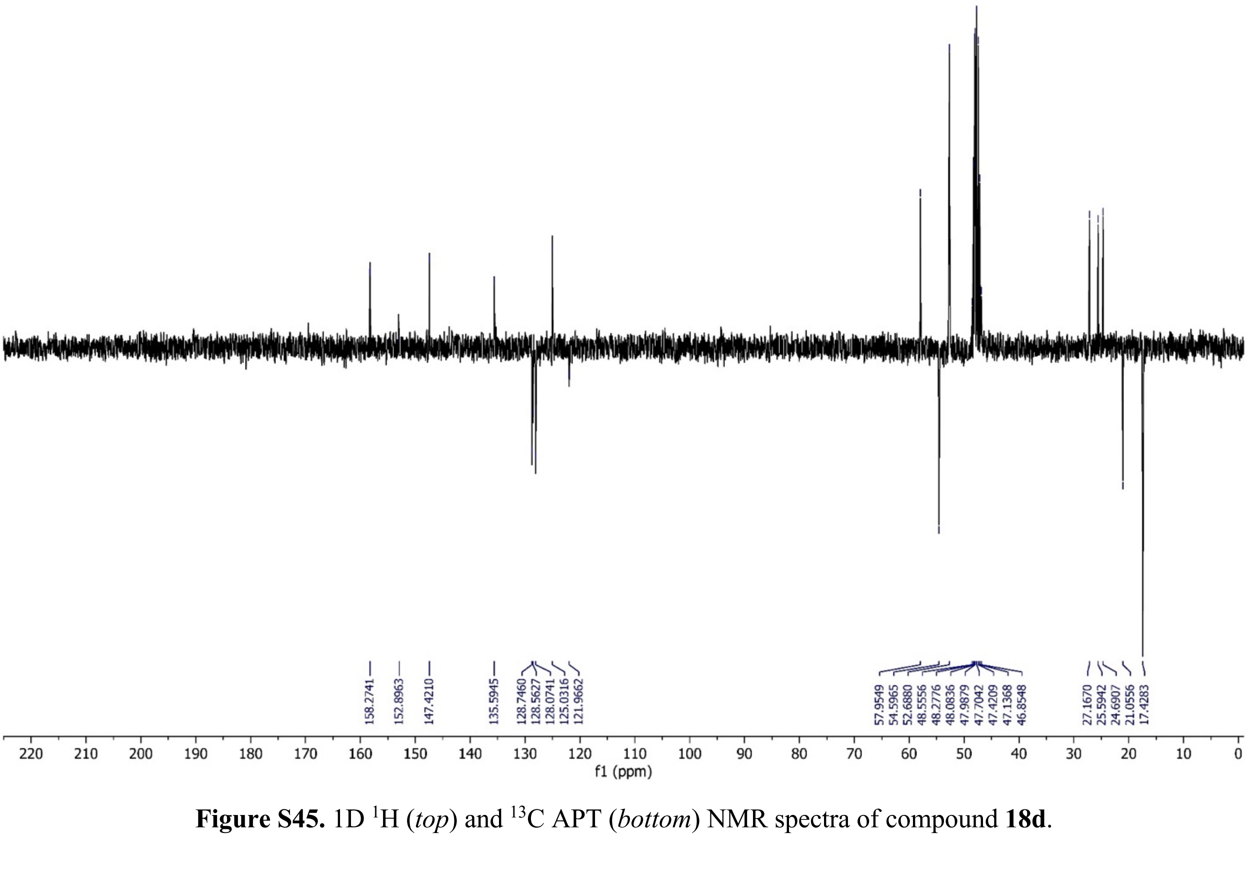

Figure S45. 1D ^{1}H (*top*) and ^{13}C APT (*bottom*) NMR spectra of compound **18d**.

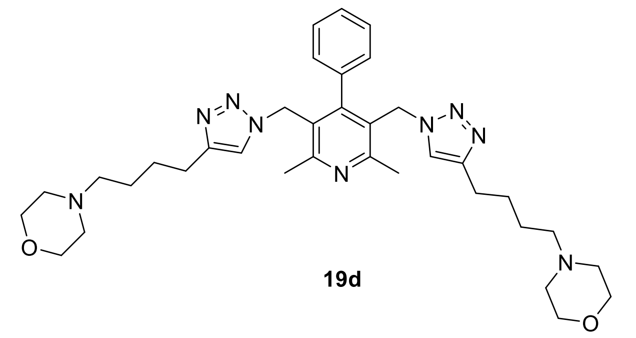

19d

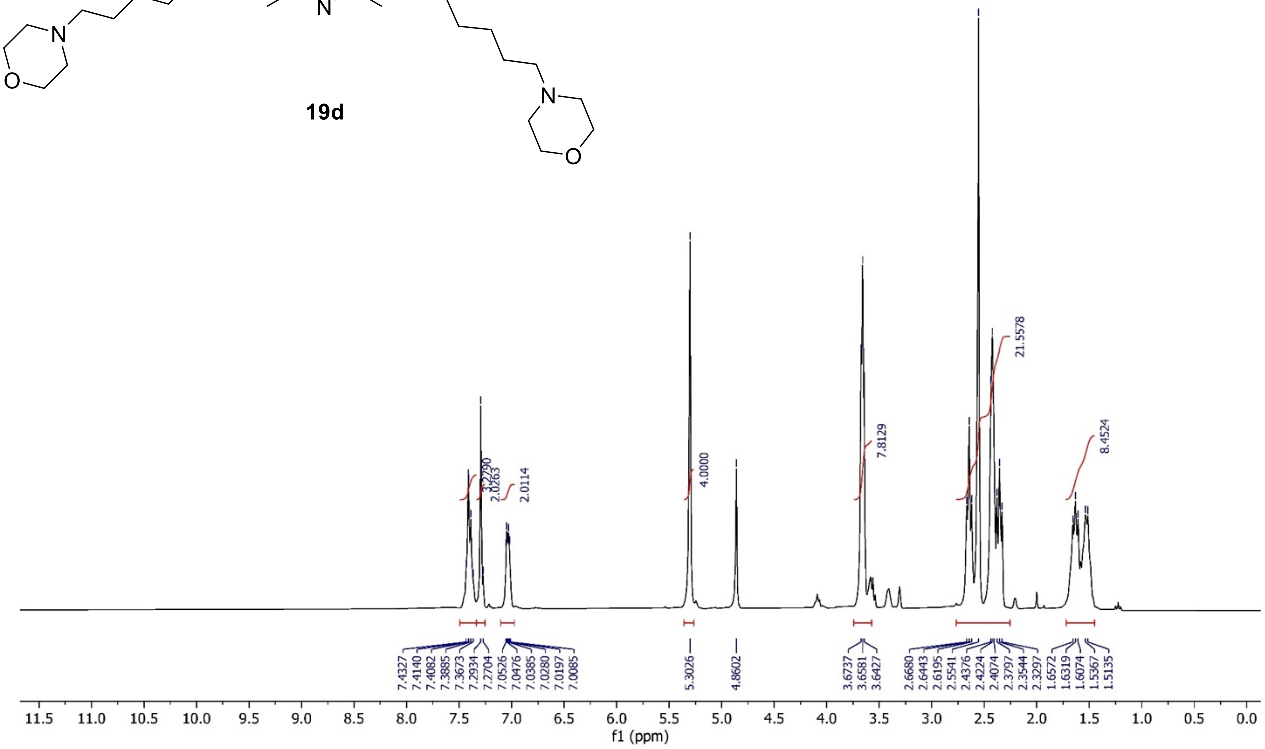

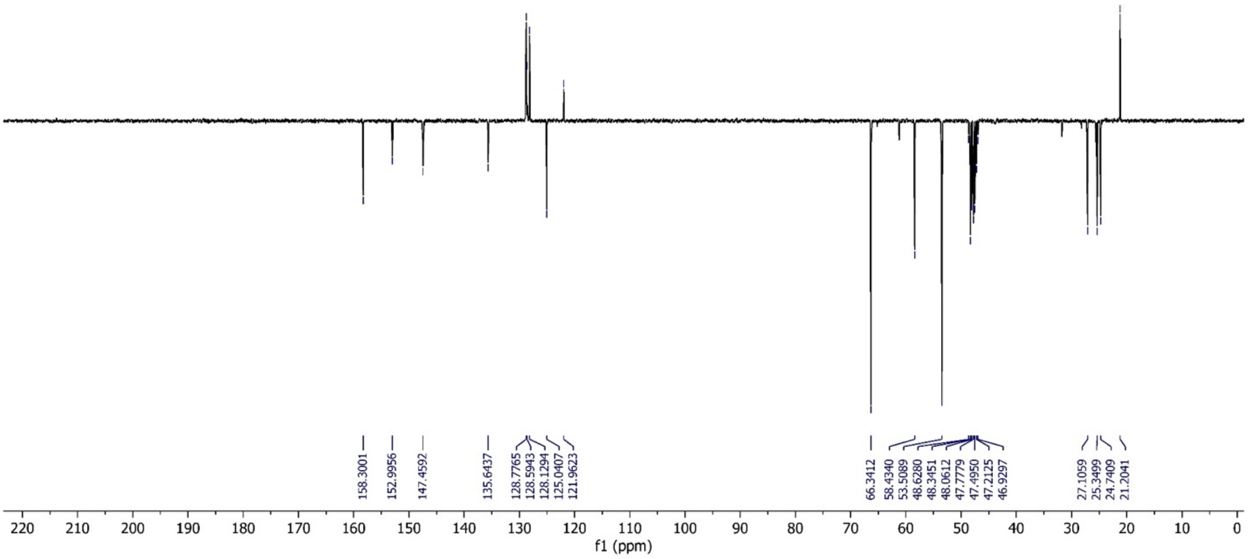

Figure S46. 1D ^{1}H (*top*) and ^{13}C APT (*bottom*) NMR spectra of compound **19d**.

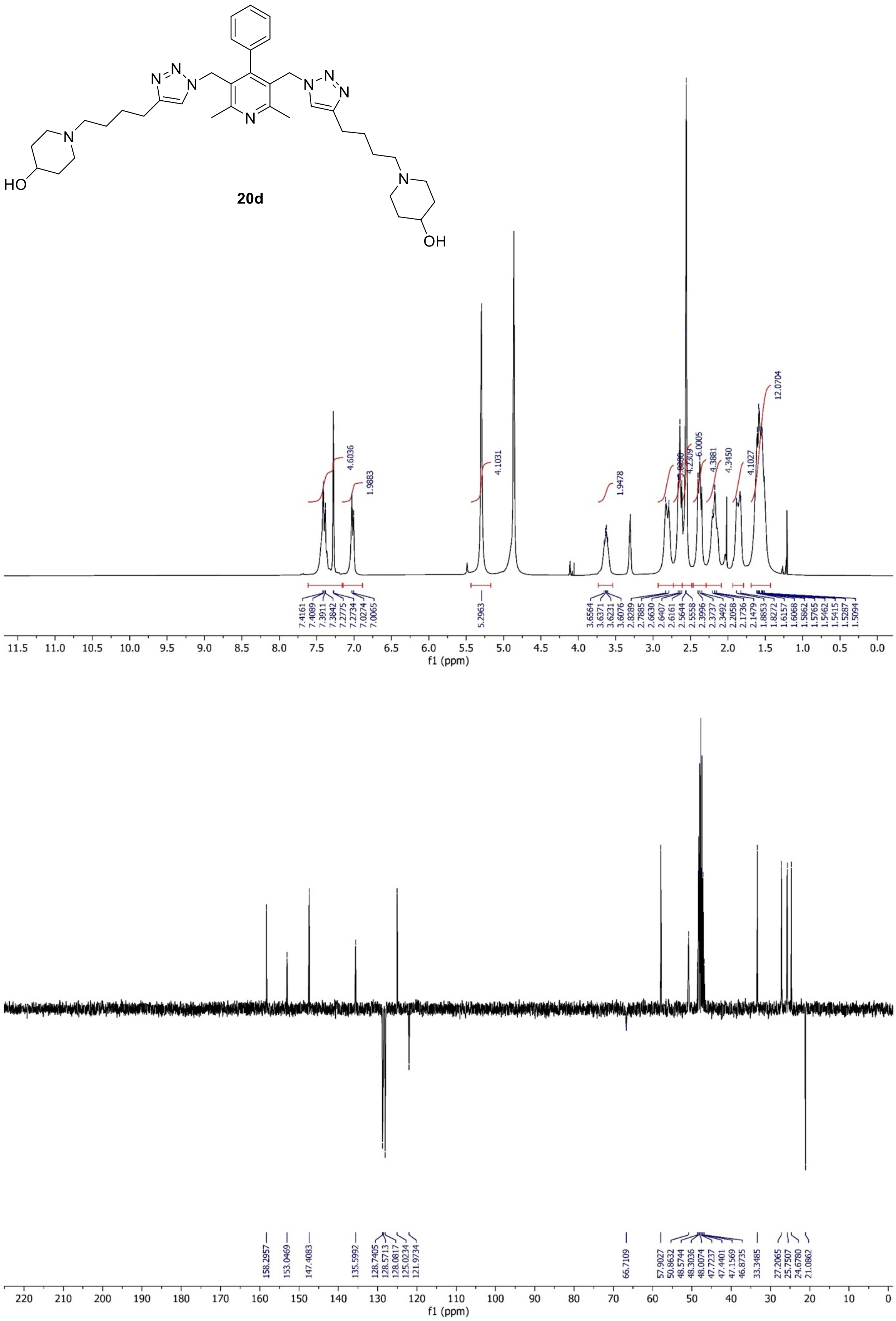

Figure S47. 1D ^{1}H (*top*) and ^{13}C APT (*bottom*) NMR spectra of compound **20d**.

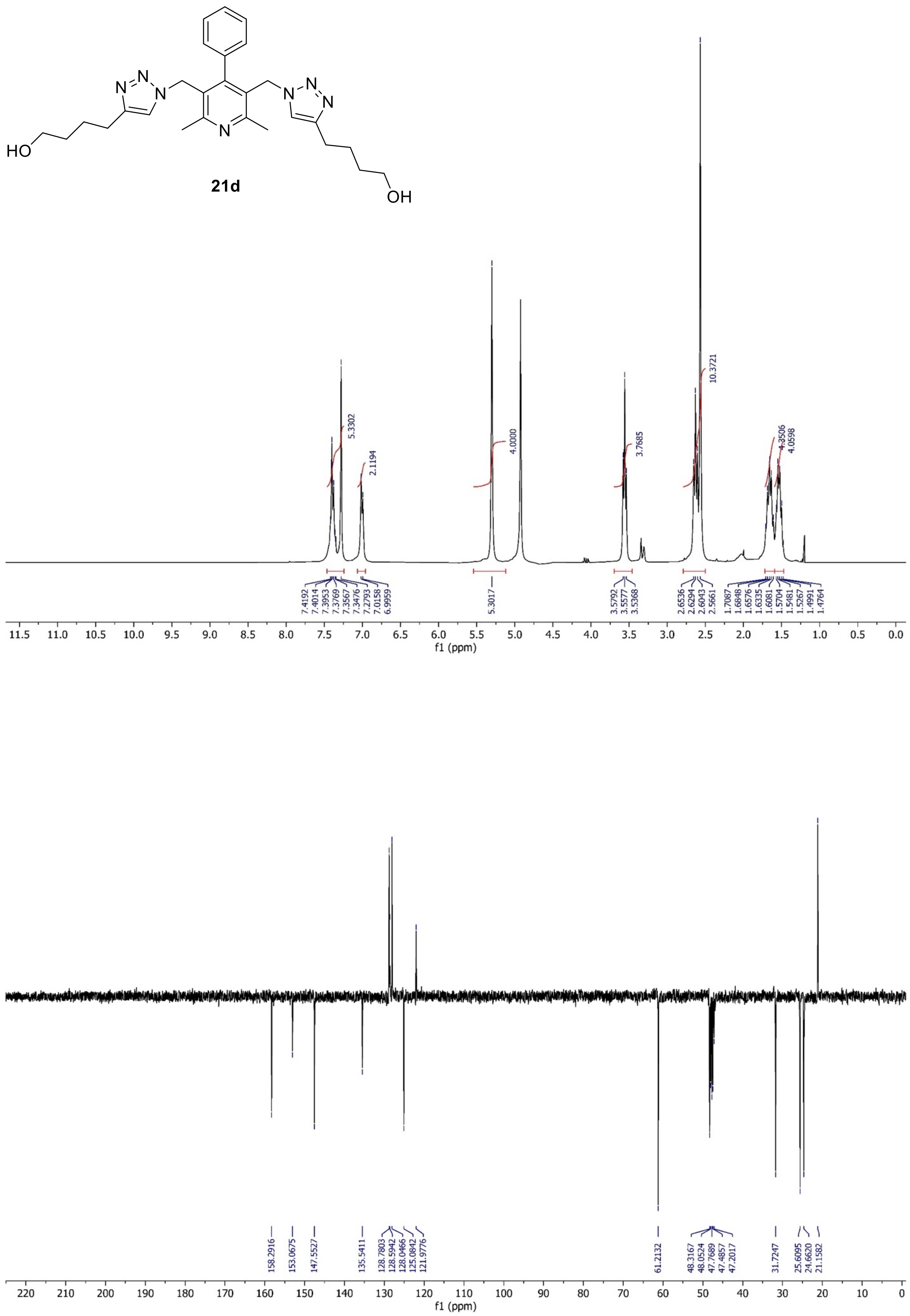

Figure S48. 1D ^{1}H (*top*) and ^{13}C APT (*bottom*) NMR spectra of compound **21d**.

References

1. Kushwaha, K.; Kaushik, N.; Lata; Jain, S.C. Design and synthesis of novel 2H-chromen-2-one derivatives bearing 1,2,3-triazole moiety as lead antimicrobials. *Bioorg. Med. Chem. Lett.* **2014**, *24*, 1795–1801, doi:10.1016/j.bmcl.2014.02.027.
2. Lv, X.; Ying, H.; Ma, X.; Qiu, N.; Wu, P.; Yang, B.; Hu, Y. Design, synthesis and biological evaluation of novel 4-alkynyl-quinoline derivatives as PI3K/mTOR dual inhibitors. *Eur. J. Med. Chem.* **2015**, *99*, 36–50, doi:10.1016/j.ejmech.2015.05.025.
3. Xue, Y.; Zhang, S.-S.; Cui, K.; Huang, J.; Zhao, Q.-L.; Lan, P.; Cao, S.-K.; Ma, Z. New polymethylene-based AB 2 star copolymers synthesized via a combination of polyhomologation of ylides and atom transfer radical polymerization. *RSC Adv.* **2015**, *5*, 7090–7097, doi:10.1039/C4RA14504F.
4. Dvorak, C.A.; Apodaca, R.; Xiao, W.; Jablonowski, J.A.; Bonaventure, P.; Dugovic, C.; Shelton, J.; Lord, B.; Miller, K.; Dvorak, L.K. Diamine-based human histamine H3 receptor antagonists: (4-Aminobutyn-1-yl)benzylamines. *Eur. J. Med. Chem.* **2009**, *44*, 4098–4106, doi:10.1016/j.ejmech.2009.04.049.
5. Karabiyikoglu, S.; Merlic, C.A. Transannular [4 + 2] Cycloaddition Reactions of Cobalt-Complexed Macrocyclic Dienynes. *Org. Lett.* **2015**, *17*, 4086–4089, doi:10.1021/acs.orglett.5b01984.
6. Cui, L.; Ye, L.; Zhang, L. Gold-catalyzed efficient synthesis of azepan-4-ones via a two-step [5 + 2] annulation. *Chem. Commun.* **2010**, *46*, 3351, doi:10.1039/c001314e.
7. Mizuno, A.; Kusama, H.; Iwasawa, N. Rhodium(I)-Catalyzed [4+1] Cycloaddition Reactions of α,β-Unsaturated Imines with Terminal Alkynes for the Preparation of Pyrrole Derivatives. *Angew. Chemie Int. Ed.* **2009**, *48*, 8318–8320, doi:10.1002/anie.200904402.
